# Condition-specific RNA editing in the coral symbiont *Symbiodinium microadriaticum*

**DOI:** 10.1371/journal.pgen.1006619

**Published:** 2017-02-28

**Authors:** Yi Jin Liew, Yong Li, Sebastian Baumgarten, Christian R. Voolstra, Manuel Aranda

**Affiliations:** King Abdullah University of Science and Technology (KAUST), Red Sea Research Center (RSRC), Biological and Environmental Sciences & Engineering Division (BESE), Thuwal, Saudi Arabia; University of Michigan, UNITED STATES

## Abstract

RNA editing is a rare post-transcriptional event that provides cells with an additional level of gene expression regulation. It has been implicated in various processes including adaptation, viral defence and RNA interference; however, its potential role as a mechanism in acclimatization has just recently been recognised. Here, we show that RNA editing occurs in 1.6% of all nuclear-encoded genes of *Symbiodinium microadriaticum*, a dinoflagellate symbiont of reef-building corals. All base-substitution edit types were present, and statistically significant motifs were associated with three edit types. Strikingly, a subset of genes exhibited condition-specific editing patterns in response to different stressors that resulted in significant increases of non-synonymous changes. We posit that this previously unrecognised mechanism extends this organism’s capability to respond to stress beyond what is encoded by the genome. This in turn may provide further acclimatization capacity to these organisms, and by extension, their coral hosts.

## Author summary

RNA editing is a mechanism that alters the nucleotide sequence of transcripts; edited transcripts are thus different from what is encoded in the genome. If the edits occur in coding regions, non-synonymous changes will generate additional protein diversity. Environmental stress is one of the many triggers for RNA editing, and evidence for its role in stress response is emerging. Here, we identified nuclear-encoded genes undergoing RNA editing in *S*. *microadriaticum*, a dinoflagellate symbiont of reef-building corals. When subjected to several common environmental stressors, the editing frequencies of some genes shifted in a consistent pattern that was specific to the applied stress. Within these genes, the predicted edits were predominantly non-synonymous in nature, suggesting that RNA editing is introducing genotypic variability beyond the organism’s haploid genome, extending its capability to respond to stress. Our data suggest that differential RNA editing is a process that contributes to the capacity of these algal symbionts to acclimate to environmental stress.

## Introduction

RNA editing is a collection of co- or post-transcriptional processes that produce RNA sequences that differ from their DNA templates (excluding mRNA splicing, capping and polyadenylation). These processes provide organisms with an additional layer of post-transcriptional control, and typically manifest as the production of tissue-specific protein isoforms [1], play a role in caste determination [2], or to adapt to a new environment [3]. In contrast to these well-characterised editing events, a potential role for RNA editing in response to short-term environmental change is just emerging. For instance, studies in human monocytes show specific induction of C-to-U edits in the human SDHB gene in response to hypoxic conditions [4]; in *Drosophila*, A-to-I edit frequencies have been shown to respond to heat stress [5]. However, for the latter, the changes were tightly linked to transcriptional silencing of the sole RNA editing enzyme encoded in the genome.

Dinoflagellates are a highly diverse group of protists that thrive in both freshwater and marine environments. As one of the major primary producers in the world’s oceans, they are an integral part of the marine food web [6]. Dinoflagellates of the genus *Symbiodinium* are best known for being a key symbiont in many marine invertebrates, including reef-building corals [7]. As such, they also contribute to the acclimatization potential of their host [8, 9]. In dinoflagellates, RNA editing has been described in organelles since the turn of this century. The combined work of [10–15] established the presence of base-substitution edits in mitochondrial *cox1*, *cob* and *cox3* genes; similarly, other studies [16–20, 75, 76] showed that numerous plastid-encoded genes are also subject to RNA editing. Evidence for editing on nuclear genes is, however, lacking.

## Results

### Widespread nuclear RNA editing in the *S*. *microadriaticum* transcriptome

We generated 16 RNA-seq libraries from algal cultures subjected to 4 different conditions: 3 bleaching-relevant stressors (cold, heat and dark) and a control condition. A total of 313 million paired-end reads were mapped to the *S*. *microadriaticum* nuclear genome in order to detect RNA editing in the transcriptome (S1 Table). As *S*. *microadriaticum* is a haploid organism [21], we focused on identifying genomic positions with unexpected base compositions in the RNA-seq data that significantly departed from the expected reference base. We applied five stringent filters to reduce false positives. Briefly, we removed edits that were deemed more likely to be sequencing errors, and further required the edit frequency to be between 5% and 95% (edit frequency of 0% = not edited; 100% = completely edited). We then removed edits located in sequence contexts that were not unique, in the vicinity of splice junctions, or on ambiguous genomic loci that had had DNA-seq reads with non-reference bases mapped to them (for details, see Materials and Methods).

**Fig 1:**
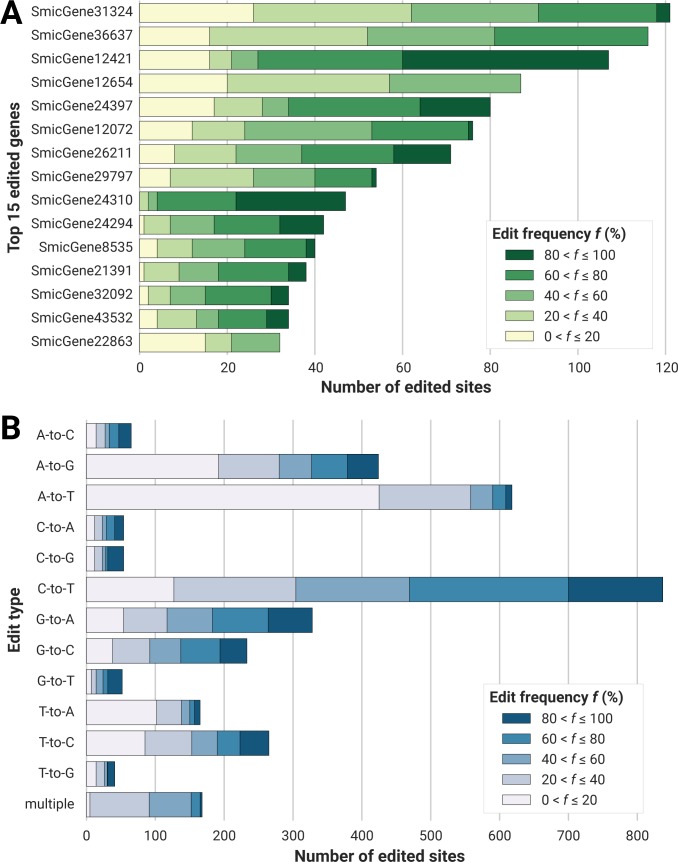
**Edited sites categorised by (A) 15 most frequently edited genes and (B) editing type.** Each bar was further subdivided into equal-width quintiles based on editing frequency. Lighter hues correspond to less frequently edited sites; darker hues to more frequently edited sites. For (B), sites with two or more editing events are lumped together in the "multiple" category.

This analysis identified 3,304 significantly edited sites (median coverage of 69×) in the *S*. *microadriaticum* nuclear genome (S2 Table). Out of the 3,304 sites, 2,547 (77.1%) occurred in 774 gene models (1.6% of all 49,109 gene models) enriched for genes that regulate metabolism, development and transport (S3 Table). The distribution of edits in genes was uneven: the top 15 genes accounted for 979 edits (Fig 1A), while the mode and median number of edits per gene were both 1. Taken together, these edited nuclear-encoded genes had 1 edited base per 1,000 bases, roughly 1–2 orders lower than that in dinoflagellate organelles (i.e. 1 edited base per 10–100 bases) [10, 11, 16, 17, 19]. We also identified 757 edits (22.9%) that resided in intergenic regions, potentially located within long non-coding RNAs or repetitive elements as shown recently [22, 23], or unannotated genic regions.

### All RNA editing types are present

We observed all combinations of N-to-N editing in the nuclear genome of *S*. *microadriaticum* (where N is any nucleotide base), unlike the specific A-to-I [24] and C-to-U [25] RNA edits prevalent in metazoans, or the expanded repertoire of changes observed in dinoflagellate organelles [10–14, 16–20, 75, 76]. Among the 12 possible RNA editing types, we observed more transitions than transversions (Fig 1B). This observation is similar to previous reports from dinoflagellate organelles (S4 Table), plant organelles [26], as well as non-A-to-G editing in humans [27]. This observation might be due to the conservation of RNA editing machineries across Eukarya. The distributions of edit frequencies within each edit type were fairly even, and the proportion of highly edited sites (edit frequency > 80%) in every edit type were similar (18.9% ± 3.6%, 1 SE) (Fig 1B).

### Distribution of RNA edits within genes is non-random

**Fig 2:**
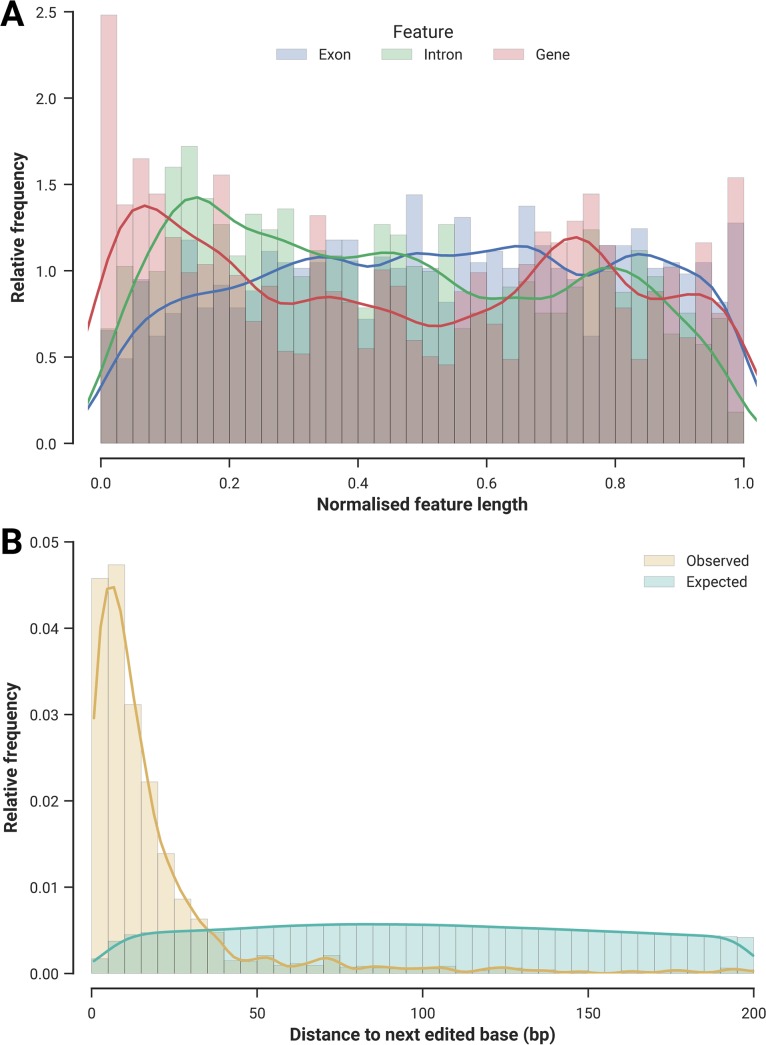
Distribution of edited sites. (A) Relative positions of the edited sites were tallied along the normalised lengths of exons (blue), introns (green) and genes (red). Each bar in the histogram covers a relative distance of 0.025. (B) Relative frequencies of distances to neighbouring edits. The observed distribution in *S*. *microadriaticum* (yellow) is contrasted with the expected distribution produced by 10,000 Monte Carlo simulations (cyan). Distributions were significantly different from each other (*p* < 10^−300^, χ^2^ test).

To further understand the spatial distribution of RNA edits, we focused on the 2,547 genic RNA edits to find out whether they exhibited positional tendencies. After normalising for the length of the edited genes, we found significantly more edits located at the 5’ end (9.7% of all edits are in the initial 5%, *p* < 10^−29^, binomial test) (Fig 2A, red bars and line). This tendency was a product of preferential editing close to the 5’ end of genes, since individual exons and introns displayed an even spread of editing events (Fig 2A, red vs. blue and green respectively). We also discovered that the edited sites clustered significantly (*p* < 10^−300^, χ^2^ test), i.e. they tended to be closer to each other than expected by chance (Fig 2B)—a similar trend is present in dinoflagellate organelles, and also in humans, as the bulk of ADAR-driven A-to-I edits are known to cluster tightly (termed “hyper-editing”) [28].

### Sequence motifs predict editing types

By utilising MEME [29], a motif identifier, we discovered several sequence motifs strongly associated with specific nucleotide conversions (S5 Table and S1 Dataset). In particular, we found that C-to-T, G-to-C and G-to-A editing sites harboured specific sequence motifs within a window of ±100 bp around the edited sites. Of note were motifs associated with C-to-T edits, which represented the best-scoring motifs derived from genic and exonic contexts with highly statistical significance (odds ratio > 15, *p*-value of < 10^−144^). These motifs were present in 41% and 73% of all C-to-T edits in genes and exons respectively (S1 Fig). These sequences were, however, different from sequence contexts around C-to-T edits in plant organelles [30].

### Condition-specific differential RNA editing

To test the intriguing possibility that *S*. *microadriaticum* uses condition-specific differential RNA editing as a mechanism of environmental acclimatization, RNA editomes from cultures subjected to three acute stressors (cold, heat and dark; illustrated as “16C”, “36C” and “DS” in S2 Fig) were compared against cultures grown under regular conditions (“control”). To increase statistical power, we selected genes with at least 2 edits present over all 4 replicates in each condition for the assessment via Generalised Linear Models (GLMs) (see Materials and Methods).

Out of 229 genes that passed our stringent filters, we identified 114 genes (50%) that are differentially edited in at least 1 stress condition relative to the control treatment (*p* < 0.05, S6 Table). This indicates that a large fraction of edited genes respond to different stressors by changing their editing patterns in a condition-specific manner. Enrichment analysis of these 114 genes indicates that proliferation- and stress-related proteins are prominent members among the differentially edited genes, supporting a function in stress response (S3 Table). Interestingly, 47 of the differentially edited genes responded to multiple stressors. Most genes (73 genes) were differentially edited under heat stress, and fewer genes were differentially edited under cold stress and dark stress (49 genes and 53 genes respectively, S6 Table).

**Fig 3:**
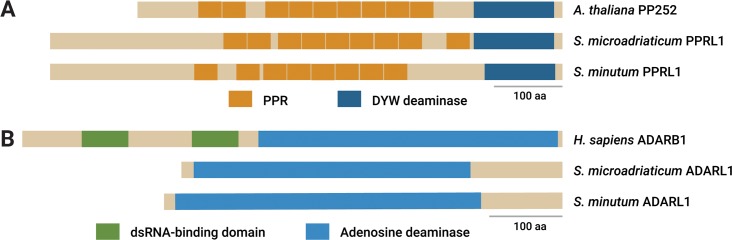
Protein domains of known RNA editing genes and candidate *Symbiodinium* homologues. For brevity, one representative sequence was chosen per species for (A) PPR-like and (B) ADAR-like proteins.

At this juncture, we assessed the extent of two confounding factors of the observed changes in editing frequencies. Firstly, the editing machinery itself might be responding to the stressors, as exemplified by a study of five drosophilid species [5]. We contrasted the changes in editing frequencies under each stressor against the “control” condition for all sites (lighter bars, S3 Fig) and sites within the 114 differentially edited genes (darker bars, S3 Fig). Contrary to the drosophilids’ general decrease in editing frequencies under heat stress, *S*. *microadriaticum* had roughly equal proportions of edited sites with increased and decreased editing frequencies under all three stressors (S4 Fig and S7 Table). This observation, we argue, is unlikely to arise from a general shutdown (or activation) of the editing machinery. To obtain further support, we analysed *Symbiodinium* transcriptomes for candidate homologues of known proteins involved in RNA editing in metazoans and plants. We identified two PPR-like (pentatricopeptide repeat) [31] and three ADAR-like (adenosine deaminases acting on RNAs) [32] candidates in the *S*. *microadriaticum* transcriptome [33]. Similarly, these candidates were also present in the *S*. *minutum* transcriptome [34], suggesting that these candidates are conserved on a genus-wide basis (Fig 3, S5 Fig and S6 Fig). By passing the same RNA-seq reads through a differential expression pipeline, we were unable to detect differential expression of any of the five candidate RNA editing genes under any stressor (S8 Table).

The second confounding factor arises from the differential stability of edited transcripts (relative to their unedited versions), exemplified by a study that reported the increased stability of ADAR-edited transcripts in humans [35]. Increases in editing frequency might thus be a side-effect of edited transcripts having a longer half-life in the cytosol. The current dearth of molecular tools in *S*. *microadriaticum* prevents us from replicating the same verification process used in that study. However, our differential expression analysis shows that the overall proportion of differentially expressed genes amongst all genes (2.4%) is neither significantly different from that of all edited genes (1.7%, Fisher’s exact *p* = 0.23) nor differentially edited genes (1.8%, Fisher’s exact *p* = 1) (S8 Table). This suggests that the differential editing of transcripts is largely independent of the differential stabilities of the edited transcripts, as the expression levels of edited genes are largely unchanged under stress.

**Fig 4:**
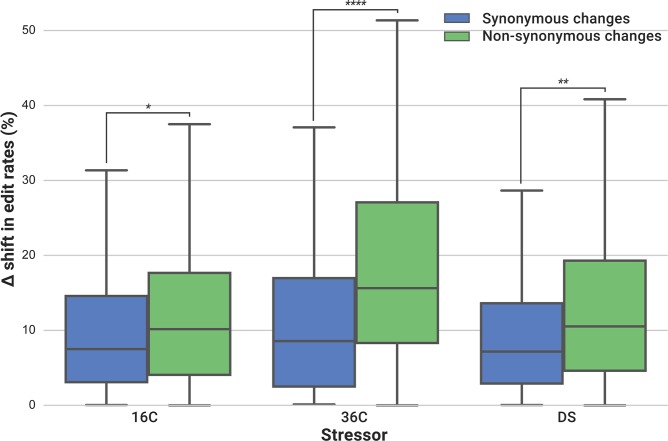
Non-synonymous changes predominate in coding regions of differentially edited genes. Delta shifts were calculated as the absolute difference of means between stressed and control samples (*n* ≥ 3 per condition). Shifts in edit frequencies at positions that lead to non-synonymous changes (green) are significantly higher than synonymous ones (blue) for all tested stressors: cold (“16C”), heat (“36C”) and dark (“DS”). *p*-values were computed from inter-group two-tailed t-tests (*: *p* < 0.05; **: *p* < 0.01; ****: *p* < 0.0001).

In line with the idea that RNA editing plays a role in stress acclimatization, we found that differentially edited genes also displayed significantly more edits resulting in non-synonymous changes (*p* < 10^−11^), and significantly less edits in intronic regions (*p* < 10^−12^) when compared to all edited genes (Table 1). Within coding regions of differentially edited genes, non-synonymous changes occurred at higher frequencies than synonymous changes (Fig 4) providing further indication that differentially edited genes have a propensity for edits that lead to changes in amino acid sequence.

**Table 1:** Distribution of RNA edits in condition-specific and all differentially edited genes. Intergenic edits are not considered; sites with multiple editing events are considered as separate effects. Category labels are as reported by SnpEff, and p-values were calculated using Fisher’s exact test adjusted for multiple testing (Benjamini-Hochberg).

	Condition-specific differentially edited genes		All genic edits		
Edit effect	#	%	#	%	*p*-value
5' UTR	59	3.7%	63	2.4%	0.04
Gain of start codon	12	0.7%	14	0.5%	0.63
Loss of start codon	0	0.0%	1	0.0%	1.00
Synonymous coding	176	11.0%	263	9.9%	0.50
Non-synonymous coding	692	43.1%	859	32.3%	8.83 × 10^−12^
Intron	658	41.0%	1,408	53.0%	2.12 × 10^−13^
Gain of stop codon	7	0.4%	13	0.5%	1.00
Loss of stop codon	0	0.0%	1	0.0%	1.00
3' UTR	2	0.1%	34	1.3%	4.49 × 10^−5^

**Fig 5:**
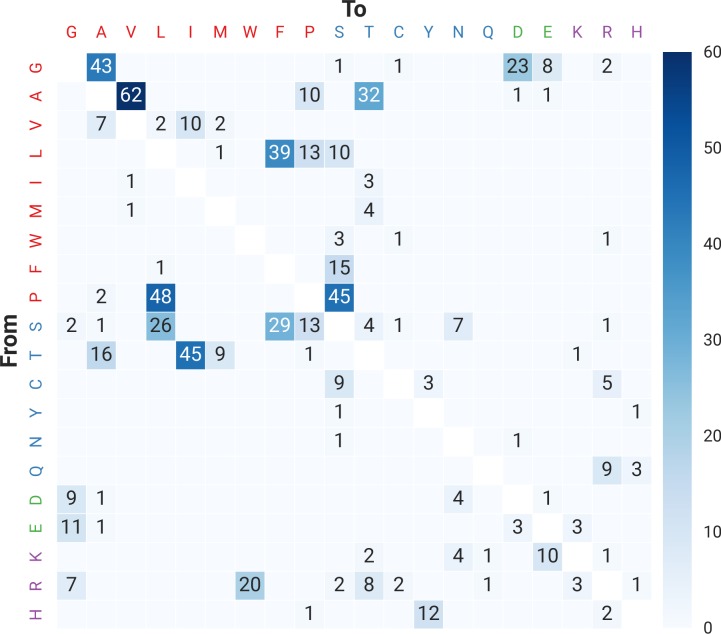
Heatmap of non-synonymous amino acid changes of differentially edited genes. Original amino acid is on the y-axis, while the post-editing amino acid is on the x-axis. Amino acids are represented as single-letter codes, and coloured according to the property of their side chains: non-polar (red), polar uncharged (blue), acidic (green) and basic (purple).

For non-synonymous edits, the most prevalent changes tended to be from a non-polar side chain to another one (e.g. there were 62 arginine-to-valine and 48 proline-to-leucine). However, we also found many other changes that are likely to alter the structure of the protein. For example, there were 45 instances of the non-polar proline substituted by the polar uncharged serine; and 10 instances of the basic lysine altered to the acidic glutamate (Fig 5).

**Fig 6:**
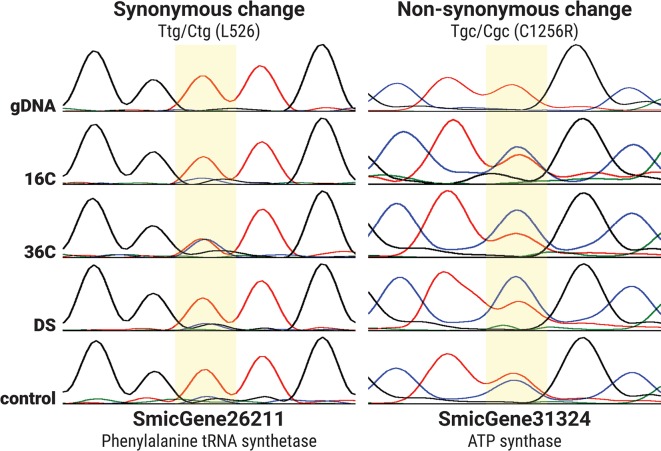
Cycle sequencing peak traces confirming differential RNA editing across different stressors. For brevity, one replicate per condition, and one edit leading to a synonymous change (left) and non-synonymous change (right) are shown. Traces for A, C, G and T are coloured green, blue, black and red respectively. Edited sites are highlighted in yellow; downstream effects of the edits are labelled on top of the highlight (i.e. “Ttg/Ctg” implies a T-to-C edit in the first codon; “C1256R” denotes the non-synonymous change of cysteine to arginine at position 1256 of the amino acid sequence).

To unequivocally confirm that transcripts are edited in a condition-specific manner, we designed PCR primers targeting five genes that were identified as differentially edited under stress. The PCR was performed on cDNA samples and genomic DNA to exclude the presence of un-sequenced genomic regions with similar sequences (n = 154 electropherograms, S3 Dataset). Sanger sequencing results of these amplicons confirmed the condition-specific changes of RNA editing in those genes: 239 of 375 (63.7%, binomial *p* < 10^−6^) edited sites had PCR results tallying with the directional shift in editing under stress as observed in the RNA-seq results (Fig 6, S7 Fig and S9 Table). Almost all (97.6%) of the primary peaks detected in the PCR traces corresponded to either the genomic base or the edited base—the former occurring when edit frequencies were lower; the latter occurring when the edit frequencies were higher.

## Discussion

In this work, we are the first to report RNA editing in genome-encoded transcripts of a dinoflagellate, which supplements similar observations made for dinoflagellate organellar genomes. Unlike metazoans and plants, where the vast majority of edits are A-to-I and C-to-U respectively, there is a greater variation of edit types in dinoflagellates. Previous work in dinoflagellate organelles reported all edit types are possible; similarly, in our data, we could observe all N-to-N edit types. These edit types were not distributed uniformly: the most frequently edited type (C-to-T) was approximately 20-fold more common than the rarest edit type (T-to-G). This is somewhat similar to previous reports from dinoflagellate organelles, where C-to-T, A-to-G and T-to-C edits were among the most frequent; however, exceptions such as A-to-T (19% in *S*. *microadriaticum* genome, 0–3% in organelles) were evident as well. While the presence of all edit types and overall spread of edit type frequencies suggest that the editing machinery might be shared between the host and its organelles, the exceptions would indicate that the latter utilises a subset of the RNA editing machinery available, or perhaps the more restricted sequence contexts in these organelles strongly favour certain edits and disfavour others.

A confounding factor to the observed condition-specific RNA editing was the dysregulation of the editing machinery, as observed in *Drosophila* [5]. To assess that possibility, we performed *in silico* identification of candidate *S*. *microadriaticum* RNA editing proteins (PPR-like and ADAR-like) via sequence similarity. While these candidates appear contentious, especially since ADARs are thought to be restricted to metazoans [32], further *in vivo* verification of these candidates is stymied by the dearth of reliable molecular techniques, e.g. transformation and gene knockdowns in dinoflagellates. Regardless, two observations indirectly suggest that some of these deaminases retained similar functionality in *S*. *microadriaticum*. Firstly, edited sites in nuclear-encoded genes of *S*. *microadriaticum* appear more clustered than expected, similar to edits in dinoflagellate organelles [36] and A-to-I edits in humans [28]. Secondly, sequence motifs have been reliably discerned in the vicinity of C-to-T edits. These motifs are, however, unlike any that have been identified in metazoans [37–39] or plants [30, 40], perhaps due to the evolutionary distance of dinoflagellates from metazoans and plants [41].

Based on our data, we argue that *Symbiodinium* is able to shift its RNA editome in response to environmental stress. The 114 genes that we identified in *S*. *microadriaticum* were differentially edited in response to at least one of three bleaching-relevant stressors: cold, heat or dark. This subset of genes had significantly more non-synonymous amino acid changes, and our observations are in line with the non-synonymous change in a K^+^ channel of the octopus *Pareledone sp*. that allows it to adapt to colder waters [3]. The prevalence of non-synonymous edits has similarly been demonstrated in other organisms such as *Drosophila* [22] and the squid *Doryteuthis pealeii* [42]. For non-synonymous mutations, larger structural or functional changes are possible through the introduction of amino acids with very different properties. This translational flexibility may represent a previously unrecognised mechanism of acclimatization that provides additional phenotypic plasticity to the organism, and thereby, increases its ability to respond to environmental change in comparison to the genome-encoded gene product [43].

As more research is done on dinoflagellate biology, it becomes more and more apparent that dinoflagellates do not seem to play by the same rules as metazoans or plants. Few genes are commonly observed to respond to stress with a change in expression [44–46], possibly linked to the relative paucity of proteins harbouring transcription factor domains [47]. Rather, expression profiles seem to be fixed between different clades, species, or even strains within species, irrespective of physiological conditions [45, 48]. Our data concurred with these observations: roughly 2% of all genes are differentially expressed under any of the three stressors, and this proportion is independent of editing state.

How, then, does the organism respond to stress? One possible answer to this conundrum is the use of post-transcriptional control mechanisms, previously illustrated through the identification of an RNAi machinery and a diverse set of miRNAs in two *Symbiodinium* species [44, 49]. We suggest that RNA editing is an additional genome-wide, post-transcriptional mechanism that modulates gene expression through shifts in the RNA editome.

What, then, would be the biological purpose of RNA editing in *Symbiodinium*? We postulate that RNA editing serves a dual function—firstly, it serves to regulate gene expression on a genome-wide level, as evidenced by a substantial number of *S*. *microadriaticum* genes (774 genes, 1.6% of all genes) being edited. Within genes, edited sites have a propensity to be at the 5’ end of genes. Furthermore, we find that differentially edited genes that respond to environmentally relevant stressors show higher frequencies of non-synonymous changes. These observations might be tightly linked to the biological functions of these edits. Synonymous changes might affect gene expression via codon bias [50]; non-synonymous changes at the N-terminus signal peptide region will disrupt protein localisation, while changes in structural regions could affect the stability of the peptide. It is also likely that these edits exert larger effects when they are closer to the 5’ end of the gene, and our data substantiates this postulate: the initial 5% of the gene contains 126 of 859 (14.2%) non-synonymous changes; comparatively, 26 of 263 (11.2%) synonymous edits are in the same region. Secondly, RNA editing putatively provides a low evolutionary cost system that introduces additional genetic variation above the coding capacity of the genome on which selection can act without the risk of generating lethal variations. This is especially important as *Symbiodinium* is haploid in the vegetative state [21], thus deleterious mutations are more likely to be costly.

In conclusion, it is a challenging prospect to identify true edits in an organism with a draft genome and unclear mechanistic origins of these edits. However, our conservative comparative transcriptomics of cultures under bleaching-relevant stressors have revealed *bona fide* RNA edits in the nuclear genome of *S*. *microadriaticum*. Our work highlights a novel role for RNA editing in providing a mechanism for dinoflagellates—and, by extension, their coral hosts—to acclimatize, and potentially adapt to, changing environments.

## Methods

### Growth conditions and experimental treatments of *S*. *microadriaticum*

12 independent *S*. *microadriaticum* cultures were grown in 200 ml of f/2 media (Sigma-Aldrich, St. Louis, MA) for 2 weeks in non-treated Nunclon Δ TripleFlasks (Thermo Scientific, Waltham, MA) at 26°C. The cultures were illuminated at a constant irradiance of 250 μmol photons m^-2^·s^-1^ on a 12h:12h day:night light-cycle (daytime of 6 am–6 pm). Prior to the day where cells were collected, three bottles were wrapped in foil (“DS”: dark stress for 6 hours); three bottles were placed in an incubator programmed to ramp up to 36°C at 8 am (“36C”: extreme heat stress for 4 hours); and another three were placed in an incubator programmed to cool down to 16°C at 8 am (“16C”: extreme cold stress for 4 hours). The remaining three bottles, labelled as “control”, were not subjected to additional stresses (S2 Fig). Cells were pelleted in 50 ml Falcon tubes at 500*g*, decanted, and washed once by resuspending it with autoclaved, filtered seawater, and re-pelleted at 1,000*g*. The drip-dried pellet was snap-frozen in liquid nitrogen in preparation for RNA extraction.

### mRNA sequencing and transcriptome

Total RNA from *S*. *microadriaticum* was extracted by grinding the cell pellets with 200–300 μl of 0.5 mm glass beads (BioSpec Products, Bartlesville, OK) in liquid nitrogen-chilled mortar and pestle prior to using the RNeasy Plant Mini Kit (Qiagen, Hilden, Germany). The quality of extracted total RNA was assessed using a Bioanalyzer 2100 (Agilent, Santa Clara, CA). Strand-specific RNA-seq libraries were generated from oligo-dT selected total RNA using the NEBNext Ultra Directional RNA Prep Kit (New England Biolabs, Ipswich, MA) according to manufacturer's instructions. A total of 397 million paired-end read pairs (read length: 101 bp, insert size: 180 bp) were retrieved from 4 lanes on the HiSeq 2000 platform (Illumina, San Diego, CA).

### Identification of significantly edited sites

The pipeline used to identify edited sites is summarised in S8 Fig. Prior to mapping, sequence adapters and low quality ends were trimmed using trimmomatic [51], resulting in 256 million reads. The first and last 6 bp of each read were trimmed to remove potential primer bias during library generation. Mapping was carried out with Bowtie2 [52] (“--fr --mp 1,1 --score-min L,0,-0.08 --end-to-end --very-sensitive”). We refrained from using a gapped-read mapper that maps across introns, as it led to many reads preferring to have artificially long “introns” to better match the reference genome downstream, potentially removing true edit locations if the read matched contiguously. The mapping efficiency is lower with a contiguous read mapper, but we are more confident that mapped reads have had the correct provenance assigned. Post-mapping, reads were de-duplicated on a per-replicate basis, to avoid PCR amplification biases influencing editing frequencies. Significantly edited sites were identified across all stress conditions by combining the post-processed FASTQ sequences from this experiment (three biological replicates from four treatments) and another previously published dataset [44] (one biological replicate, identical set of four treatments) into one file. The merged file was analysed using REDItools [53] with several non-default parameters (-T 9-9 -e -W) in order to avoid potential biases in edit calling [54–58].

Previously, sequencing errors have been shown to be concentrated on the ends of the sequenced reads [56]. To reduce potential biases from sequencing errors, the first and last 9 bases were trimmed of each read from further processing using the “-T 9-9” parameter in REDItools (see S9 Fig for error rates from both ends). Non-uniquely mapped reads (“-e”) were excluded to prevent reads mapping to paralogs; substitutions in homopolymeric regions (“-W”) were also removed as it is hard to distinguish between an insertion (resulting from technical artefacts) and a substitution (possible *bona fide* editing) in these regions.

REDItools reports the number of reads that are matches and mismatches to the genomic sequence for each transcribed genomic position, and implements an algorithm to calculate the *p*-value of each site being significantly edited. However, we noticed that the resulting *p*-values clustered around certain values (0.25, 0.5, 0.75, 1) instead of exhibiting a continuous distribution. We therefore decided to calculate the *p*-values based on the statistical principles outlined in [2]. Briefly, we modelled the distribution of editing errors as a binomial distribution B(*n*, *p*), where *n* = total coverage of that site in question and *p* = 0.01 (corresponding to a Phred score of 20). We opted to use the maximum possible sequencing error rate instead of the average error rate, as we preferred to be more conservative in our cutoffs, trading sensitivity for accuracy. Thus, the *p*-value for a site with *k* edits in a total coverage of *n* reads was calculated as the probability of observing at least *k* edited reads in that site. The resulting *p*-values were corrected for multiple testing [59, 60], and sites with post-corrected *p* < 0.01 were considered to be significant.

Post-multiple testing correction, we introduced five additional filtering criteria to address other potential biases. Firstly, sites that were edited below 5% and above 95% were removed. The former removes lowly edited sites that would be of less interest, while the latter guards against mis-sequenced bases in the reference genome (and also acts as a 5% filter even if the genomic position was not sequenced correctly). A minority of sites (5%) were reported to be edited to multiple bases. In order to separate *bona fide* multiple edited sites from those that might arise from sequencing errors, we required sites with multiple edits to have ≥ 20% overall editing frequency and ≥ 2 reads supporting each of the particular editing event.

Secondly, we observed that spliced reads do map contiguously to another locus with less sequence similarity, as that locus lacks the intron spanned by the exons present in the spliced read (the same read is unable to map across that intron in the actual locus), giving rise to edited sites that are false positive. To remove these, a window of 201 bp (100 bp upstream and downstream) centred on each edited site was subjected to a BLASTN search against the genome. If the window matched another locus (e-value of ≤ 10^−5^), the site was removed from further consideration. We believe that this step is crucial for dinoflagellate genomes, as these genomes tend to contain many tandem repeats [61–63].

Thirdly, edited sites that were overrepresented on reads in a particular direction (> 85%) were removed—these sites mostly arose from spliced reads mapping into intronic regions in a unidirectional manner [54].

Furthermore, edited sites that fell into the first and last 9 bp of annotated introns were removed. This filter stems from the observation that the first few bases of introns tend to match the 5’ end of the next downstream exon, while the last few bases of introns closely match the 3’ end of the immediately upstream exon [61], producing false edited sites when spliced reads map into introns and the corresponding ends stop being similar.

Lastly, to further discount the possibility of edits resulting from SNPs or sequencing mistakes in the genome, eight paired-end DNA-seq libraries from *S*. *microadriaticum* (~823 million read pairs) were mapped against the genome with mapping parameters that mirrored that of the RNA-seq reads. We then opted to be extremely stringent by only permitting unambiguous genomic loci past the filter.

We noticed that REDItools reported RNA editing events with the implicit assumption that the gene models were on the Watson strand of the genome. Based on the gene models for the *S*. *microadriaticum* genome [33], edits that occurred in genes on the Crick strand were reverse-complemented via a script; edits that occur in genes on the Watson strand or intergenic regions were left as-is.

### Clustering of RNA editing positions

We observed that the edited sites tended to be located in clusters instead of being randomly distributed within the gene. To support to this observation, for every gene that contained at least two significantly edited positions, we recorded the average distance of each edit to its immediate neighbours (upstream and downstream) in the same gene. If the edit was the most upstream or most downstream one in the gene, the distance to the nearest edit was recorded instead.

We contrasted this observed distribution to a null distribution of distances, which was produced by shuffling the positions of the edits randomly within the same gene without changing the number of edits in the gene. The Monte Carlo shuffling was repeated 10,000 times and subsequently averaged out to obtain an “expected distribution” of neighbouring distances.

A χ^2^ test was performed on the observed and expected distributions to ascertain whether the distributional differences were statistically significant.

### Identification of RNA editing motifs

In order to discover motifs that are strongly associated with RNA editing in *S*. *microadriaticum*, we generated training sets for the motif based analysis tool MEME [29]. While the RNA editing machinery has been well-described in plants and metazoans [31, 64], these mechanisms do not explain many of the other edit types that we observe in *S*. *microadriaticum*. As the editing machinery is unknown to us at present, we ran motif searches on genic sequences (to mimic co-transcriptional editing) or exonic sequences (to mimic post-transcriptional editing).

Briefly, for each possible edit type (e.g. A-to-G, A-to-C, etc.), we extracted sequence contexts of 100 bp upstream and downstream of positions with the specific edit type (i.e. each window is 201 bp). Half of these sequences were randomly chosen as the training set while the other half served as the test set. In order to achieve a cleaner signal from the training set, sequence contexts containing more than one edit were moved to the test set. Potential motifs governing the specificity of the respective editing types were identified with MEME (“-dna -mod zoops -minw 6 -maxw 50 -nmotifs 6 -minsites 50”) with the default e-value cutoff of 0.05. Using MAST, true positives were calculated from checking for the presence of these motifs in the test set (*p* < 10^−4^); false negatives were obtained from testing against 10,000 randomly retrieved sequence windows (201 bp) from genes with no editing events (*p* < 10^−4^).

### Identification of differentially edited genes

Differentially edited genes are defined as genes that exhibit significant shifts in edit frequencies in some or all edited positions when subjected to stress relative to the “control” treatment. These genes were identified by fitting generalised linear models (GLMs) in R on a per-gene basis with the following formula:

glm(edited,non_edited∼growth_condition*position,family='binomial')

where “edited, non_edited” refer to a two-column response variable containing the number of reads with and without RNA editing respectively at a particular position; “growth_conditions” and “positions” refer to predictor variables that stored the growth condition and the location of the edited base as factors. Data from each replicate were entered as individual rows instead of being pooled together, as we preferred to assign equal weightages to replicate observations (if the data was pooled, the replicate with the most mapped reads will have a bigger influence on the overall editing frequency). To minimise false positives, we filtered for genes containing ≥ 2 edit positions that have had reads mapping to these positions in all replicates.

This analysis was repeated for each stress condition (“16C”, “36C” and “DS”) relative to the “control” condition, with every gene having a *p*-value representing the probability that the difference in editing patterns across the four replicates of the stress condition relative to the four replicates of the control condition was due to chance (i.e. *p* approaches 0 if the editing frequency under stress were consistently different from control; *p* approaches 1 if editing frequency under stress mimics that of control). These *p*-values were corrected for multiple testing [59, 60], and genes with corrected *p* < 0.05 were considered differentially edited in response to stress.

### Identification of candidate RNA editing proteins

To identify proteins that are putatively involved in the RNA editing of *Symbiodinium*, known reference proteins from both metazoans and plants were retrieved from the UniProt SwissProt database [65] (S2 Dataset). Reference transcriptomes of *S*. *microadriaticum* [33] and *S*. *minutum* [34] were translated into protein sequences using TransDecoder (https://transdecoder.github.io) [66]. These open reading frames were then queried against reference RNA editing proteins using BLASTP (e-value ≤ 10^−5^). For matches below the cutoff, putative Pfam protein domains were annotated via HMMER (http://hmmer.org); transcripts were only retained if they contained crucial protein domains with RNA editing activity. Protein alignments of known RNA editing domains were generated with Jalview [67], and coloured with the Clustal X Colour Scheme (http://www.jalview.org/help/html/colourSchemes/clustal.html), which assigns the same colour to amino acids of similar side-chain properties. Amino acids are only coloured if the per-site conservation is above a certain threshold.

### Identification of differentially expressed genes

Similar to the identification of edited sites, trimmed RNA-seq reads were mapped against all *S*. *microadriaticum* gene models and eight candidate RNA editing homologues with kallisto v0.42.4 [68] using default parameters. Differentially expressed genes were subsequently identified using sleuth [69].

### Functional analysis of genes with edited sites

To discern potential functional differences between the set of condition-specific edits and the overall set of edits, we used SnpEff [70] to annotate potential changes in our gene models that result from the respective sets of edits. For each editing location (and for each type of edit in sites with multiple editing types), SnpEff identifies the region where the edit lies in (intergenic, intronic, exonic) and the effect of the edit (e.g. synonymous substitutions, non-synonymous substitution, gain of stop codons etc.).

### GO term enrichment

Functional enrichment analyses were performed using topGO (version 2.16.0) [71]. We used the default topGO "weight01" settings, and considered terms that had a *p*-value of < 0.05 as significant. The resulting *p*-values were not corrected for multiple testing as non-independent tests are carried out on each GO term by topGO [71].

### Verification of edited genes

As the genome of *S*. *microadriaticum* is not complete, there is still a possibility that similar transcripts produced from unsequenced genomic regions will map to existing regions, and mismatched bases will be considered as false RNA edits. In order to provide additional verification that genes are edited, and also to qualitatively confirm the editing patterns in differentially edited genes, PCR primers were designed to target seven amplicons (each ~300 bp containing ~20 edits) in five genes (one amplicon for SmicGene12072, SmicGene12421 and SmicGene26211; two amplicons for SmicGene31324 and SmicGene36637). Regions containing edits that exhibited large differences in edit frequencies between stressed samples and controls were preferentially selected. We took exceptional care to avoid designing primers on top of other known edited sites, which would result in PCR amplification biases if edits tended to occur in tandem. To optimise amplification, a script was written to incorporate other considerations e.g. similar melting temperatures, similar GC%, and avoiding long stretches (≥ 5) of the same nucleotide, allowing us to brute-force primer combinations with Primer3 (v2.3.6) [72] to find primers satisfying all desired criteria.

Leftover RNA from library construction (3 replicates from “16C”, 2 from “36C”, 2 from “DS” and 3 from “control”, i.e. 10 in total) were reverse-transcribed with SuperScript III First-Strand Synthesis SuperMix (Invitrogen, Carlsbad, CA) according to manufacturer’s instructions. The cDNA mix was subjected to 40 cycles of PCR (with a melting temperature of 60°C), loaded into a 1% agarose gel, and bands of the correct size were gel purified using QIAquick MinElute kit (Qiagen, Hilden, Germany) as per manufacturer’s instructions.

To provide further support that RNA editing has occurred, the same amplicons were used to amplify genomic DNA extracted from a separate *S*. *microadriaticum* culture with the same genotype. PCR amplification with the same settings were carried out, and gel purified in the same manner. Sanger sequencing on all samples were performed in-house (KAUST Bioscience Core Lab). Per-position peak height ratios from the AB1 traces produced from Sanger sequencing was obtained using a manufacturer-provided online tool [73].

### Accession numbers

Short read data generated for this study can be retrieved from the NCBI Short Read Archive (SRA), with the following BioSample accession numbers: SRS429458, SRS429460, SRS429463, SRS429465 and SRR3337493–3337505, or BioProject accessions of PRJNA205322 and PRJNA315758. Further detail is provided in S1 Table. The extent of RNA editing can be visualised on a genome browser, available at http://smic.reefgenomics.org [74].

## Supporting information

S1 Fig Motifs identified by MEME in sequence contexts surrounding edited sites. (A) motifs that are highly significant (Benjamini-Hochberg corrected *p* < 10^−40^, odds ratio > 10) are listed here. (B) Highest-scoring motifs from C-to-T, G-to-C and G-to-A edits. “Genic” and “exonic” refers to the sequence context used in deriving the motifs.
(TIF)

S2 Fig Illustration of experimental schematic. Number of replicates are denoted in the top-right corner of the flasks. Unless noted otherwise, cultures were grown at 26°C.
(TIF)

S3 Fig Changes in edit frequencies (relative to “control”) in response to stress. The number of sites (on the x-axis) that underwent increases in edit frequencies are in red; decreases are in green. The trends between all edited sites (pale green/red) is contrasted against the set of 114 differentially edited genes (dark green/red). In contrast to cold stress (“16C”) and dark stress (“DS”), there is a noticeable preference for increased editing in dealing with heat stress (“36C”).
(TIF)

S4 Fig Distribution of per-base changes in edit frequencies (relative to “control”) in response to stress. Positive values along the x-axis indicate an increase in edit frequencies under stress; negative values indicate decrease in edit frequencies.
(TIF)

S5 Fig Graphical alignment of the DYW domain in PPRs. Candidate homologues from *S*. *microadriaticum* (“Smic”) and *S*. *minutum* (“Smin”) were aligned against known proteins from *A*. *thaliana*. Amino acids are coloured according to the Clustal X colour scheme in Jalview.
(TIF)

S6 Fig Graphical alignment of the adenosine deaminase domain in ADARs. Candidate homologues from *S*. *microadriaticum* (“Smic”) and *S*. *minutum* (“Smin”) were aligned against known proteins from *H*. *sapiens*. Amino acids are coloured according to the Clustal X colour scheme in Jalview.
(TIF)

S7 Fig Additional PCR electropherograms for the five genes tested. For the genes (A) SmicGene12072, (B) SmicGene12421, (C) SmicGene26211, (D) SmicGene31324 and (E) SmicGene36637, two additional traces with several edited bases in windows of 15–30 bp were compiled. Traces for A, C, G and T are coloured green, blue, black and red respectively. Edited sites are highlighted in yellow.
(TIF)

S8 Fig Flowchart of applied analysis pipeline to identify significantly edited sites. At every stage of the pipeline, the number of sites passing all upstream filters are denoted in the light-blue boxes.
(TIF)

S9 Fig **Histograms showing per-position error rates from the (A) 5' end and (B) 3' end respectively.** The first 9 bases from both ends have far more mismatches than following bases, which—if included in our analyses—would produce numerous false positives.
(TIF)

S1 Table Read counts of RNA-seq libraries. (XLSX)

S2 Table List of 3,304 significantly edited positions in the *Symbiodinium microadriaticum* nuclear genome. (XLSX)

S3 Table Functional enrichment within (A) 774 genes with edited sites and (B) 114 differentially edited genes.
(XLSX)

S4 Table Summary of editing frequencies in dinoflagellate organelles from previous studies. (XLSX)

S5 Table MEME output for exonic context and genic context. (XLSX)

S6 Table *Symbiodinium microadraticum* genes differentially edited in at least one stressor. (XLSX)

S7 Table Change in editing frequencies when cultures are subject to stress, relative to control, for edited sites. Changes for (A) all genes and (B) genes that are differentially edited in at least one stressor are tabulated separately.
(XLSX)

S8 Table Summary and statistics for expression values in (A) edited genes and (B) all genes.
(XLSX)

S9 Table PCR verification for condition-specific differential editing. (A) shows a summary of the full results elaborated in (B).
(XLSX)

S1 Dataset Motif logos produced by MEME for all 12 edit types in both genic and exonic contexts. (GZ)

S2 Dataset FASTA files of Swiss-Prot protein sequences of known RNA editing proteins used to identify homologues in the *S. microadriaticum* and *S. minutum* transcriptomes. (GZ)

S3 Dataset AB1 trace files produced from Sanger sequencing that confirms condition-specific RNA editing. (GZ)

## References

[1] Chen SH, Li XX, Liao WS, Wu JH, Chan L. RNA editing of apolipoprotein B mRNA. Sequence specificity determined by in vitro coupled transcription editing. J Biol Chem. 1990;265(12):6811–6. 2324099

[2] Li Q, Wang Z, Lian J, Schiott M, Jin L, Zhang P, et al Caste-specific RNA editomes in the leaf-cutting ant Acromyrmex echinatior. Nat Commun. 2014;5:4943 10.1038/ncomms5943 25266559 PMC4200514

[3] Garrett S, Rosenthal JJ. RNA editing underlies temperature adaptation in K+ channels from polar octopuses. Science. 2012;335(6070):848–51. 10.1126/science.1212795 22223739 PMC4219319

[4] Baysal BE, De Jong K, Liu B, Wang J, Patnaik SK, Wallace PK, et al Hypoxia-inducible C-to-U coding RNA editing downregulates SDHB in monocytes. PeerJ. 2013;1:e152 10.7717/peerj.152 24058882 PMC3775634

[5] Rieder LE, Savva YA, Reyna MA, Chang YJ, Dorsky JS, Rezaei A, et al Dynamic response of RNA editing to temperature in Drosophila. BMC Biol. 2015;13:1 10.1186/s12915-014-0111-3 25555396 PMC4299485

[6] Taylor FJR. The biology of dinoflagellates. 1987.

[7] Stat M, Carter D, Hoegh-Guldberg O. The evolutionary history of Symbiodinium and scleractinian hosts—symbiosis, diversity, and the effect of climate change. Perspect Plant Ecol Evol Syst. 2006;8(1):23–43.

[8] Howells E, Beltran V, Larsen N, Bay L, Willis B, Van Oppen M. Coral thermal tolerance shaped by local adaptation of photosymbionts. Nat Clim Chang. 2012;2(2):116–20.

[9] Hume BC, Voolstra CR, Arif C, D'Angelo C, Burt JA, Eyal G, et al Ancestral genetic diversity associated with the rapid spread of stress-tolerant coral symbionts in response to Holocene climate change. Proc Natl Acad Sci U S A. 2016; 113(16)4416–4421. 10.1073/pnas.1601910113 27044109 PMC4843444

[10] Lin S, Zhang H, Spencer DF, Norman JE, Gray MW. Widespread and extensive editing of mitochondrial mRNAS in dinoflagellates. J Mol Biol. 2002;320(4):727–39. 12095251 10.1016/s0022-2836(02)00468-0

[11] Zhang H, Lin S. Mitochondrial cytochrome b mRNA editing in dinoflagellates: possible ecological and evolutionary associations? J Eukaryot Microbiol. 2005;52(6):538–45. 10.1111/j.1550-7408.2005.00060.x 16313447

[12] Jackson CJ, Norman JE, Schnare MN, Gray MW, Keeling PJ, Waller RF. Broad genomic and transcriptional analysis reveals a highly derived genome in dinoflagellate mitochondria. BMC Biol. 2007;5:41 10.1186/1741-7007-5-41 17897476 PMC2151934

[13] Nash EA, Barbrook AC, Edwards-Stuart RK, Bernhardt K, Howe CJ, Nisbet RE. Organization of the mitochondrial genome in the dinoflagellate Amphidinium carterae. Mol Biol Evol. 2007;24(7):1528–36. 10.1093/molbev/msm074 17440175

[14] Zhang H, Lin S. mRNA editing and spliced-leader RNA trans-splicing groups Oxyrrhis, Noctiluca, Heterocapsa, and Amphidinium as basal lineages of dinoflagellates. J Phycol. 2008;44(3):703–11. 10.1111/j.1529-8817.2008.00521.x 27041428

[15] Zhang H, Bhattacharya D, Maranda L, Lin S. Mitochondrial cob and cox1 Genes and Editing of the Corresponding mRNAs in Dinophysis acuminata from Narragansett Bay, with Special Reference to the Phylogenetic Position of the Genus Dinophysis. Appl Environ Microbiol. 2008;74(5):1546–54. 10.1128/AEM.02103-07 18165361 PMC2258633

[16] Zauner S, Greilinger D, Laatsch T, Kowallik KV, Maier UG. Substitutional editing of transcripts from genes of cyanobacterial origin in the dinoflagellate Ceratium horridum. FEBS Lett. 2004;577(3):535–8. 10.1016/j.febslet.2004.10.060 15556642

[17] Mungpakdee S, Shinzato C, Takeuchi T, Kawashima T, Koyanagi R, Hisata K, et al Massive gene transfer and extensive RNA editing of a symbiotic dinoflagellate plastid genome. Genome Biol Evol. 2014;6(6):1408–22. 10.1093/gbe/evu109 24881086 PMC4079212

[18] Wang Y, Morse D. Rampant polyuridylylation of plastid gene transcripts in the dinoflagellate Lingulodinium. Nucleic Acids Res. 2006;34(2):613–9. 10.1093/nar/gkj438 16434702 PMC1351369

[19] Dang Y, Green BR. Substitutional editing of Heterocapsa triquetra chloroplast transcripts and a folding model for its divergent chloroplast 16S rRNA. Gene. 2009;442(1–2):73–80. 10.1016/j.gene.2009.04.006 19376212

[20] Jackson CJ, Gornik SG, Waller RF. A tertiary plastid gains RNA editing in its new host. Mol Biol Evol. 2013;30(4):788–92. 10.1093/molbev/mss270 23197592

[21] Santos SR, Coffroth MA. Molecular genetic evidence that dinoflagellates belonging to the genus Symbiodinium freudenthal are haploid. Biol Bull. 2003;204(1):10–20. 10.2307/1543491 12588740

[22] St Laurent G, Tackett MR, Nechkin S, Shtokalo D, Antonets D, Savva YA, et al Genome-wide analysis of A-to-I RNA editing by single-molecule sequencing in Drosophila. Nat Struct Mol Biol. 2013;20(11):1333–9. 10.1038/nsmb.2675 24077224

[23] Daniel C, Lagergren J, Ohman M. RNA editing of non-coding RNA and its role in gene regulation. Biochimie. 2015;117:22–7. 10.1016/j.biochi.2015.05.020 26051678

[24] Bass BL. RNA editing by adenosine deaminases that act on RNA. Annu Rev Biochem. 2002;71:817–46. 10.1146/annurev.biochem.71.110601.135501 12045112 PMC1823043

[25] Smith HC. Measuring editing activity and identifying cytidine-to-uridine mRNA editing factors in cells and biochemical isolates. Methods Enzymol. 2007;424:389–416. 10.1016/S0076-6879(07)24018-2 17662851

[26] Freyer R, Kiefer-Meyer MC, Kossel H. Occurrence of plastid RNA editing in all major lineages of land plants. Proc Natl Acad Sci U S A. 1997;94(12):6285–90. 9177209 10.1073/pnas.94.12.6285 PMC21041

[27] Peng Z, Cheng Y, Tan BC, Kang L, Tian Z, Zhu Y, et al Comprehensive analysis of RNA-Seq data reveals extensive RNA editing in a human transcriptome. Nat Biotechnol. 2012;30(3):253–60. 10.1038/nbt.2122 22327324

[28] Porath HT, Carmi S, Levanon EY. A genome-wide map of hyper-edited RNA reveals numerous new sites. Nat Commun. 2014;5:4726 10.1038/ncomms5726 25158696 PMC4365171

[29] Bailey TL, Boden M, Buske FA, Frith M, Grant CE, Clementi L, et al MEME SUITE: tools for motif discovery and searching. Nucleic Acids Res. 2009;37(Web Server issue):W202–8. 10.1093/nar/gkp335 19458158 PMC2703892

[30] Brehme N, Bayer-Csaszar E, Glass F, Takenaka M. The DYW Subgroup PPR Protein MEF35 Targets RNA Editing Sites in the Mitochondrial rpl16, nad4 and cob mRNAs in Arabidopsis thaliana. PLoS One. 2015;10(10):e0140680 10.1371/journal.pone.0140680 26470017 PMC4607164

[31] Takenaka M, Zehrmann A, Verbitskiy D, Hartel B, Brennicke A. RNA editing in plants and its evolution. Annu Rev Genet. 2013;47:335–52. 10.1146/annurev-genet-111212-133519 24274753

[32] Nishikura K. Functions and regulation of RNA editing by ADAR deaminases. Annu Rev Biochem. 2010;79:321–49. 10.1146/annurev-biochem-060208-105251 20192758 PMC2953425

[33] Aranda M, Li Y, Liew YJ, Baumgarten S, Simakov O, Wilson MC, et al Genomes of coral dinoflagellate symbionts highlight evolutionary adaptations conducive to a symbiotic lifestyle. Sci Rep. 2016;6:39734 10.1038/srep39734 28004835 PMC5177918

[34] Shoguchi E, Shinzato C, Kawashima T, Gyoja F, Mungpakdee S, Koyanagi R, et al Draft assembly of the Symbiodinium minutum nuclear genome reveals dinoflagellate gene structure. Curr Biol. 2013;23(15):1399–408. 10.1016/j.cub.2013.05.062 23850284

[35] Wang IX, So E, Devlin JL, Zhao Y, Wu M, Cheung VG. ADAR regulates RNA editing, transcript stability, and gene expression. Cell Rep. 2013;5(3):849–60. 10.1016/j.celrep.2013.10.002 24183664 PMC3935819

[36] Lin S, Zhang H, Gray MW. RNA editing in dinoflagellates and its implications for the evolutionary history of the editing machinery 2008. 208-306

[37] Roberts SA, Lawrence MS, Klimczak LJ, Grimm SA, Fargo D, Stojanov P, et al An APOBEC Cytidine Deaminase Mutagenesis Pattern is Widespread in Human Cancers. Nat Genet. 2013;45(9):970–6. 10.1038/ng.2702 23852170 PMC3789062

[38] Taylor BJ, Nik-Zainal S, Wu YL, Stebbings LA, Raine K, Campbell PJ, et al DNA deaminases induce break-associated mutation showers with implication of APOBEC3B and 3A in breast cancer kataegis. Elife. 2013;2:e00534 10.7554/eLife.00534 23599896 PMC3628087

[39] Blanc V, Park E, Schaefer S, Miller M, Lin Y, Kennedy S, et al Genome-wide identification and functional analysis of Apobec-1-mediated C-to-U RNA editing in mouse small intestine and liver. Genome Biol. 2014;15(6):R79 10.1186/gb-2014-15-6-r79 24946870 PMC4197816

[40] Giegé P, Brennicke A. RNA editing in Arabidopsis mitochondria effects 441 C to U changes in ORFs. Proc Natl Acad Sci U S A. 1999;96(26):15324–9. 10611383 10.1073/pnas.96.26.15324 PMC24818

[41] Escalante AA, Ayala FJ. Evolutionary origin of Plasmodium and other Apicomplexa based on rRNA genes. Proc Natl Acad Sci U S A. 1995;92(13):5793–7. 7597031 10.1073/pnas.92.13.5793 PMC41587

[42] Alon S, Garrett SC, Levanon EY, Olson S, Graveley BR, Rosenthal JJ, et al The majority of transcripts in the squid nervous system are extensively recoded by A-to-I RNA editing. Elife. 2015;4. 10.7554/eLife.05198 PMC4384741 25569156

[43] Gommans WM, Mullen SP, Maas S. RNA editing: a driving force for adaptive evolution? Bioessays. 2009;31(10):1137–45. 10.1002/bies.200900045 19708020 PMC2829293

[44] Baumgarten S, Bayer T, Aranda M, Liew YJ, Carr A, Micklem G, et al Integrating microRNA and mRNA expression profiling in Symbiodinium microadriaticum, a dinoflagellate symbiont of reef-building corals. BMC Genomics. 2013;14:704 10.1186/1471-2164-14-704 24119094 PMC3853145

[45] Barshis DJ, Ladner JT, Oliver TA, Palumbi SR. Lineage-specific transcriptional profiles of Symbiodinium spp. unaltered by heat stress in a coral host. Mol Biol Evol. 2014;31(6):1343–52. 10.1093/molbev/msu107 24651035

[46] Moustafa A, Evans AN, Kulis DM, Hackett JD, Erdner DL, Anderson DM, et al Transcriptome profiling of a toxic dinoflagellate reveals a gene-rich protist and a potential impact on gene expression due to bacterial presence. PLoS One. 2010;5(3):e9688 10.1371/journal.pone.0009688 20300646 PMC2837391

[47] Bayer T, Aranda M, Sunagawa S, Yum LK, DeSalvo MK, Lindquist E, et al Symbiodinium Transcriptomes: Genome Insights into the Dinoflagellate Symbionts of Reef-Building Corals. PLoS One. 2012;7(4):e35269 10.1371/journal.pone.0035269 22529998 PMC3329448

[48] Parkinson JE, Baumgarten S, Michell CT, Baums IB, LaJeunesse TC, Voolstra CR. Gene expression variation resolves species and individual strains among coral-associated dinoflagellates within the genus Symbiodinium. Genome Biol Evol. 2016 2 11;8(3):665–80. 10.1093/gbe/evw019 . 26868597 PMC4824173

[49] Lin S, Cheng S, Song B, Zhong X, Lin X, Li W, et al The Symbiodinium kawagutii genome illuminates dinoflagellate gene expression and coral symbiosis. Science. 2015;350(6261):691–4. 10.1126/science.aad0408 26542574

[50] Shabalina SA, Spiridonov NA, Kashina A. Sounds of silence: synonymous nucleotides as a key to biological regulation and complexity. Nucleic Acids Res. 2013;41(4):2073–94. 10.1093/nar/gks1205 23293005 PMC3575835

[51] Bolger AM, Lohse M, Usadel B. Trimmomatic: a flexible trimmer for Illumina sequence data. Bioinformatics. 2014;30(15):2114–20. 10.1093/bioinformatics/btu170 24695404 PMC4103590

[52] Langmead B, Salzberg SL. Fast gapped-read alignment with Bowtie 2. Nat Methods. 2012;9(4):357–9. 10.1038/nmeth.1923 22388286 PMC3322381

[53] Picardi E, Pesole G. REDItools: high-throughput RNA editing detection made easy. Bioinformatics. 2013;29(14):1813–4. 10.1093/bioinformatics/btt287 23742983

[54] Kleinman CL, Majewski J. Comment on "Widespread RNA and DNA sequence differences in the human transcriptome". Science. 2012;335(6074):1302 10.1126/science.1209658 22422962

[55] Lin W, Piskol R, Tan MH, Li JB. Comment on "Widespread RNA and DNA sequence differences in the human transcriptome". Science. 2012;335(6074):1302 10.1126/science.1210624 22422962

[56] Pickrell JK, Gilad Y, Pritchard JK. Comment on "Widespread RNA and DNA sequence differences in the human transcriptome". Science. 2012;335(6074):1302 10.1126/science.1210484 PMC5207799 22422963

[57] Kleinman CL, Adoue V, Majewski J. RNA editing of protein sequences: a rare event in human transcriptomes. Rna. 2012;18(9):1586–96. 10.1261/rna.033233.112 22832026 PMC3425774

[58] Park E, Williams B, Wold BJ, Mortazavi A. RNA editing in the human ENCODE RNA-seq data. Genome Res. 2012;22(9):1626–33. 10.1101/gr.134957.111 22955975 PMC3431480

[59] Benjamini Y, Hochberg Y. Controlling the false discovery rate: a practical and powerful approach to multiple testing. J R Stat Soc Series B Stat Methodol. 1995:289–300.

[60] Benjamini Y, Yekutieli D. The control of the false discovery rate in multiple testing under dependency. Ann Stat. 2001:1165–88.

[61] Mendez GS, Delwiche CF, Apt KE, Lippmeier JC. Dinoflagellate Gene Structure and Intron Splice Sites in a Genomic Tandem Array. J Eukaryot Microbiol. 2015;62(5):679–87. 10.1111/jeu.12230 25963315 PMC5032977

[62] Jaeckisch N, Yang I, Wohlrab S, Glockner G, Kroymann J, Vogel H, et al Comparative genomic and transcriptomic characterization of the toxigenic marine dinoflagellate Alexandrium ostenfeldii. PLoS One. 2011;6(12):e28012 10.1371/journal.pone.0028012 22164224 PMC3229502

[63] Beauchemin M, Roy S, Daoust P, Dagenais-Bellefeuille S, Bertomeu T, Letourneau L, et al Dinoflagellate tandem array gene transcripts are highly conserved and not polycistronic. Proc Natl Acad Sci U S A. 2012;109(39):15793–8. 10.1073/pnas.1206683109 23019363 PMC3465430

[64] Barraud P, Allain FH. ADAR proteins: double-stranded RNA and Z-DNA binding domains. Curr Top Microbiol Immunol. 2012;353:35–60. 10.1007/82_2011_145 21728134 PMC3226063

[65] Bairoch A, Boeckmann B, Ferro S, Gasteiger E. Swiss-Prot: juggling between evolution and stability. Brief Bioinform. 2004;5(1):39–55. 15153305 10.1093/bib/5.1.39

[66] Haas BJ, Papanicolaou A, Yassour M, Grabherr M, Blood PD, Bowden J, et al De novo transcript sequence reconstruction from RNA-seq using the Trinity platform for reference generation and analysis. Nat Protoc. 2013;8(8):1494–512. 10.1038/nprot.2013.084 23845962 PMC3875132

[67] Waterhouse AM, Procter JB, Martin DM, Clamp M, Barton GJ. Jalview Version 2—a multiple sequence alignment editor and analysis workbench. Bioinformatics. 2009;25(9):1189–91. 10.1093/bioinformatics/btp033 19151095 PMC2672624

[68] Bray NL, Pimentel H, Melsted P, Pachter L. Near-optimal probabilistic RNA-seq quantification. Nat Biotechnol. 2016;34(5):525–7. 10.1038/nbt.3519 27043002

[69] Pimentel HJ, Bray N, Puente S, Melsted P, Pachter L. Differential analysis of RNA-Seq incorporating quantification uncertainty. bioRxiv. 2016. 10.1038/nmeth.4324 28581496

[70] Cingolani P, Platts A, Wang le L, Coon M, Nguyen T, Wang L, et al A program for annotating and predicting the effects of single nucleotide polymorphisms, SnpEff: SNPs in the genome of Drosophila melanogaster strain w1118; iso-2; iso-3. Fly (Austin). 2012;6(2):80–92. 22728672 10.4161/fly.19695 PMC3679285

[71] Alexa A, Rahnenfuhrer J, Lengauer T. Improved scoring of functional groups from gene expression data by decorrelating GO graph structure. Bioinformatics. 2006;22(13):1600–7. 10.1093/bioinformatics/btl140 16606683

[72] Untergasser A, Cutcutache I, Koressaar T, Ye J, Faircloth BC, Remm M, et al Primer3—new capabilities and interfaces. Nucleic Acids Res. 2012;40(15):e115 10.1093/nar/gks596 22730293 PMC3424584

[73] Roy S, Schreiber E. Detecting and Quantifying Low Level Gene Variants in Sanger Sequencing Traces Using the ab1 Peak Reporter Tool. J Biomol Tech. 2014;25(Suppl):S13–S4.

[74] Liew YJ, Aranda M, Voolstra CR. Reefgenomics.Org—a repository for marine genomics data. Database (Oxford). 2016;2016 12 26;2016. pii: baw152. 10.1093/database/baw152 PMC5199144 28025343

[75] Dorrell RG, Howe CJ. Functional remodeling of RNA processing in replacement chloroplasts by pathways retained from their predecessors. Proc Natl Acad Sci U S A. 2012;109(46):18879–84. 23112181 10.1073/pnas.1212270109 PMC3503182

[76] Richardson E, Dorrell RG, Howe CJ. Genome-wide transcript profiling reveals the coevolution of plastid gene sequences and transcript processing pathways in the fucoxanthin dinoflagellate Karlodinium veneficum. Mol Biol Evol 2014; 31(9):2376–86. 10.1093/molbev/msu189 24925926 PMC4137713

