# Supplementary figures and images for: Condition-specific RNA editing in the coral symbiont Symbiodinium microadriaticum (part 1 of 2)
Source: PLoS Genet. 2017 Feb 28;13(2):e1006619. doi: 10.1371/journal.pgen.1006619 (PMC5357065; doi:10.1371/journal.pgen.1006619)

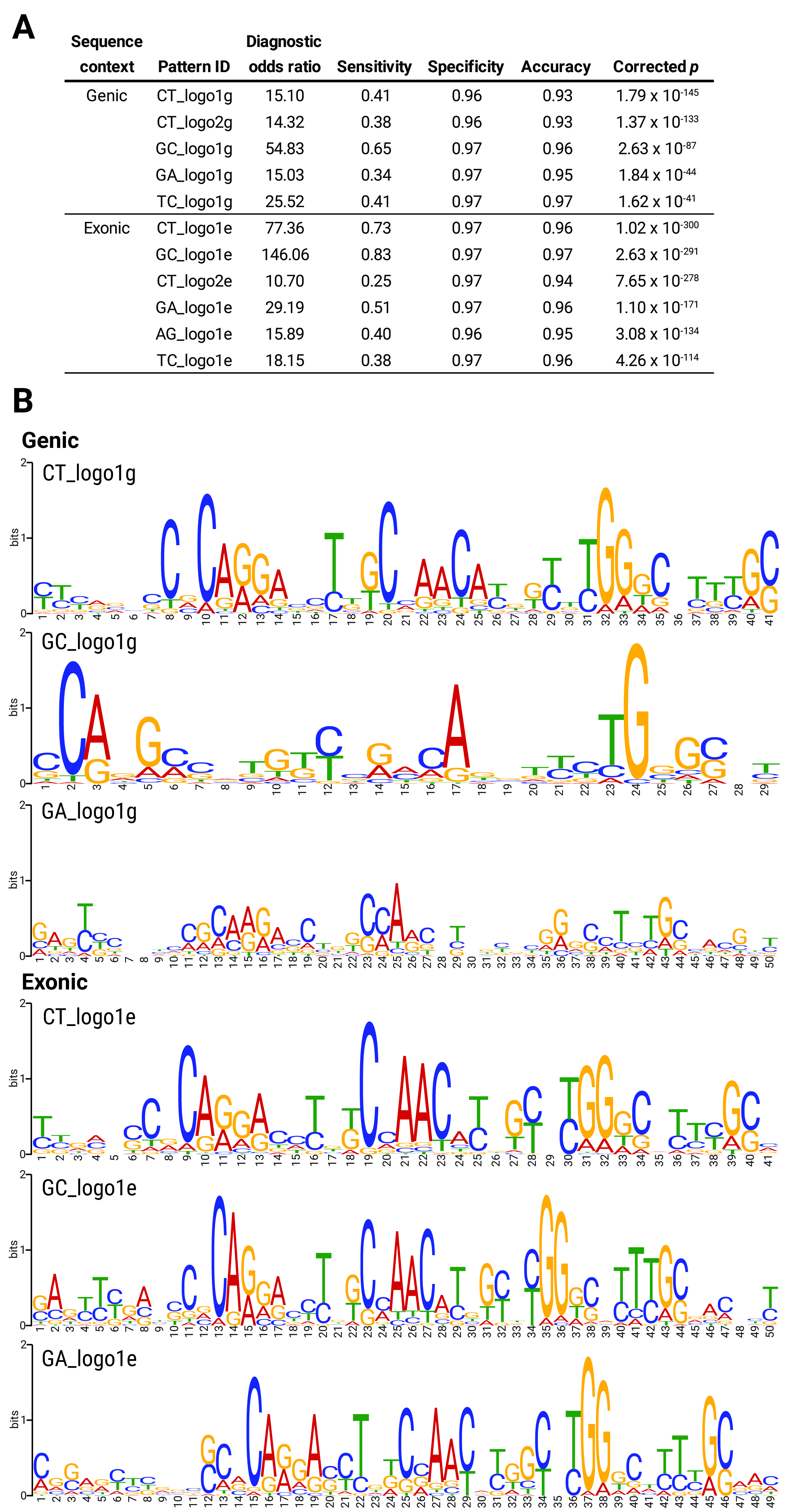

Supplement: S1 Fig — (A) motifs that are highly significant (Benjamini-Hochberg corrected p < 10−40, odds ratio > 10) are listed here. (B) Highest-scoring motifs from C-to-T, G-to-C and G-to-A edits. “Genic” and “exonic” refers to the sequence context used in deriving the motifs. (TIF) [file pgen.1006619.s001.tif]

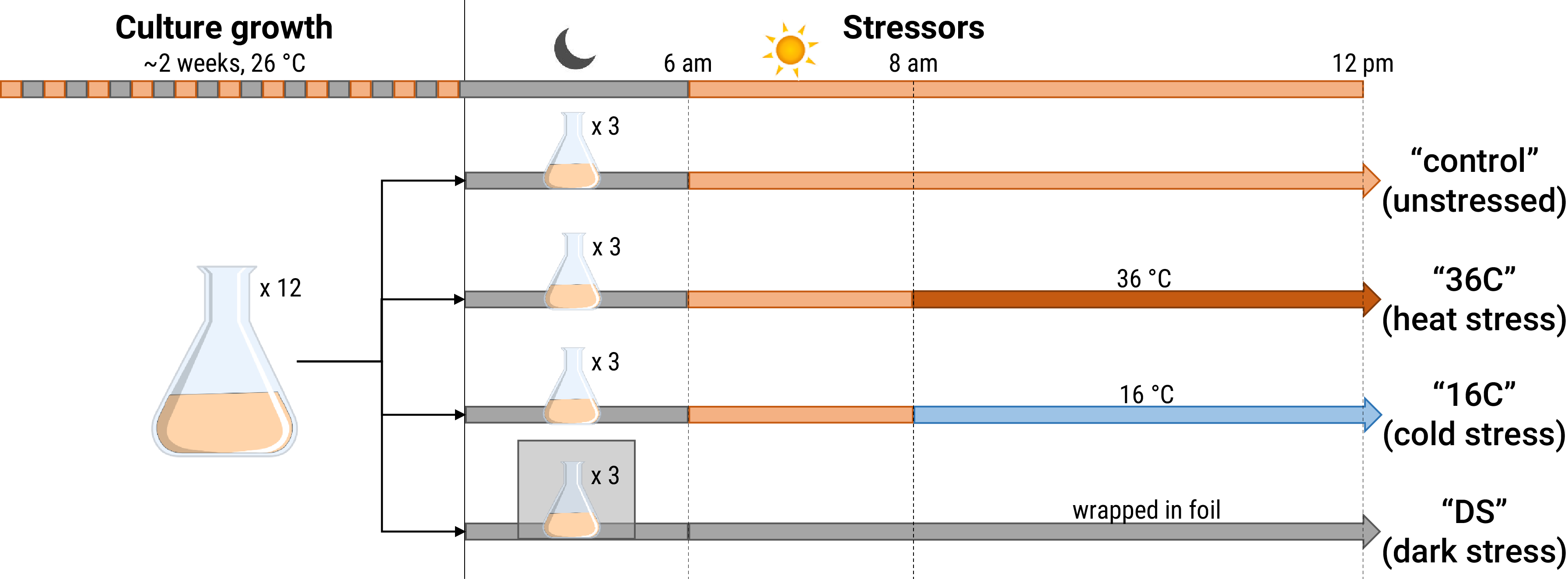

Supplement: S2 Fig — Number of replicates are denoted in the top-right corner of the flasks. Unless noted otherwise, cultures were grown at 26°C. (TIF) [file pgen.1006619.s002.tif]

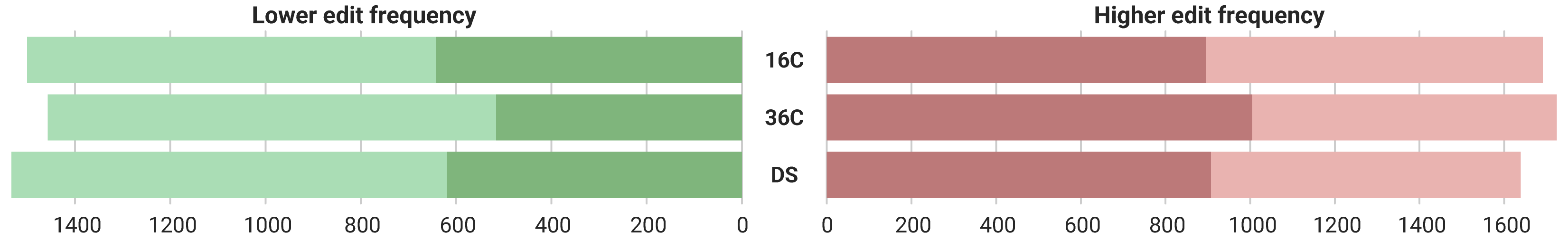

Supplement: S3 Fig — The number of sites (on the x-axis) that underwent increases in edit frequencies are in red; decreases are in green. The trends between all edited sites (pale green/red) is contrasted against the set of 114 differentially edited genes (dark green/red). In contrast to cold stress (“16C”) and dark stress (“DS”), there is a noticeable preference for increased editing in dealing with heat stress (“36C”). (TIF) [file pgen.1006619.s003.tif]

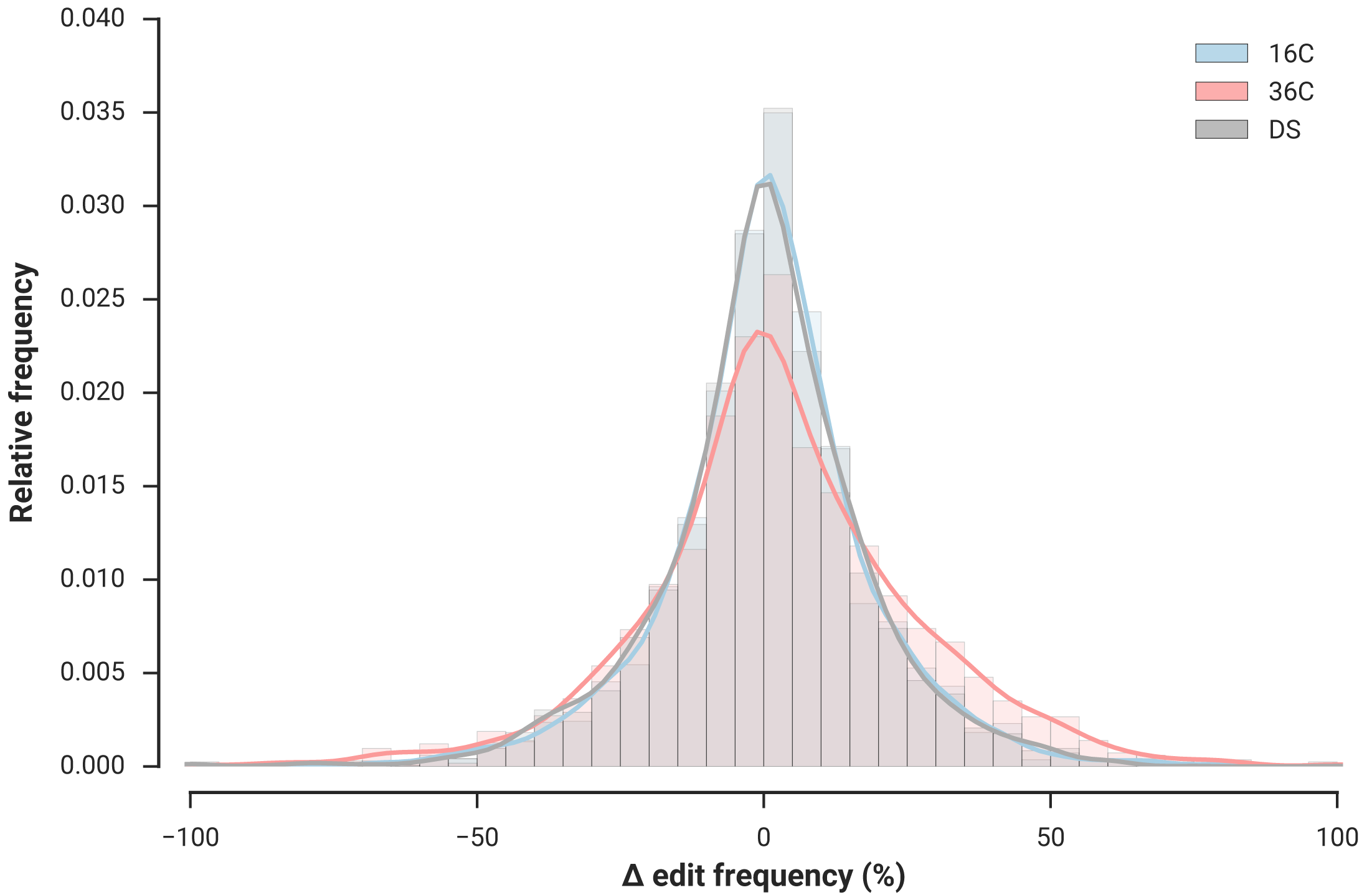

Supplement: S4 Fig — Positive values along the x-axis indicate an increase in edit frequencies under stress; negative values indicate decrease in edit frequencies. (TIF) [file pgen.1006619.s004.tif]

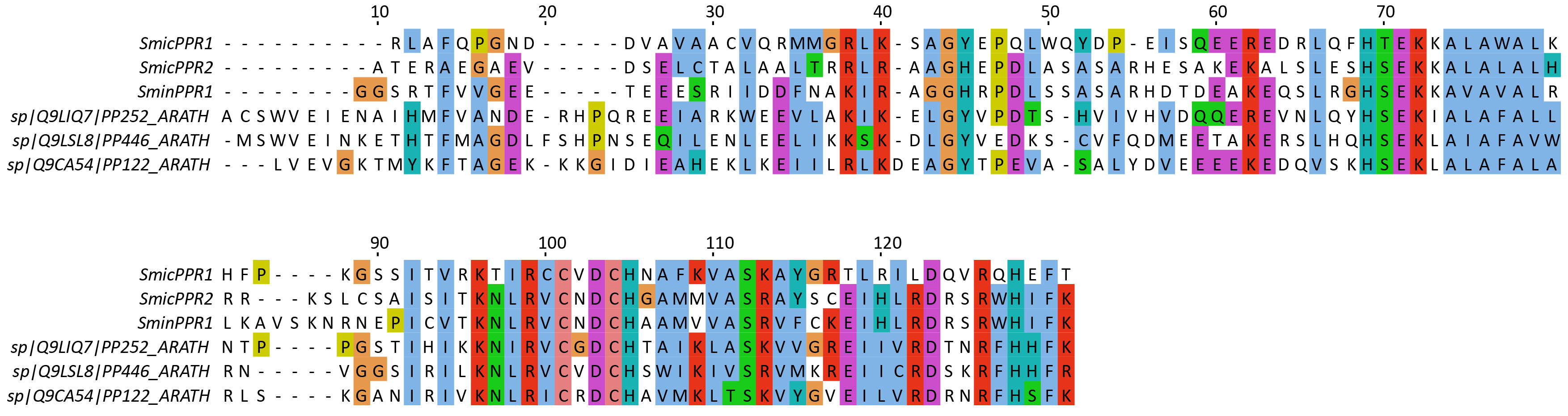

Supplement: S5 Fig — Candidate homologues from S. microadriaticum (“Smic”) and S. minutum (“Smin”) were aligned against known proteins from A. thaliana. Amino acids are coloured according to the Clustal X colour scheme in Jalview. (TIF) [file pgen.1006619.s005.tif]

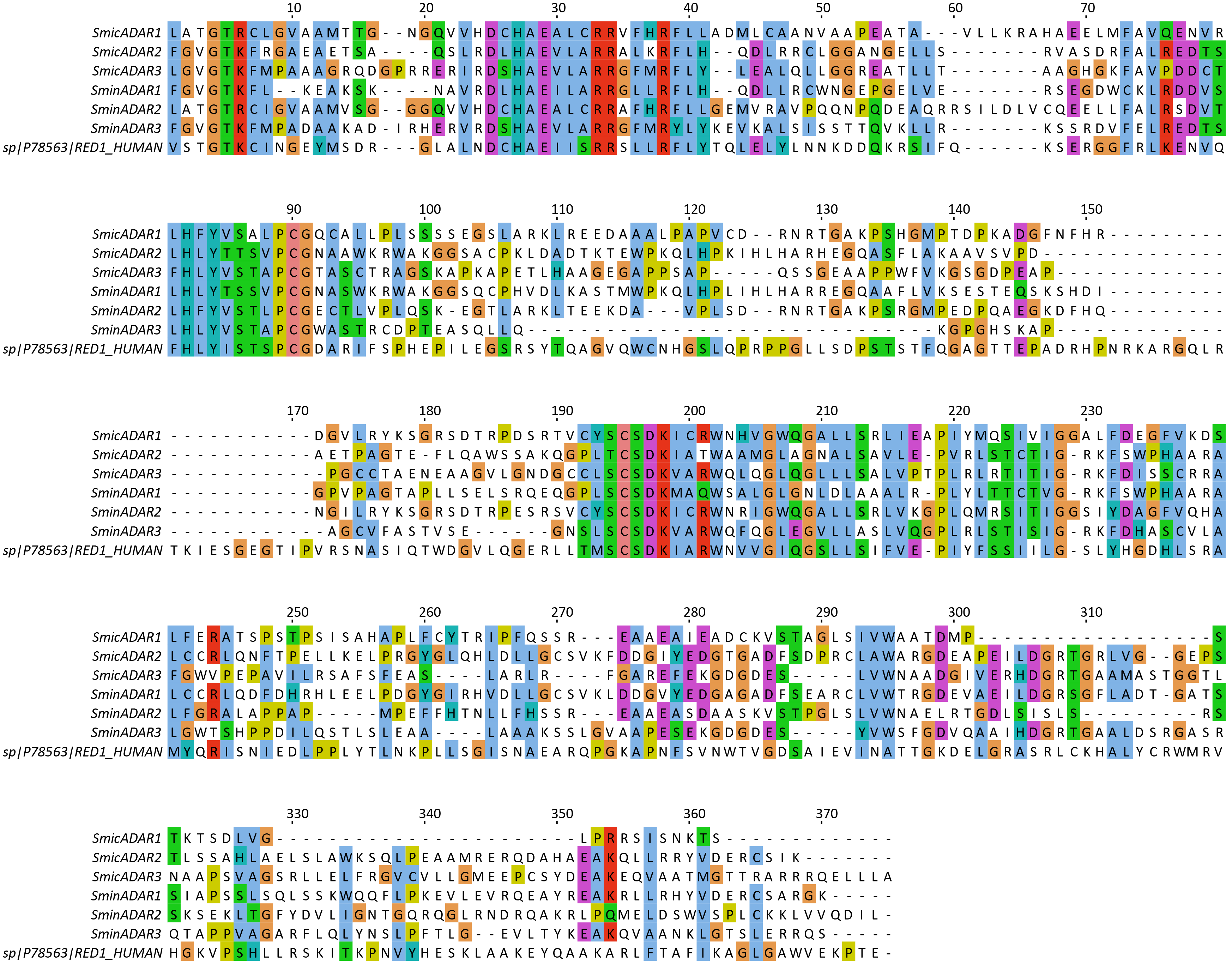

Supplement: S6 Fig — Candidate homologues from S. microadriaticum (“Smic”) and S. minutum (“Smin”) were aligned against known proteins from H. sapiens. Amino acids are coloured according to the Clustal X colour scheme in Jalview. (TIF) [file pgen.1006619.s006.tif]

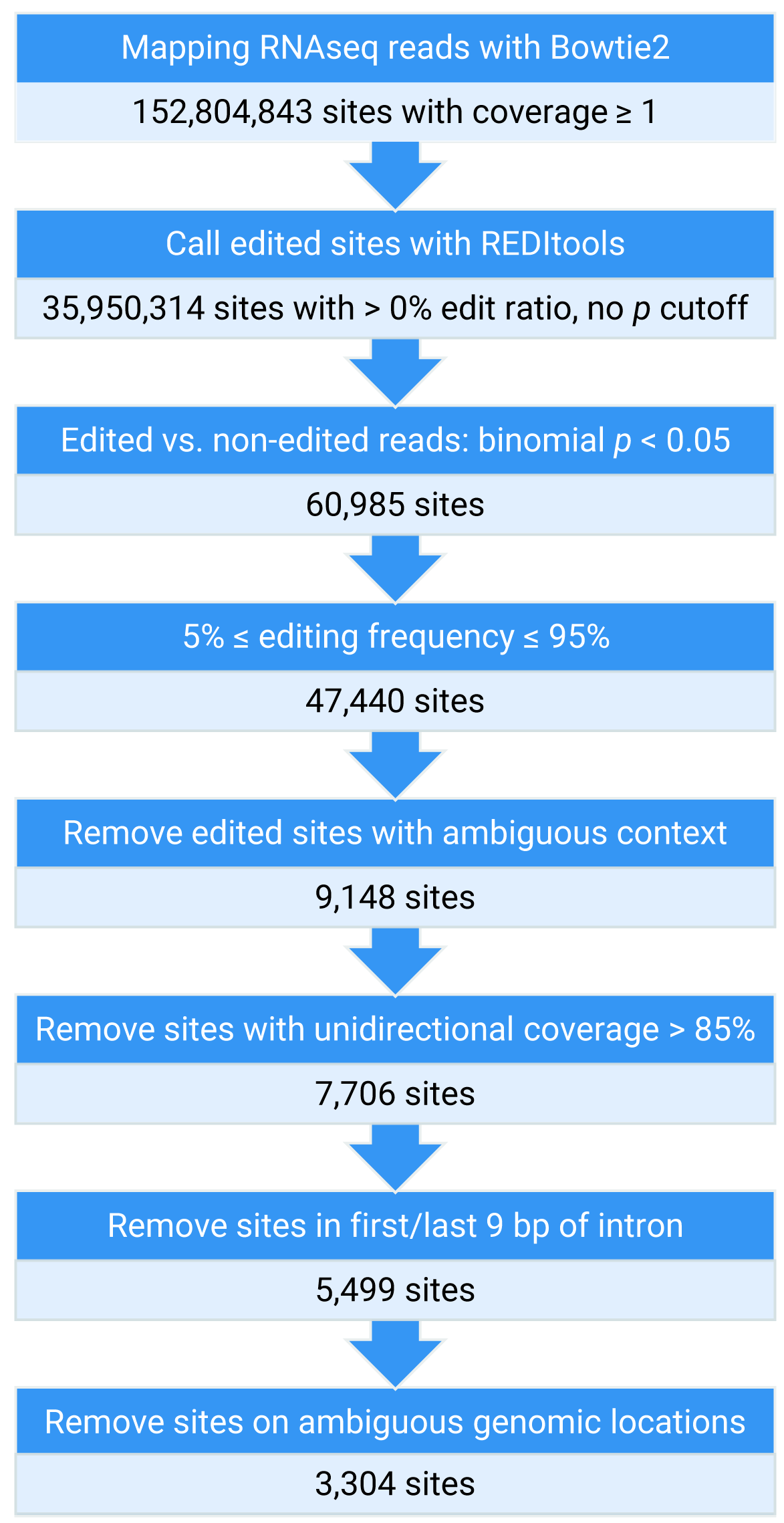

Supplement: S8 Fig — At every stage of the pipeline, the number of sites passing all upstream filters are denoted in the light-blue boxes. (TIF) [file pgen.1006619.s008.tif]

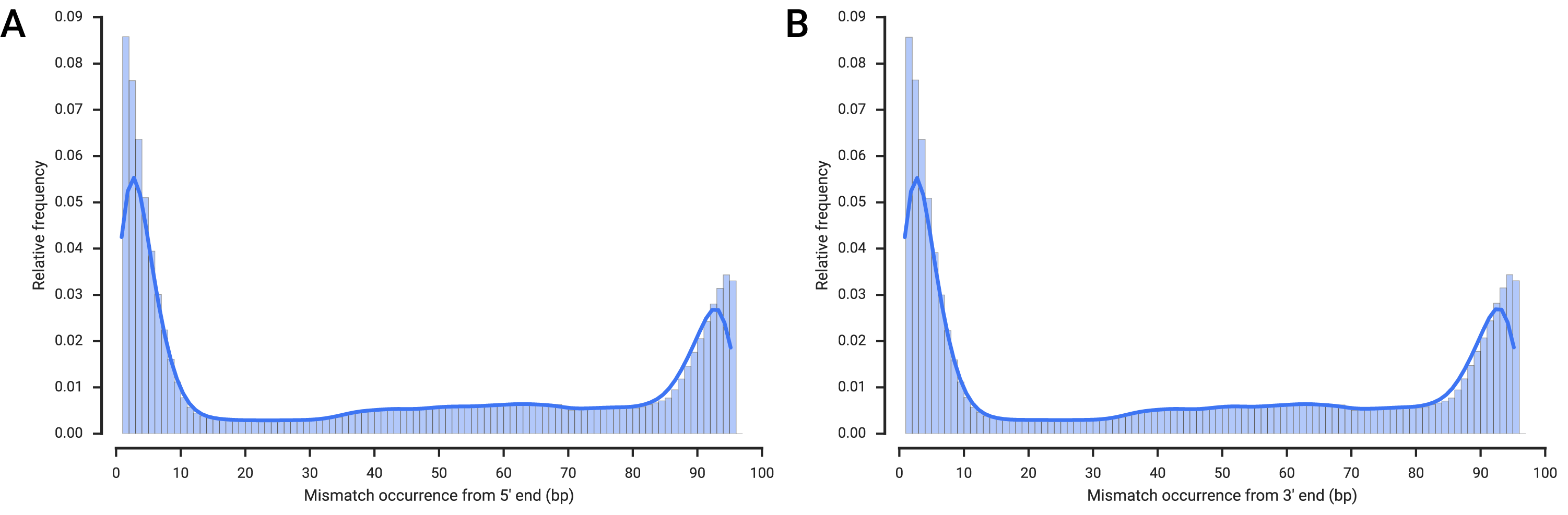

Supplement: S9 Fig — Histograms showing per-position error rates from the (A) 5' end and (B) 3' end respectively. The first 9 bases from both ends have far more mismatches than following bases, which—if included in our analyses—would produce numerous false positives. (TIF) [file pgen.1006619.s009.tif]

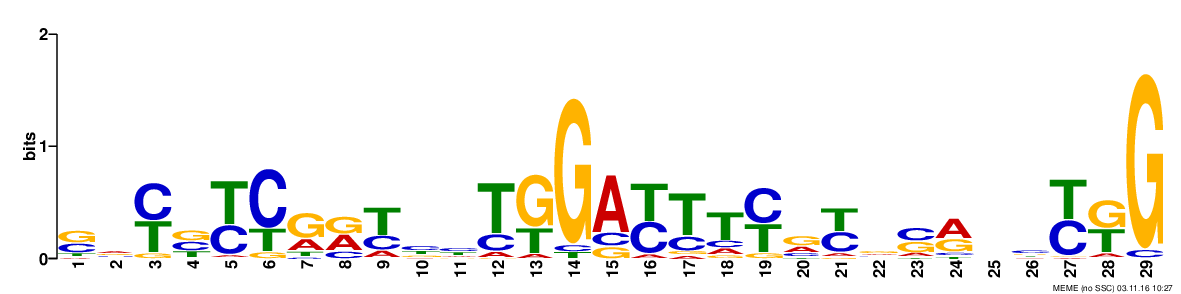

Supplement: S2 Dataset — (GZ) [file pgen.1006619.s020.tar.gz › motif_logo/exonic_edits/CT_meme/logo2.png]

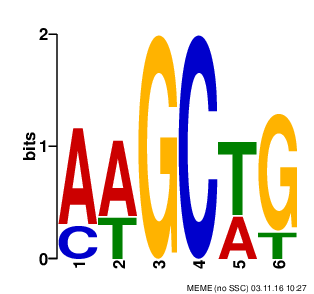

Supplement: S2 Dataset — (GZ) [file pgen.1006619.s020.tar.gz › motif_logo/exonic_edits/CT_meme/logo3.png]

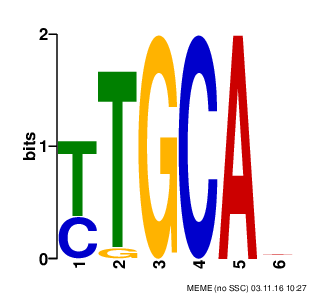

Supplement: S2 Dataset — (GZ) [file pgen.1006619.s020.tar.gz › motif_logo/exonic_edits/CT_meme/logo_rc6.png]

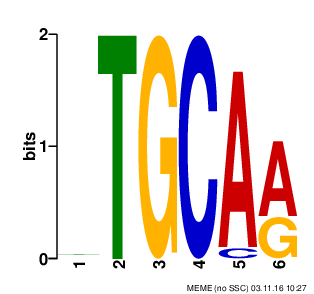

Supplement: S2 Dataset — (GZ) [file pgen.1006619.s020.tar.gz › motif_logo/exonic_edits/CT_meme/logo6.png]

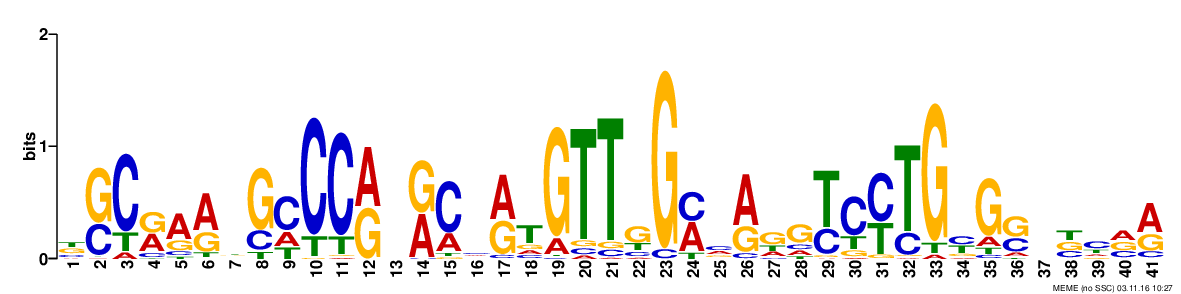

Supplement: S2 Dataset — (GZ) [file pgen.1006619.s020.tar.gz › motif_logo/exonic_edits/CT_meme/logo_rc1.png]

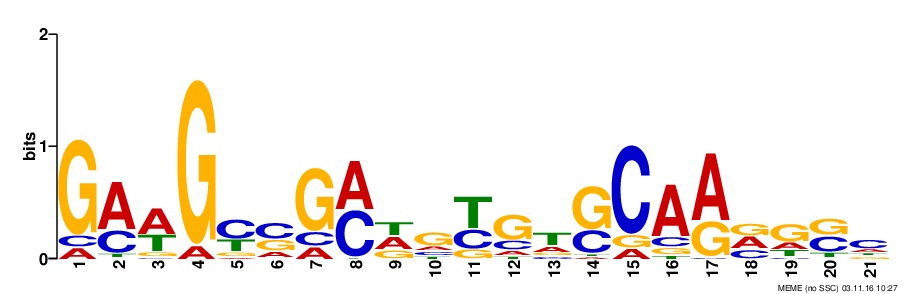

Supplement: S2 Dataset — (GZ) [file pgen.1006619.s020.tar.gz › motif_logo/exonic_edits/CT_meme/logo_rc5.png]

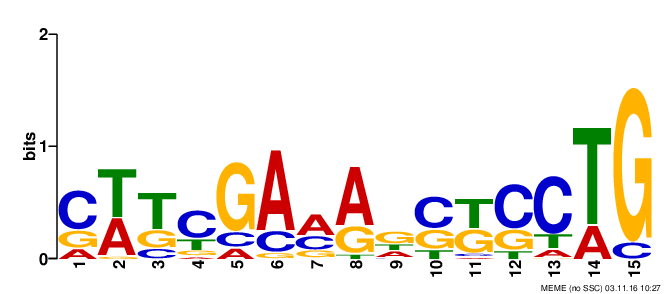

Supplement: S2 Dataset — (GZ) [file pgen.1006619.s020.tar.gz › motif_logo/exonic_edits/CT_meme/logo_rc4.png]

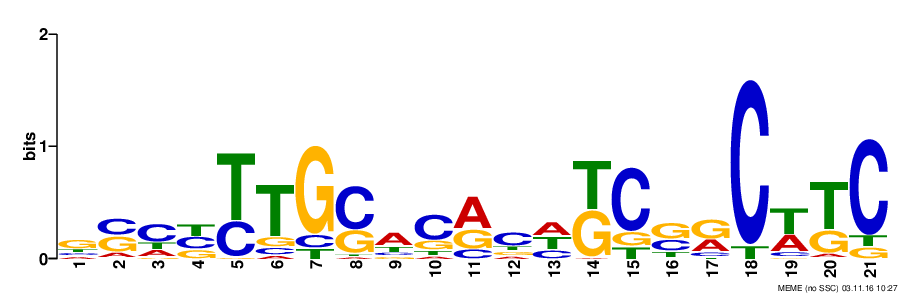

Supplement: S2 Dataset — (GZ) [file pgen.1006619.s020.tar.gz › motif_logo/exonic_edits/CT_meme/logo5.png]

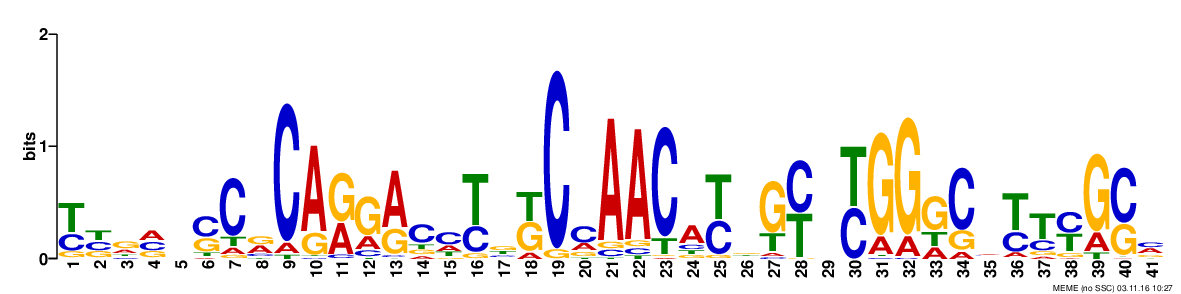

Supplement: S2 Dataset — (GZ) [file pgen.1006619.s020.tar.gz › motif_logo/exonic_edits/CT_meme/logo1.png]

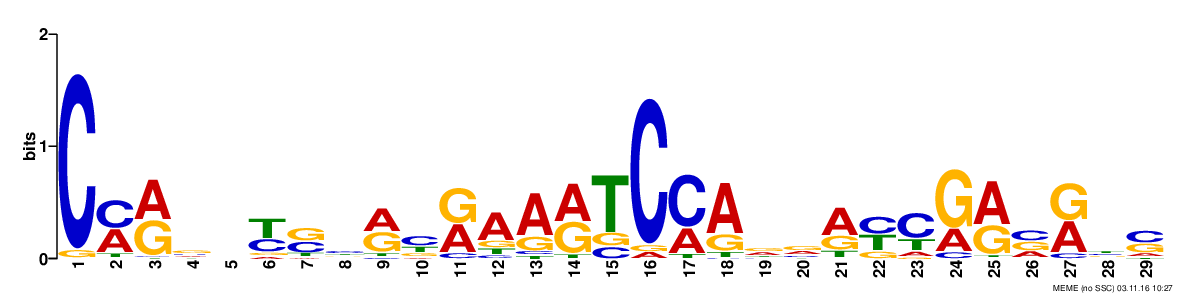

Supplement: S2 Dataset — (GZ) [file pgen.1006619.s020.tar.gz › motif_logo/exonic_edits/CT_meme/logo_rc2.png]

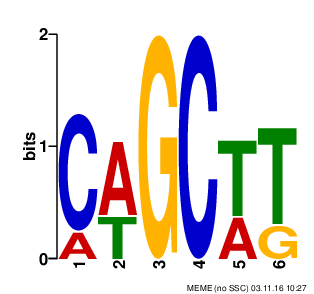

Supplement: S2 Dataset — (GZ) [file pgen.1006619.s020.tar.gz › motif_logo/exonic_edits/CT_meme/logo_rc3.png]

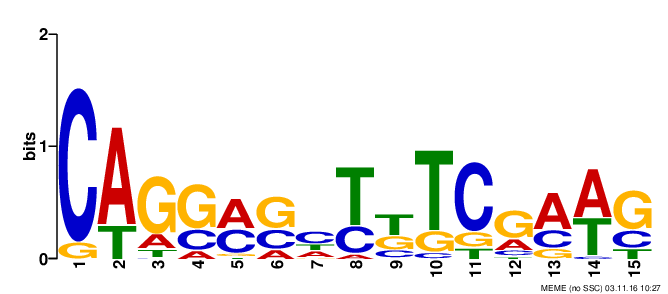

Supplement: S2 Dataset — (GZ) [file pgen.1006619.s020.tar.gz › motif_logo/exonic_edits/CT_meme/logo4.png]

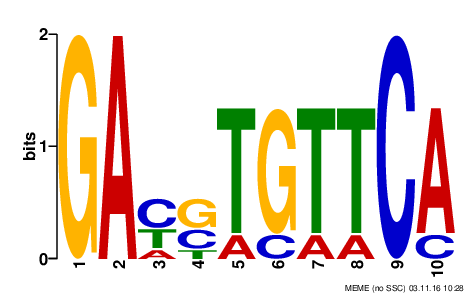

Supplement: S2 Dataset — (GZ) [file pgen.1006619.s020.tar.gz › motif_logo/exonic_edits/TA_meme/logo2.png]

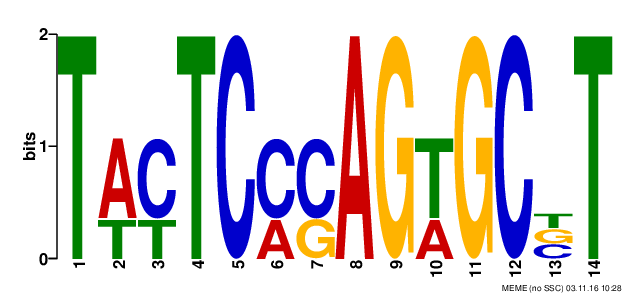

Supplement: S2 Dataset — (GZ) [file pgen.1006619.s020.tar.gz › motif_logo/exonic_edits/TA_meme/logo3.png]

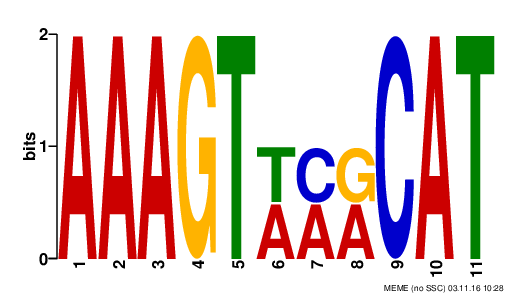

Supplement: S2 Dataset — (GZ) [file pgen.1006619.s020.tar.gz › motif_logo/exonic_edits/TA_meme/logo_rc6.png]

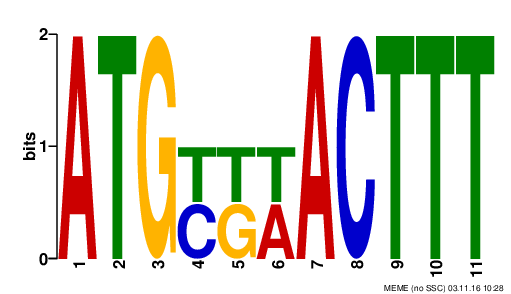

Supplement: S2 Dataset — (GZ) [file pgen.1006619.s020.tar.gz › motif_logo/exonic_edits/TA_meme/logo6.png]

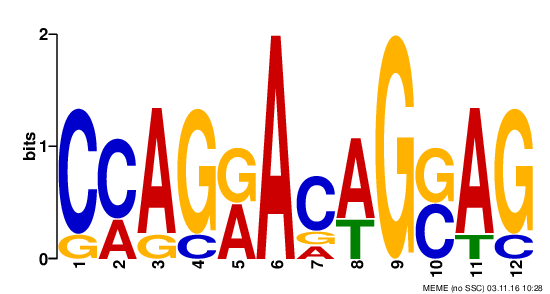

Supplement: S2 Dataset — (GZ) [file pgen.1006619.s020.tar.gz › motif_logo/exonic_edits/TA_meme/logo_rc1.png]

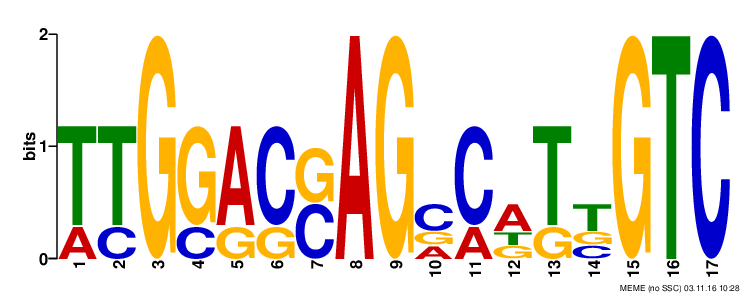

Supplement: S2 Dataset — (GZ) [file pgen.1006619.s020.tar.gz › motif_logo/exonic_edits/TA_meme/logo_rc5.png]

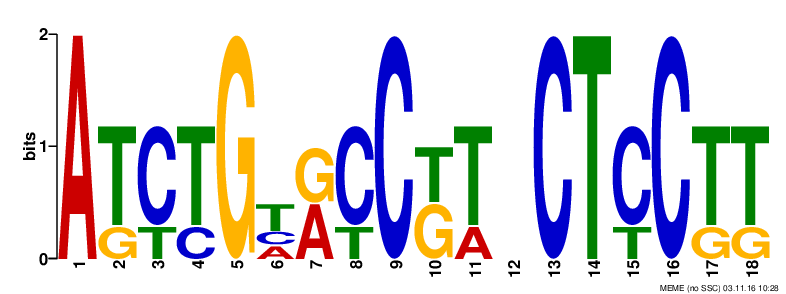

Supplement: S2 Dataset — (GZ) [file pgen.1006619.s020.tar.gz › motif_logo/exonic_edits/TA_meme/logo_rc4.png]

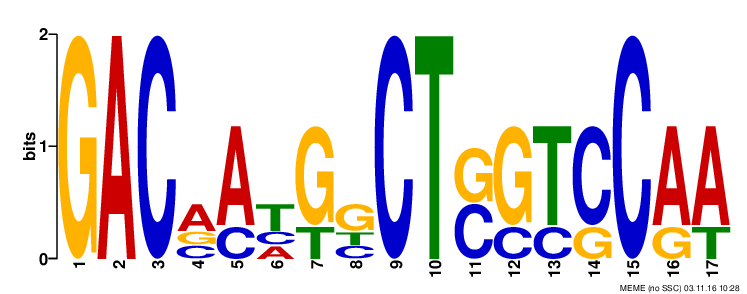

Supplement: S2 Dataset — (GZ) [file pgen.1006619.s020.tar.gz › motif_logo/exonic_edits/TA_meme/logo5.png]

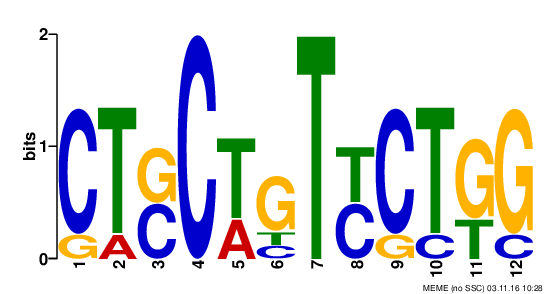

Supplement: S2 Dataset — (GZ) [file pgen.1006619.s020.tar.gz › motif_logo/exonic_edits/TA_meme/logo1.png]

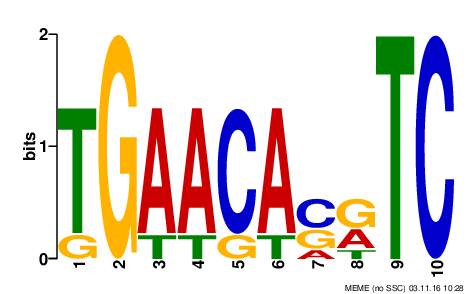

Supplement: S2 Dataset — (GZ) [file pgen.1006619.s020.tar.gz › motif_logo/exonic_edits/TA_meme/logo_rc2.png]

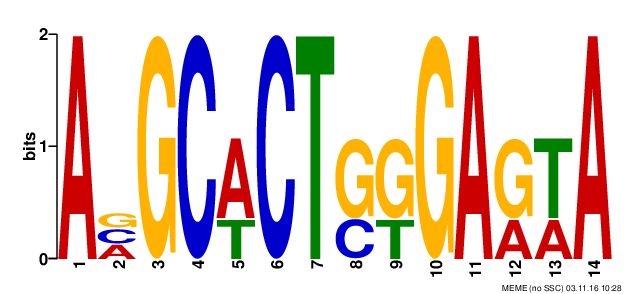

Supplement: S2 Dataset — (GZ) [file pgen.1006619.s020.tar.gz › motif_logo/exonic_edits/TA_meme/logo_rc3.png]

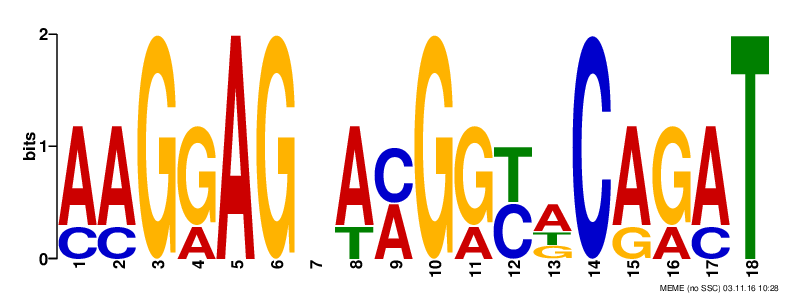

Supplement: S2 Dataset — (GZ) [file pgen.1006619.s020.tar.gz › motif_logo/exonic_edits/TA_meme/logo4.png]

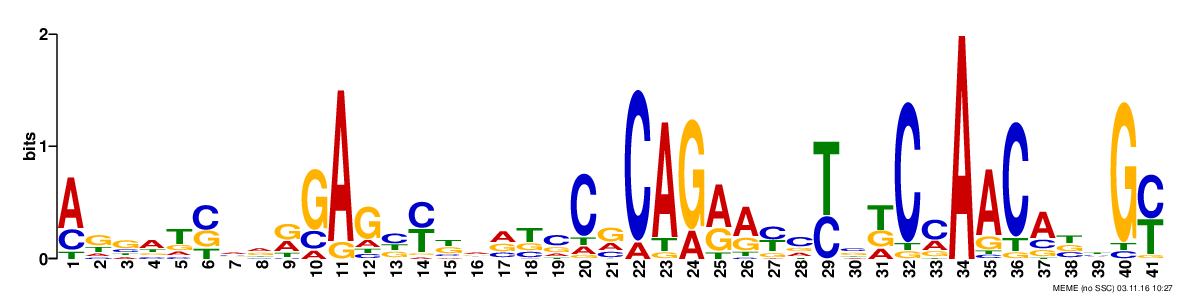

Supplement: S2 Dataset — (GZ) [file pgen.1006619.s020.tar.gz › motif_logo/exonic_edits/GC_meme/logo2.png]

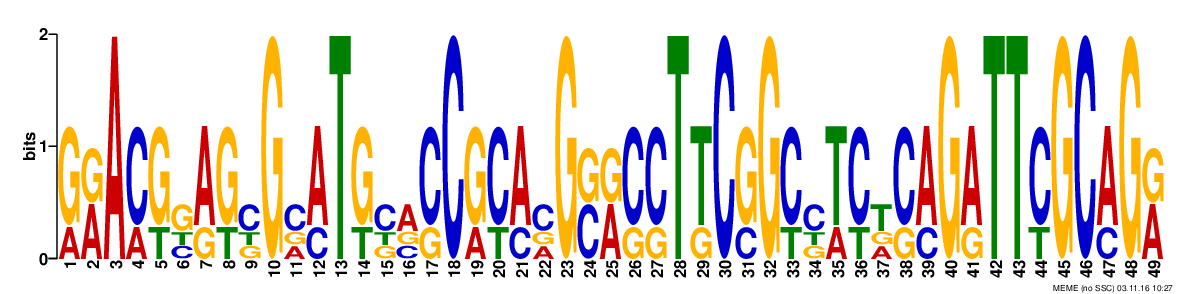

Supplement: S2 Dataset — (GZ) [file pgen.1006619.s020.tar.gz › motif_logo/exonic_edits/GC_meme/logo3.png]

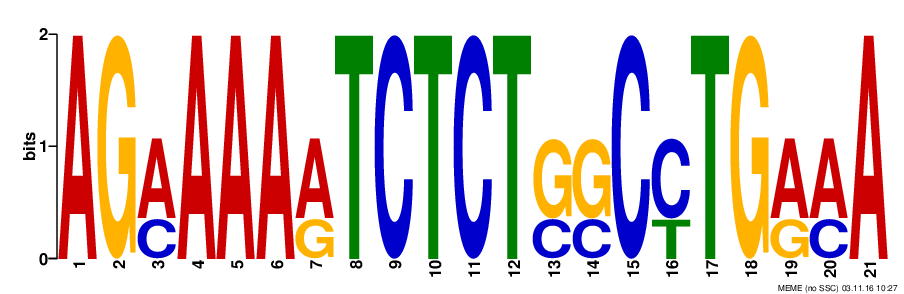

Supplement: S2 Dataset — (GZ) [file pgen.1006619.s020.tar.gz › motif_logo/exonic_edits/GC_meme/logo_rc6.png]

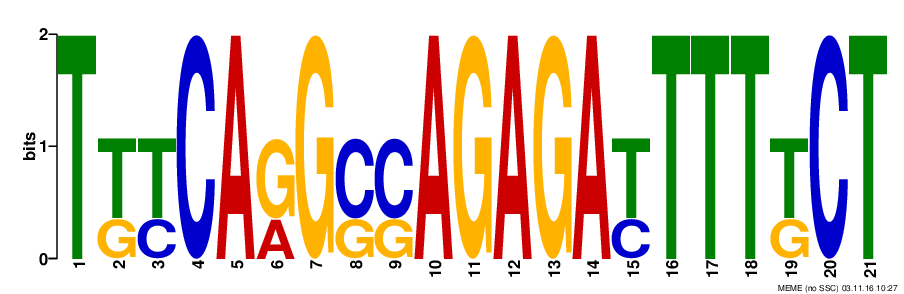

Supplement: S2 Dataset — (GZ) [file pgen.1006619.s020.tar.gz › motif_logo/exonic_edits/GC_meme/logo6.png]

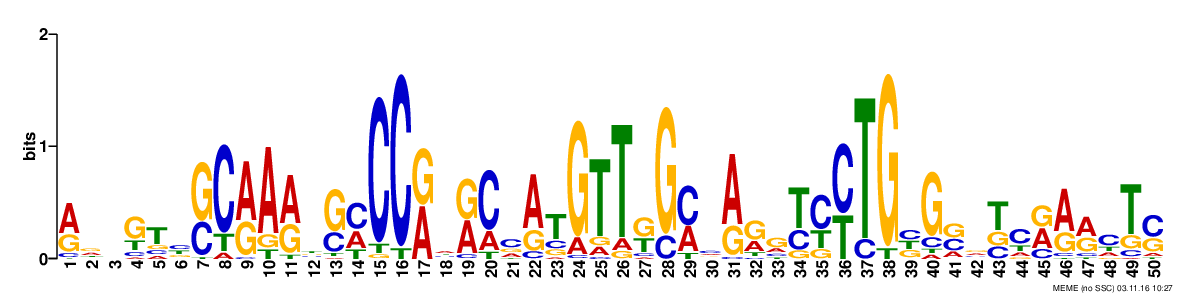

Supplement: S2 Dataset — (GZ) [file pgen.1006619.s020.tar.gz › motif_logo/exonic_edits/GC_meme/logo_rc1.png]

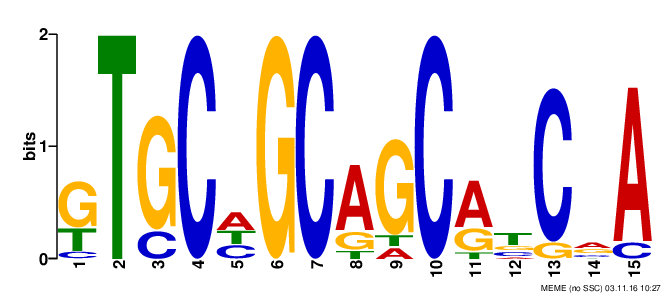

Supplement: S2 Dataset — (GZ) [file pgen.1006619.s020.tar.gz › motif_logo/exonic_edits/GC_meme/logo_rc5.png]

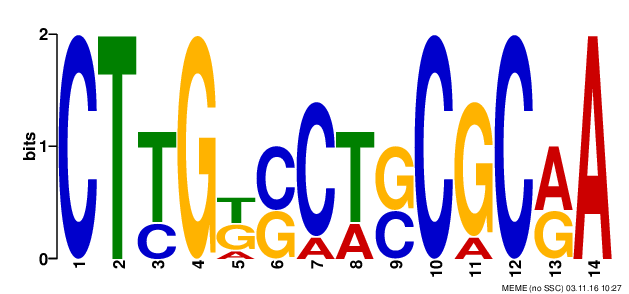

Supplement: S2 Dataset — (GZ) [file pgen.1006619.s020.tar.gz › motif_logo/exonic_edits/GC_meme/logo_rc4.png]

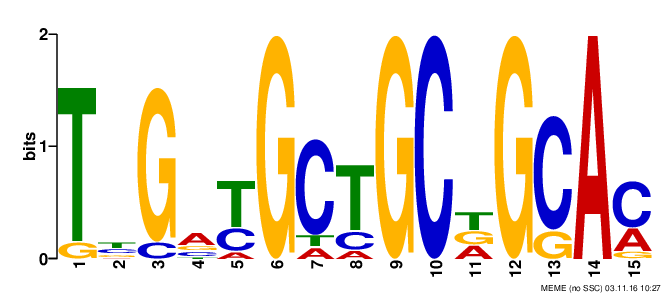

Supplement: S2 Dataset — (GZ) [file pgen.1006619.s020.tar.gz › motif_logo/exonic_edits/GC_meme/logo5.png]

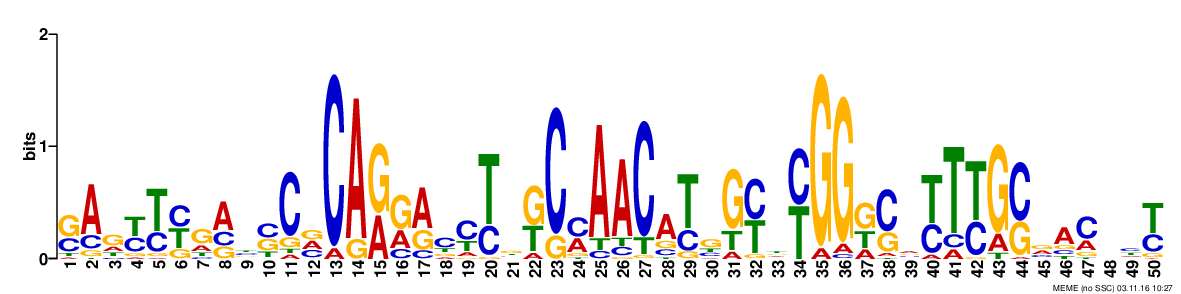

Supplement: S2 Dataset — (GZ) [file pgen.1006619.s020.tar.gz › motif_logo/exonic_edits/GC_meme/logo1.png]

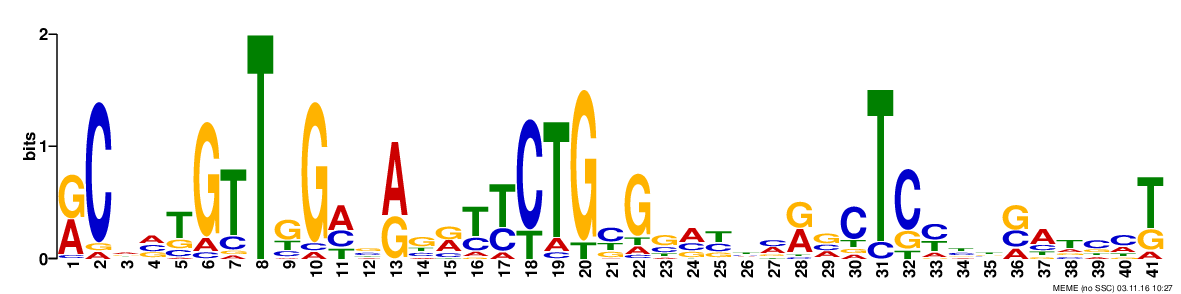

Supplement: S2 Dataset — (GZ) [file pgen.1006619.s020.tar.gz › motif_logo/exonic_edits/GC_meme/logo_rc2.png]

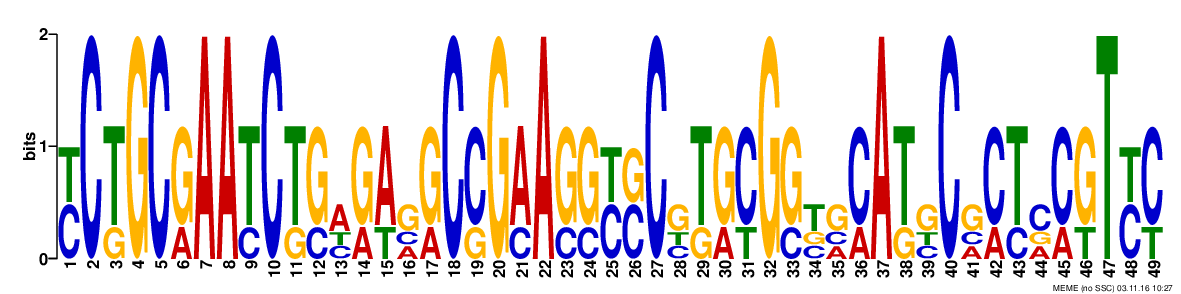

Supplement: S2 Dataset — (GZ) [file pgen.1006619.s020.tar.gz › motif_logo/exonic_edits/GC_meme/logo_rc3.png]

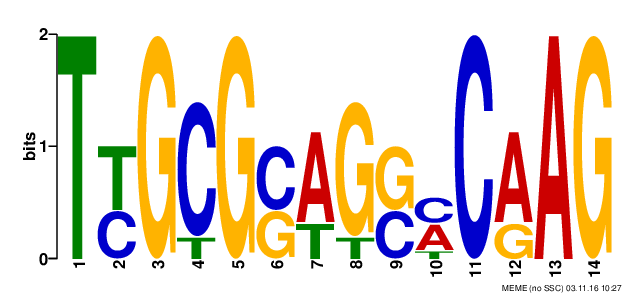

Supplement: S2 Dataset — (GZ) [file pgen.1006619.s020.tar.gz › motif_logo/exonic_edits/GC_meme/logo4.png]

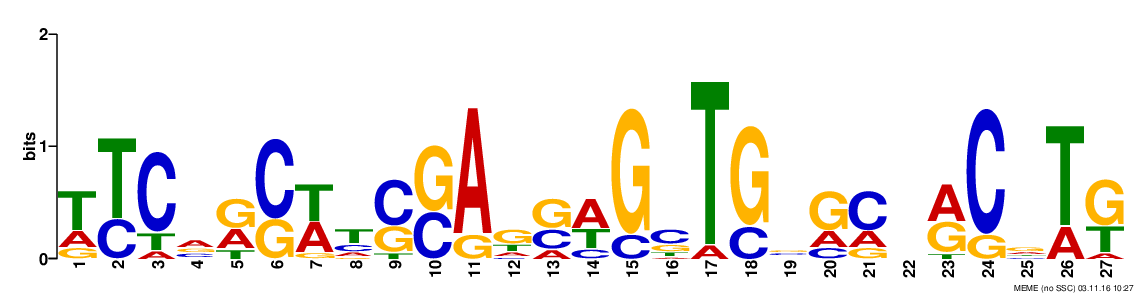

Supplement: S2 Dataset — (GZ) [file pgen.1006619.s020.tar.gz › motif_logo/exonic_edits/CA_meme/logo2.png]

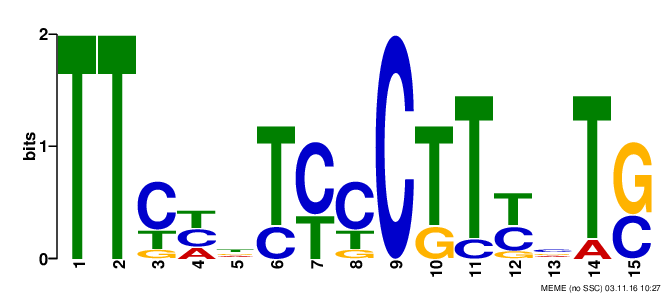

Supplement: S2 Dataset — (GZ) [file pgen.1006619.s020.tar.gz › motif_logo/exonic_edits/CA_meme/logo3.png]

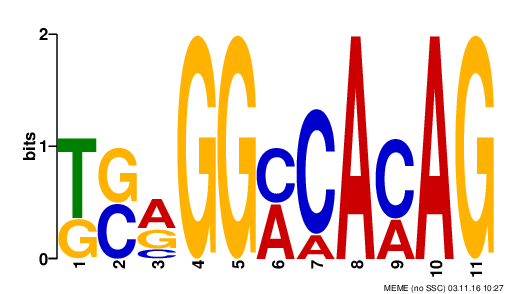

Supplement: S2 Dataset — (GZ) [file pgen.1006619.s020.tar.gz › motif_logo/exonic_edits/CA_meme/logo_rc6.png]

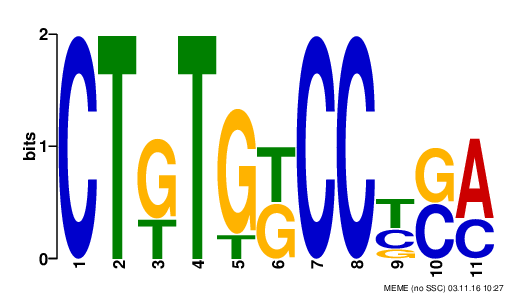

Supplement: S2 Dataset — (GZ) [file pgen.1006619.s020.tar.gz › motif_logo/exonic_edits/CA_meme/logo6.png]

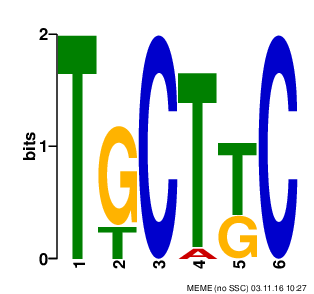

Supplement: S2 Dataset — (GZ) [file pgen.1006619.s020.tar.gz › motif_logo/exonic_edits/CA_meme/logo_rc1.png]

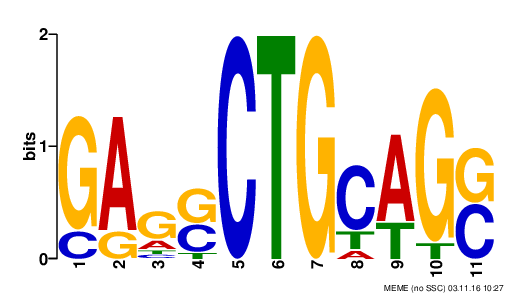

Supplement: S2 Dataset — (GZ) [file pgen.1006619.s020.tar.gz › motif_logo/exonic_edits/CA_meme/logo_rc5.png]

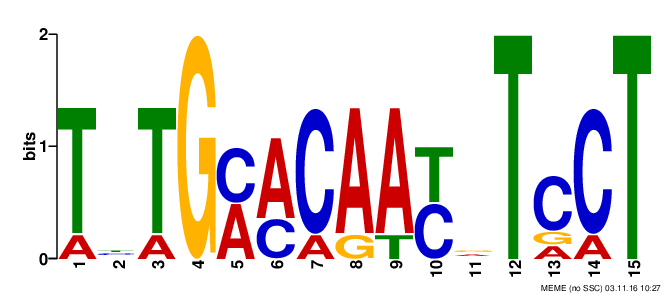

Supplement: S2 Dataset — (GZ) [file pgen.1006619.s020.tar.gz › motif_logo/exonic_edits/CA_meme/logo_rc4.png]

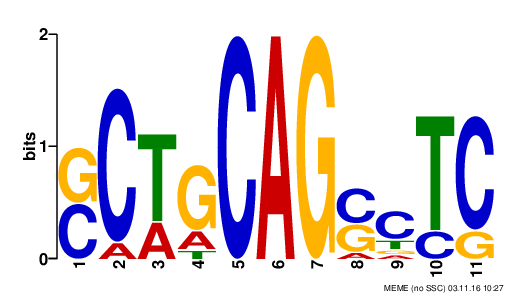

Supplement: S2 Dataset — (GZ) [file pgen.1006619.s020.tar.gz › motif_logo/exonic_edits/CA_meme/logo5.png]

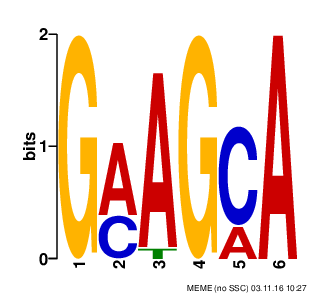

Supplement: S2 Dataset — (GZ) [file pgen.1006619.s020.tar.gz › motif_logo/exonic_edits/CA_meme/logo1.png]

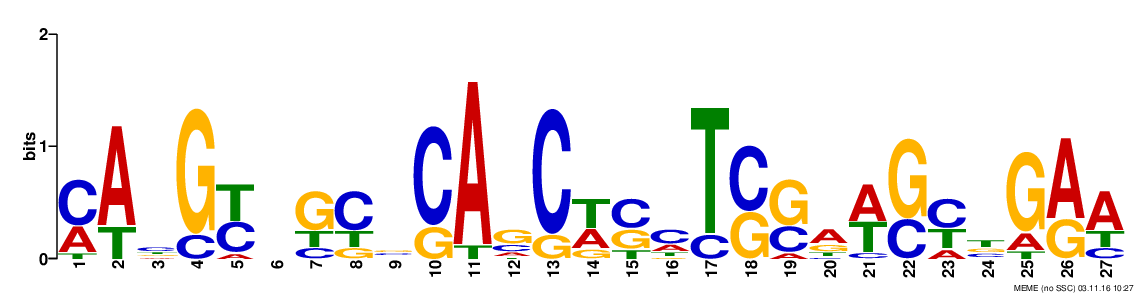

Supplement: S2 Dataset — (GZ) [file pgen.1006619.s020.tar.gz › motif_logo/exonic_edits/CA_meme/logo_rc2.png]

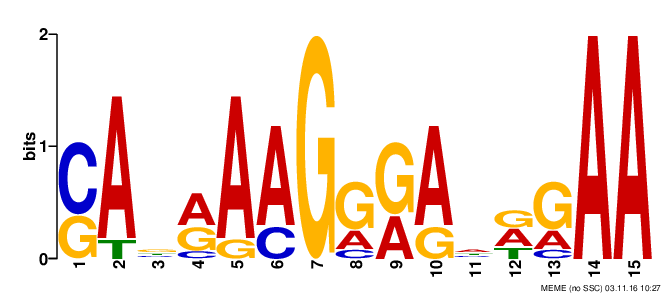

Supplement: S2 Dataset — (GZ) [file pgen.1006619.s020.tar.gz › motif_logo/exonic_edits/CA_meme/logo_rc3.png]

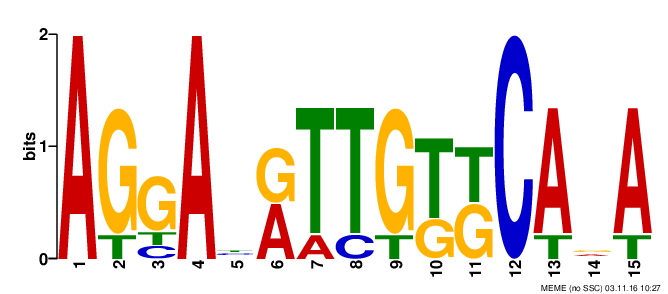

Supplement: S2 Dataset — (GZ) [file pgen.1006619.s020.tar.gz › motif_logo/exonic_edits/CA_meme/logo4.png]

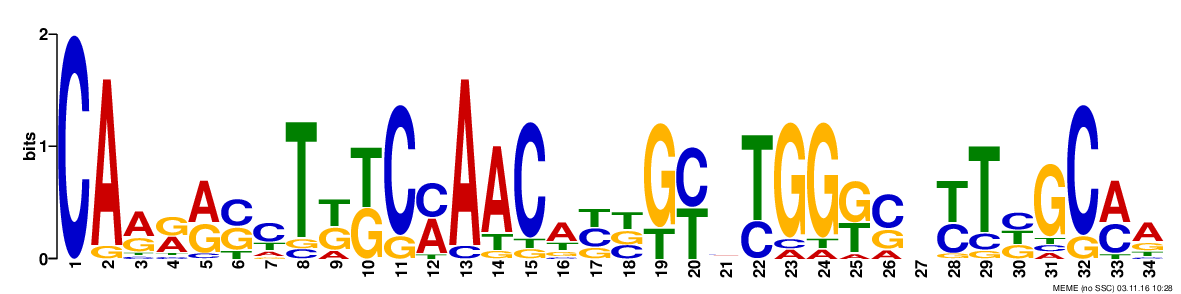

Supplement: S2 Dataset — (GZ) [file pgen.1006619.s020.tar.gz › motif_logo/exonic_edits/TC_meme/logo2.png]

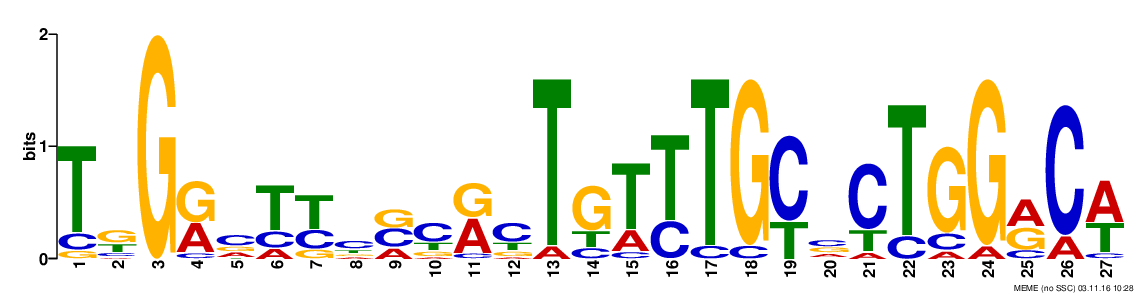

Supplement: S2 Dataset — (GZ) [file pgen.1006619.s020.tar.gz › motif_logo/exonic_edits/TC_meme/logo3.png]

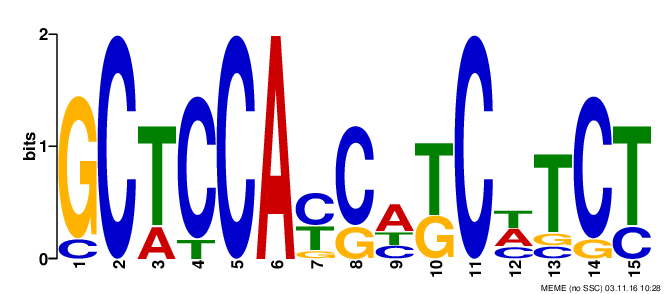

Supplement: S2 Dataset — (GZ) [file pgen.1006619.s020.tar.gz › motif_logo/exonic_edits/TC_meme/logo_rc6.png]

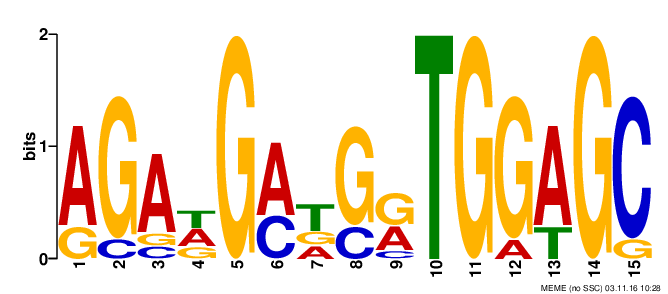

Supplement: S2 Dataset — (GZ) [file pgen.1006619.s020.tar.gz › motif_logo/exonic_edits/TC_meme/logo6.png]

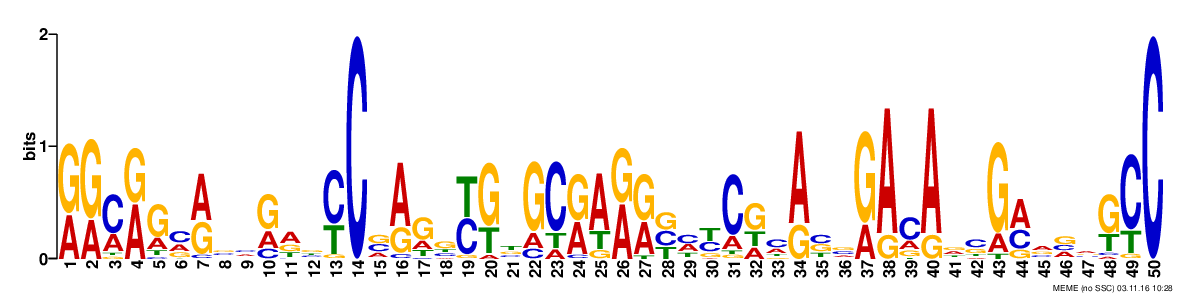

Supplement: S2 Dataset — (GZ) [file pgen.1006619.s020.tar.gz › motif_logo/exonic_edits/TC_meme/logo_rc1.png]

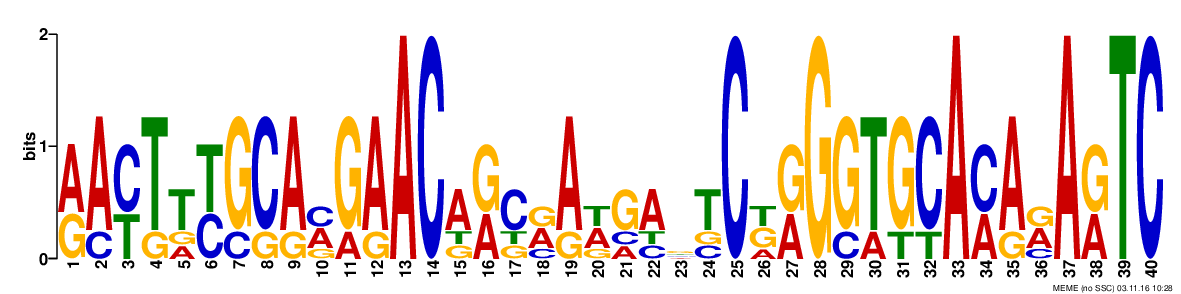

Supplement: S2 Dataset — (GZ) [file pgen.1006619.s020.tar.gz › motif_logo/exonic_edits/TC_meme/logo_rc5.png]

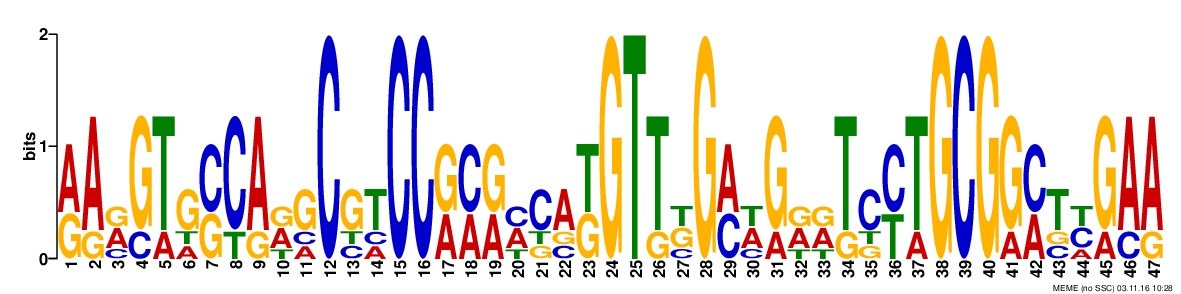

Supplement: S2 Dataset — (GZ) [file pgen.1006619.s020.tar.gz › motif_logo/exonic_edits/TC_meme/logo_rc4.png]

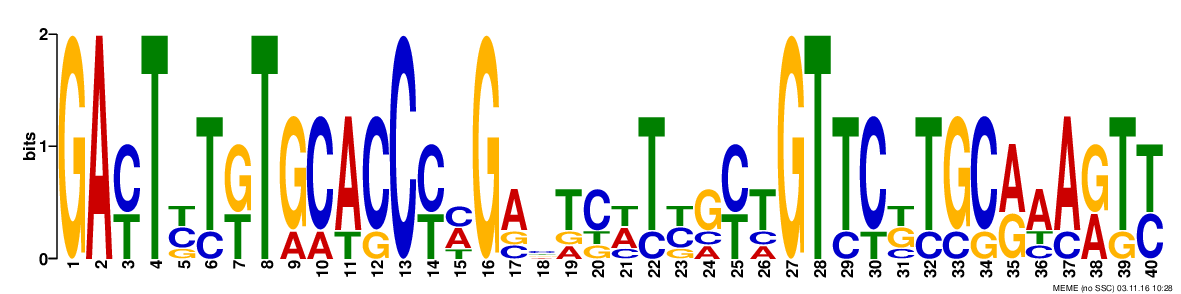

Supplement: S2 Dataset — (GZ) [file pgen.1006619.s020.tar.gz › motif_logo/exonic_edits/TC_meme/logo5.png]

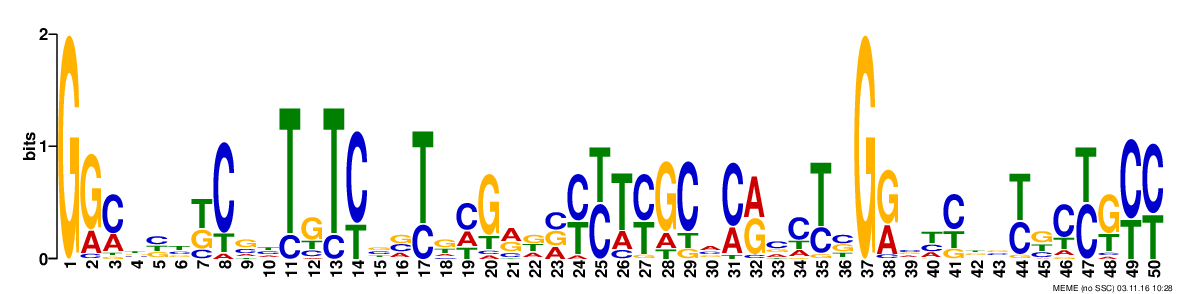

Supplement: S2 Dataset — (GZ) [file pgen.1006619.s020.tar.gz › motif_logo/exonic_edits/TC_meme/logo1.png]

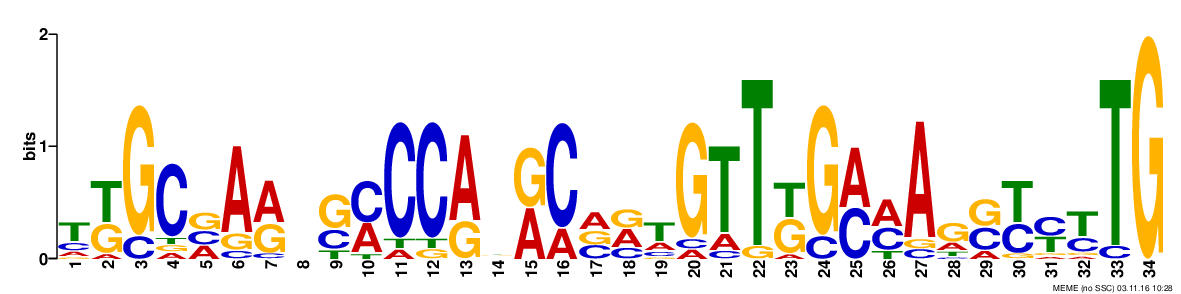

Supplement: S2 Dataset — (GZ) [file pgen.1006619.s020.tar.gz › motif_logo/exonic_edits/TC_meme/logo_rc2.png]

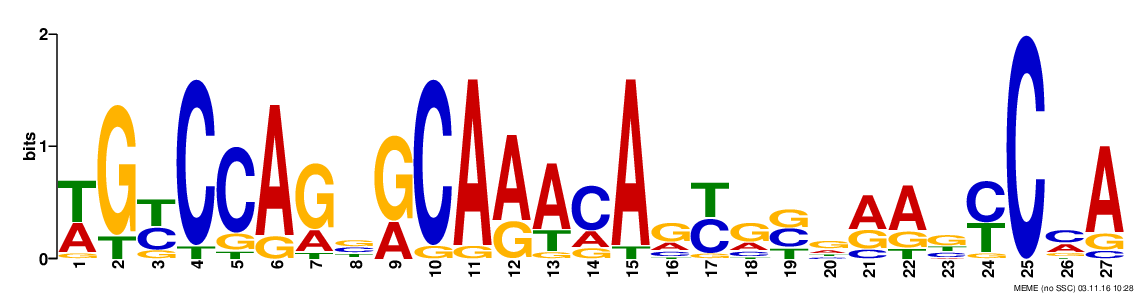

Supplement: S2 Dataset — (GZ) [file pgen.1006619.s020.tar.gz › motif_logo/exonic_edits/TC_meme/logo_rc3.png]

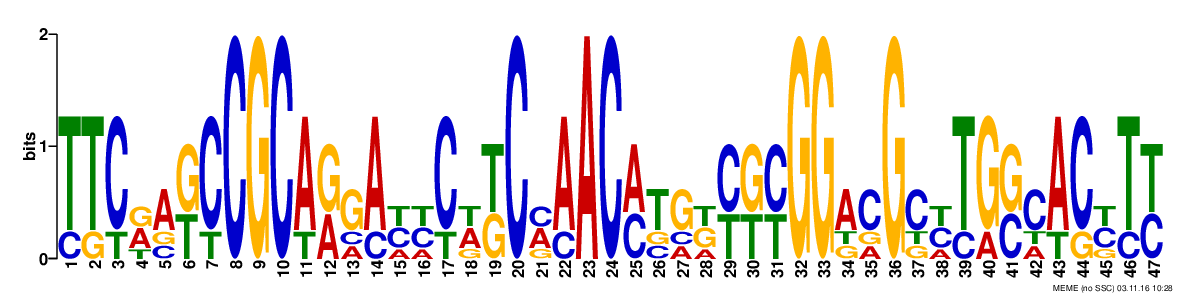

Supplement: S2 Dataset — (GZ) [file pgen.1006619.s020.tar.gz › motif_logo/exonic_edits/TC_meme/logo4.png]

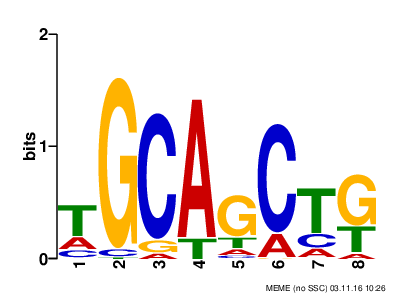

Supplement: S2 Dataset — (GZ) [file pgen.1006619.s020.tar.gz › motif_logo/exonic_edits/AG_meme/logo2.png]

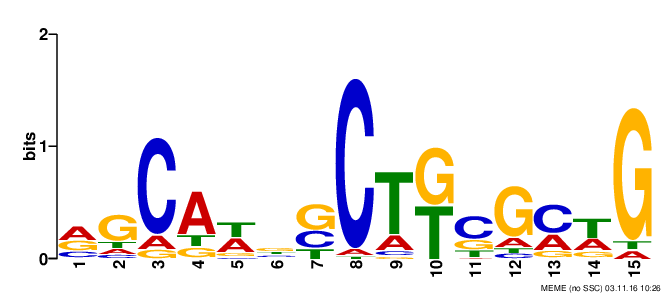

Supplement: S2 Dataset — (GZ) [file pgen.1006619.s020.tar.gz › motif_logo/exonic_edits/AG_meme/logo3.png]

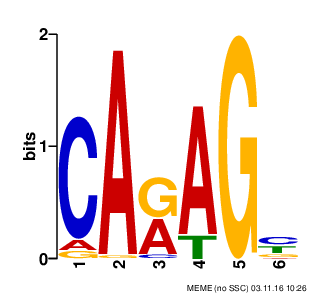

Supplement: S2 Dataset — (GZ) [file pgen.1006619.s020.tar.gz › motif_logo/exonic_edits/AG_meme/logo_rc6.png]

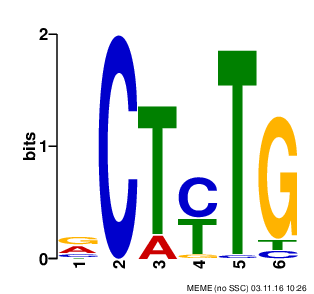

Supplement: S2 Dataset — (GZ) [file pgen.1006619.s020.tar.gz › motif_logo/exonic_edits/AG_meme/logo6.png]

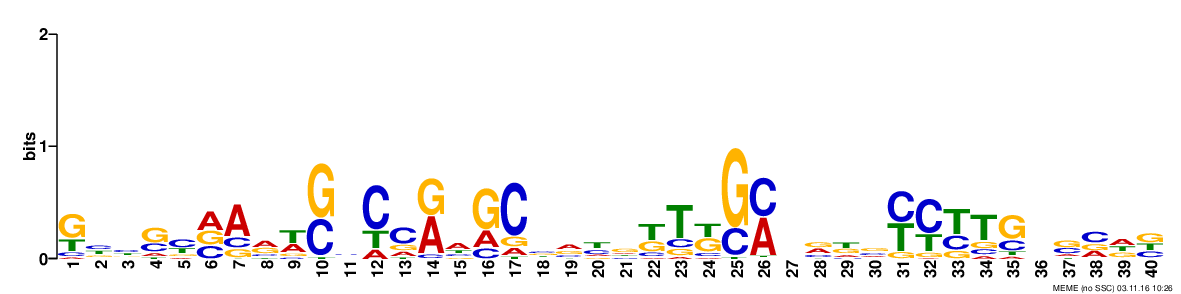

Supplement: S2 Dataset — (GZ) [file pgen.1006619.s020.tar.gz › motif_logo/exonic_edits/AG_meme/logo_rc1.png]

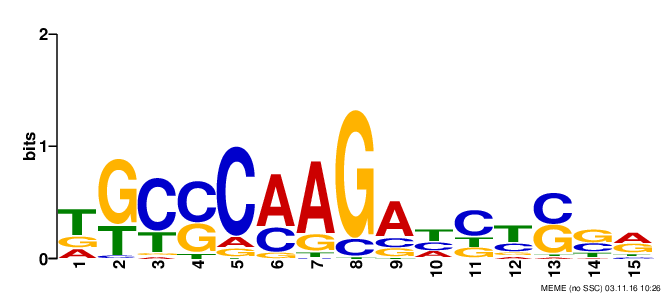

Supplement: S2 Dataset — (GZ) [file pgen.1006619.s020.tar.gz › motif_logo/exonic_edits/AG_meme/logo_rc5.png]

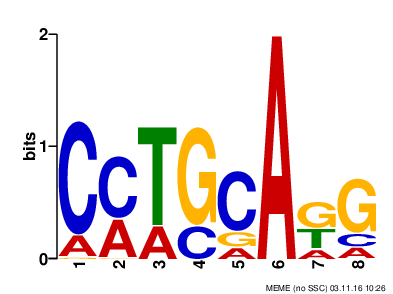

Supplement: S2 Dataset — (GZ) [file pgen.1006619.s020.tar.gz › motif_logo/exonic_edits/AG_meme/logo_rc4.png]

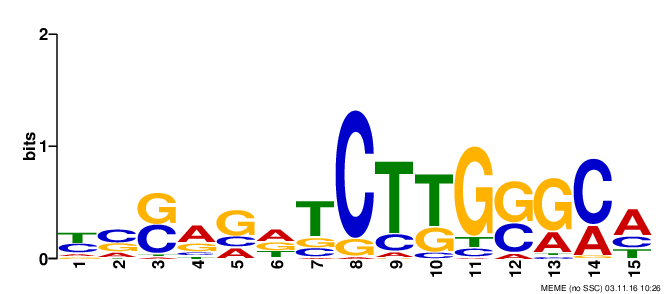

Supplement: S2 Dataset — (GZ) [file pgen.1006619.s020.tar.gz › motif_logo/exonic_edits/AG_meme/logo5.png]

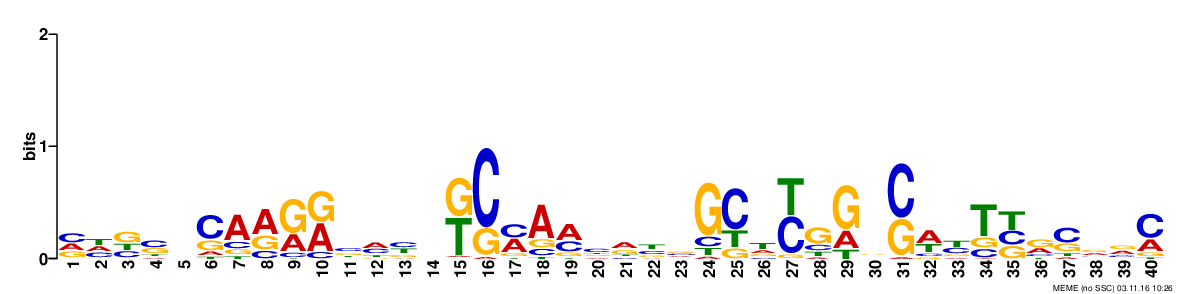

Supplement: S2 Dataset — (GZ) [file pgen.1006619.s020.tar.gz › motif_logo/exonic_edits/AG_meme/logo1.png]

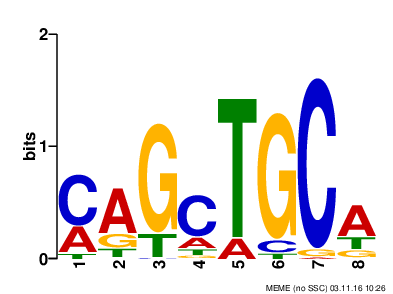

Supplement: S2 Dataset — (GZ) [file pgen.1006619.s020.tar.gz › motif_logo/exonic_edits/AG_meme/logo_rc2.png]

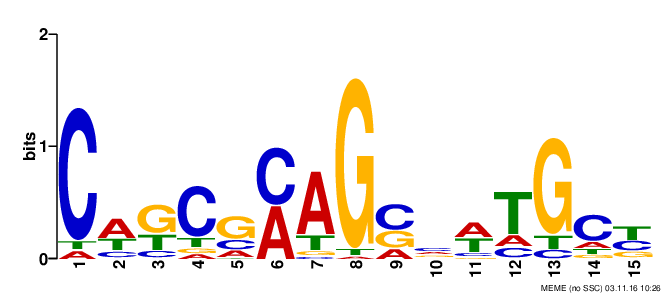

Supplement: S2 Dataset — (GZ) [file pgen.1006619.s020.tar.gz › motif_logo/exonic_edits/AG_meme/logo_rc3.png]

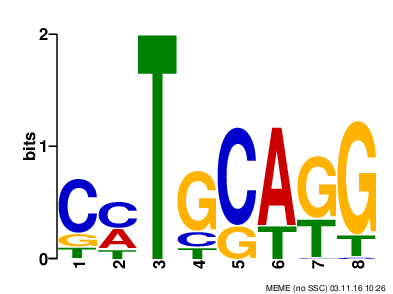

Supplement: S2 Dataset — (GZ) [file pgen.1006619.s020.tar.gz › motif_logo/exonic_edits/AG_meme/logo4.png]

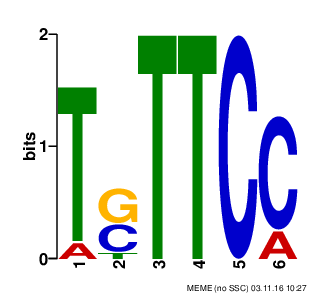

Supplement: S2 Dataset — (GZ) [file pgen.1006619.s020.tar.gz › motif_logo/exonic_edits/CG_meme/logo2.png]

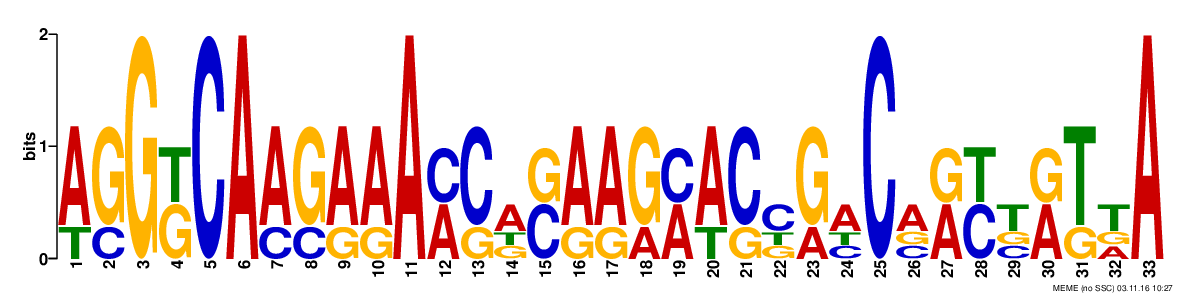

Supplement: S2 Dataset — (GZ) [file pgen.1006619.s020.tar.gz › motif_logo/exonic_edits/CG_meme/logo3.png]

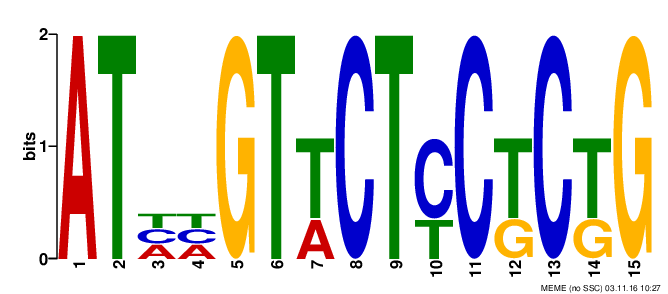

Supplement: S2 Dataset — (GZ) [file pgen.1006619.s020.tar.gz › motif_logo/exonic_edits/CG_meme/logo_rc6.png]

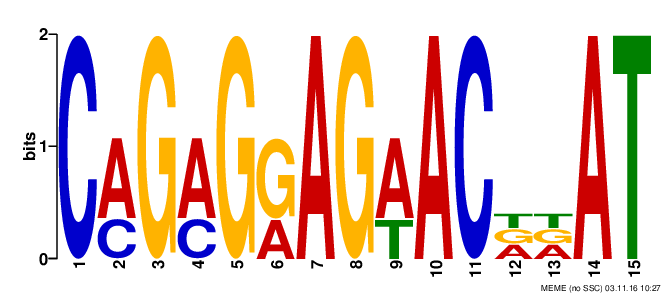

Supplement: S2 Dataset — (GZ) [file pgen.1006619.s020.tar.gz › motif_logo/exonic_edits/CG_meme/logo6.png]

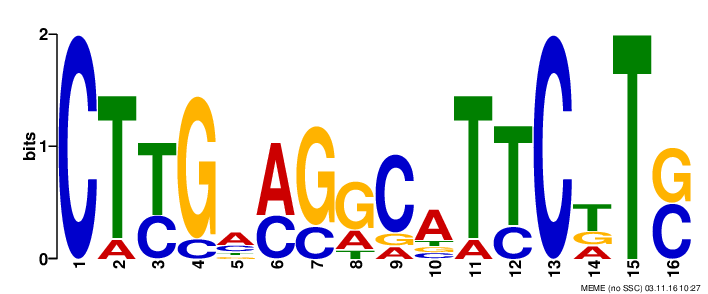

Supplement: S2 Dataset — (GZ) [file pgen.1006619.s020.tar.gz › motif_logo/exonic_edits/CG_meme/logo_rc1.png]

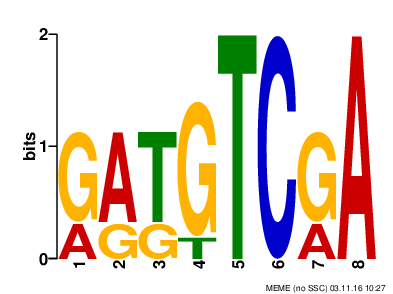

Supplement: S2 Dataset — (GZ) [file pgen.1006619.s020.tar.gz › motif_logo/exonic_edits/CG_meme/logo_rc5.png]

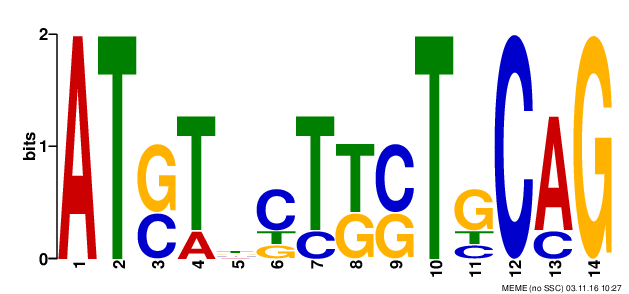

Supplement: S2 Dataset — (GZ) [file pgen.1006619.s020.tar.gz › motif_logo/exonic_edits/CG_meme/logo_rc4.png]

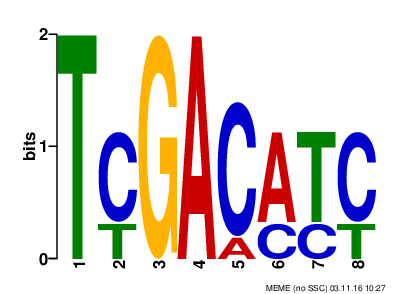

Supplement: S2 Dataset — (GZ) [file pgen.1006619.s020.tar.gz › motif_logo/exonic_edits/CG_meme/logo5.png]

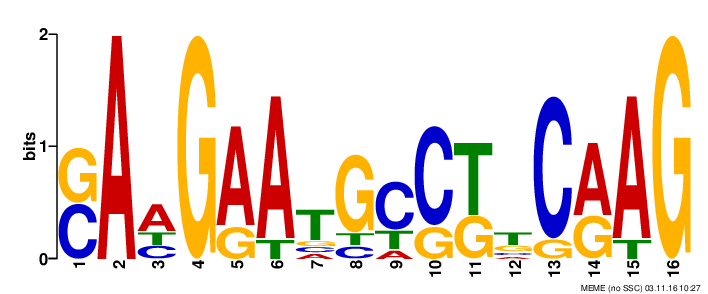

Supplement: S2 Dataset — (GZ) [file pgen.1006619.s020.tar.gz › motif_logo/exonic_edits/CG_meme/logo1.png]

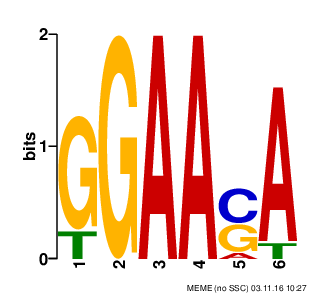

Supplement: S2 Dataset — (GZ) [file pgen.1006619.s020.tar.gz › motif_logo/exonic_edits/CG_meme/logo_rc2.png]

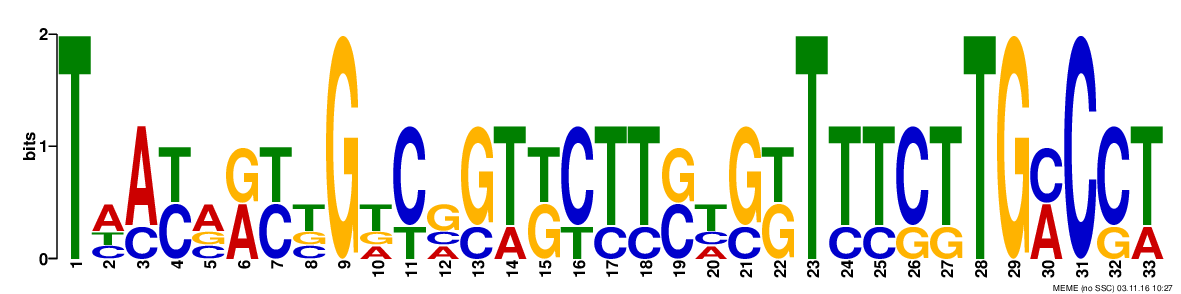

Supplement: S2 Dataset — (GZ) [file pgen.1006619.s020.tar.gz › motif_logo/exonic_edits/CG_meme/logo_rc3.png]

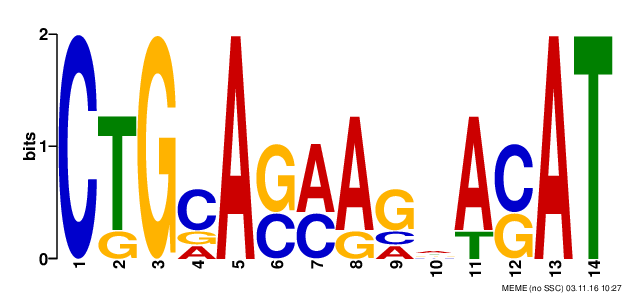

Supplement: S2 Dataset — (GZ) [file pgen.1006619.s020.tar.gz › motif_logo/exonic_edits/CG_meme/logo4.png]

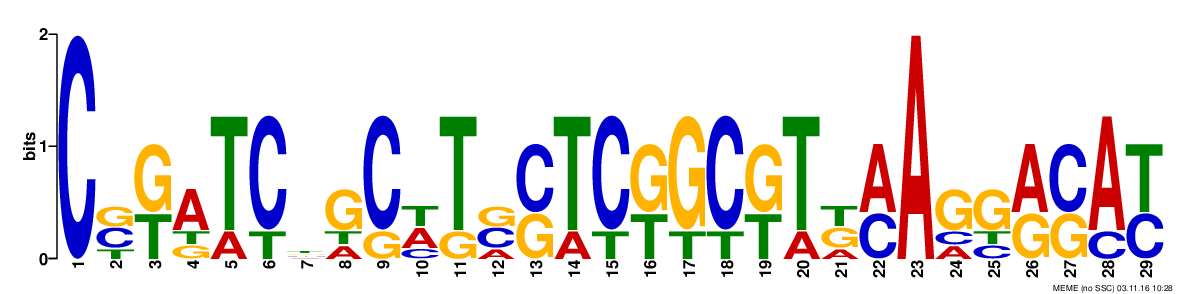

Supplement: S2 Dataset — (GZ) [file pgen.1006619.s020.tar.gz › motif_logo/exonic_edits/TG_meme/logo2.png]

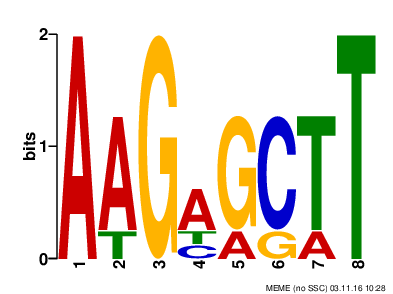

Supplement: S2 Dataset — (GZ) [file pgen.1006619.s020.tar.gz › motif_logo/exonic_edits/TG_meme/logo3.png]

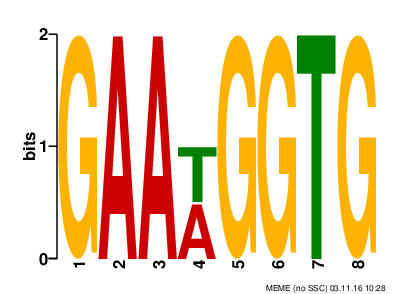

Supplement: S2 Dataset — (GZ) [file pgen.1006619.s020.tar.gz › motif_logo/exonic_edits/TG_meme/logo_rc6.png]

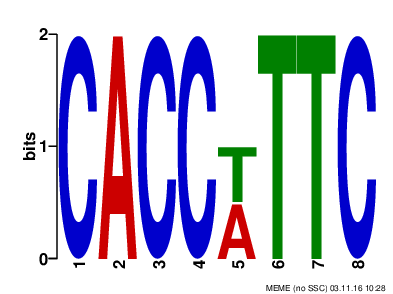

Supplement: S2 Dataset — (GZ) [file pgen.1006619.s020.tar.gz › motif_logo/exonic_edits/TG_meme/logo6.png]

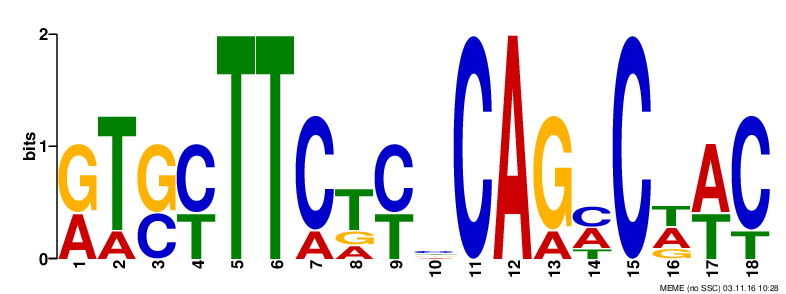

Supplement: S2 Dataset — (GZ) [file pgen.1006619.s020.tar.gz › motif_logo/exonic_edits/TG_meme/logo_rc1.png]

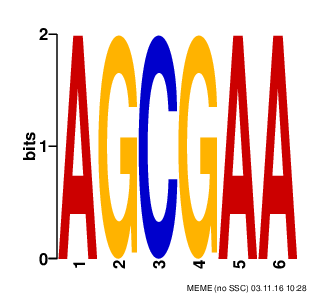

Supplement: S2 Dataset — (GZ) [file pgen.1006619.s020.tar.gz › motif_logo/exonic_edits/TG_meme/logo_rc5.png]

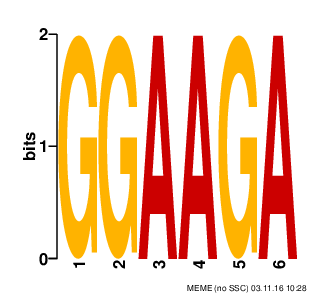

Supplement: S2 Dataset — (GZ) [file pgen.1006619.s020.tar.gz › motif_logo/exonic_edits/TG_meme/logo_rc4.png]

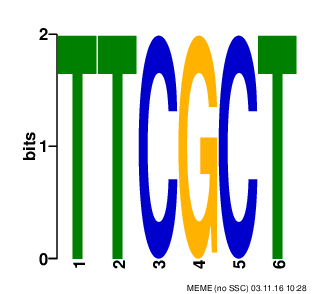

Supplement: S2 Dataset — (GZ) [file pgen.1006619.s020.tar.gz › motif_logo/exonic_edits/TG_meme/logo5.png]
