# Supplementary figures and images for: Condition-specific RNA editing in the coral symbiont Symbiodinium microadriaticum (part 2 of 2)
Source: PLoS Genet. 2017 Feb 28;13(2):e1006619. doi: 10.1371/journal.pgen.1006619 (PMC5357065; doi:10.1371/journal.pgen.1006619)

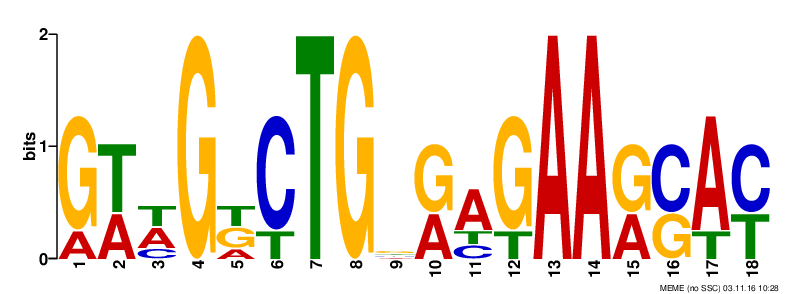

Supplement: S2 Dataset — (GZ) [file pgen.1006619.s020.tar.gz › motif_logo/exonic_edits/TG_meme/logo1.png]

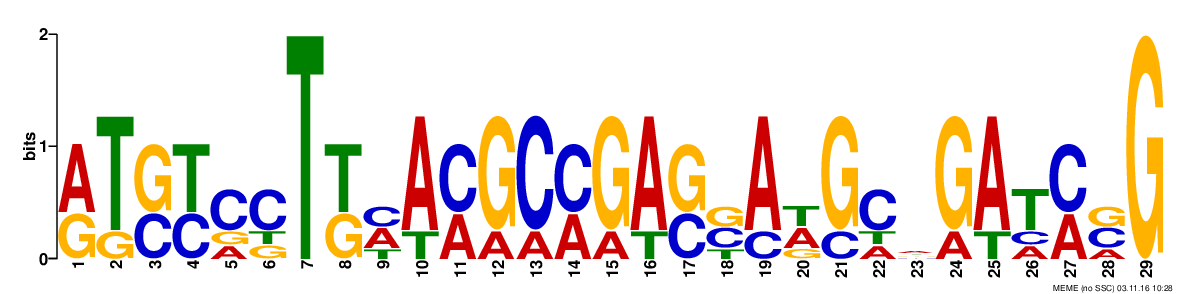

Supplement: S2 Dataset — (GZ) [file pgen.1006619.s020.tar.gz › motif_logo/exonic_edits/TG_meme/logo_rc2.png]

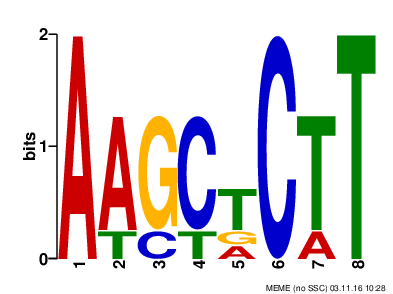

Supplement: S2 Dataset — (GZ) [file pgen.1006619.s020.tar.gz › motif_logo/exonic_edits/TG_meme/logo_rc3.png]

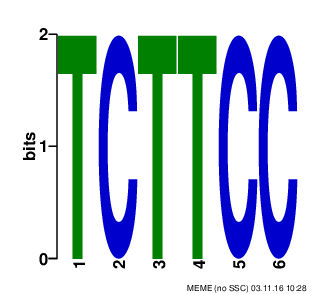

Supplement: S2 Dataset — (GZ) [file pgen.1006619.s020.tar.gz › motif_logo/exonic_edits/TG_meme/logo4.png]

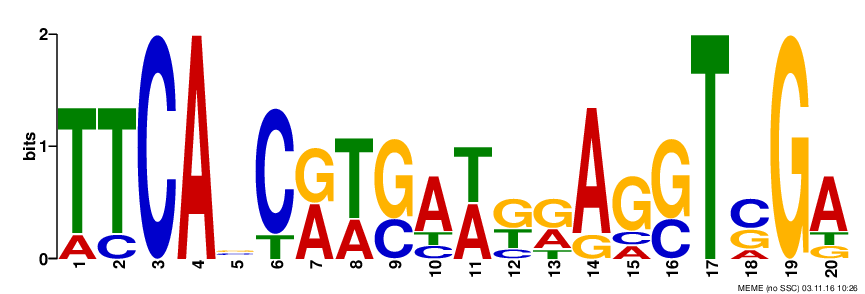

Supplement: S2 Dataset — (GZ) [file pgen.1006619.s020.tar.gz › motif_logo/exonic_edits/AC_meme/logo2.png]

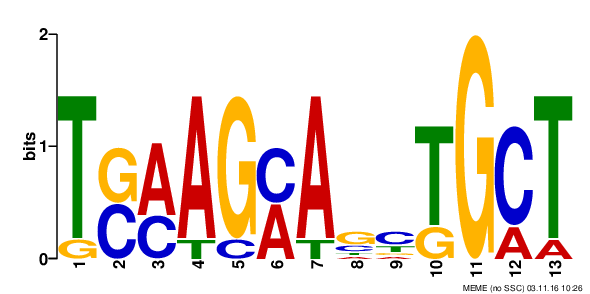

Supplement: S2 Dataset — (GZ) [file pgen.1006619.s020.tar.gz › motif_logo/exonic_edits/AC_meme/logo3.png]

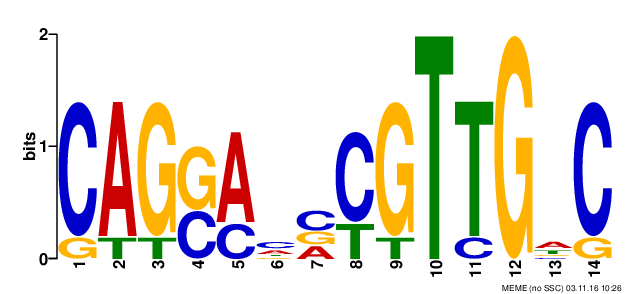

Supplement: S2 Dataset — (GZ) [file pgen.1006619.s020.tar.gz › motif_logo/exonic_edits/AC_meme/logo_rc6.png]

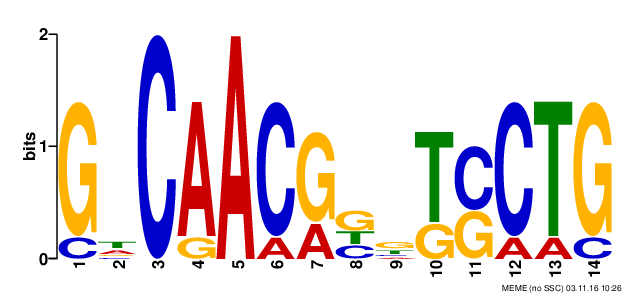

Supplement: S2 Dataset — (GZ) [file pgen.1006619.s020.tar.gz › motif_logo/exonic_edits/AC_meme/logo6.png]

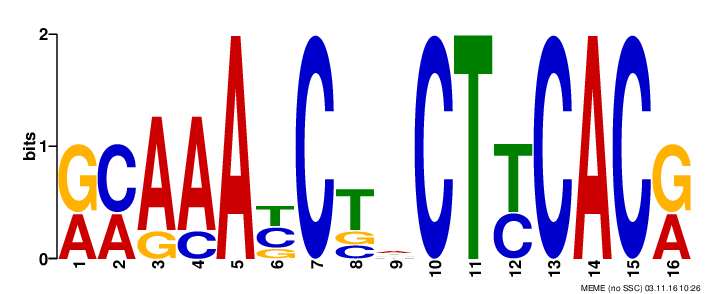

Supplement: S2 Dataset — (GZ) [file pgen.1006619.s020.tar.gz › motif_logo/exonic_edits/AC_meme/logo_rc1.png]

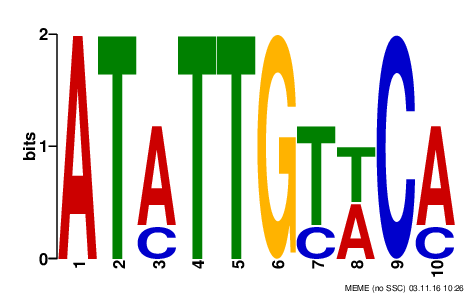

Supplement: S2 Dataset — (GZ) [file pgen.1006619.s020.tar.gz › motif_logo/exonic_edits/AC_meme/logo_rc5.png]

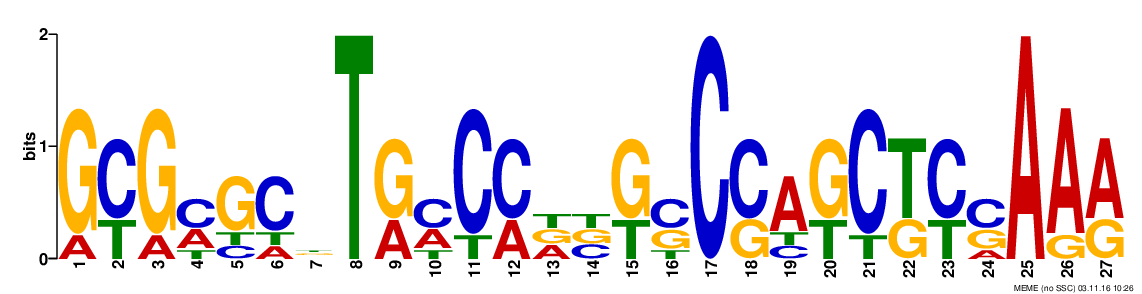

Supplement: S2 Dataset — (GZ) [file pgen.1006619.s020.tar.gz › motif_logo/exonic_edits/AC_meme/logo_rc4.png]

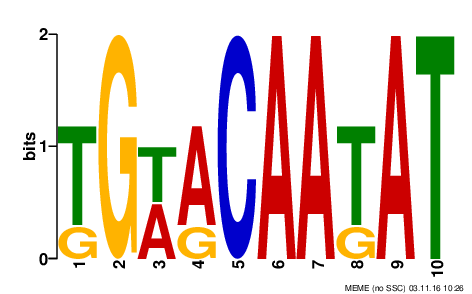

Supplement: S2 Dataset — (GZ) [file pgen.1006619.s020.tar.gz › motif_logo/exonic_edits/AC_meme/logo5.png]

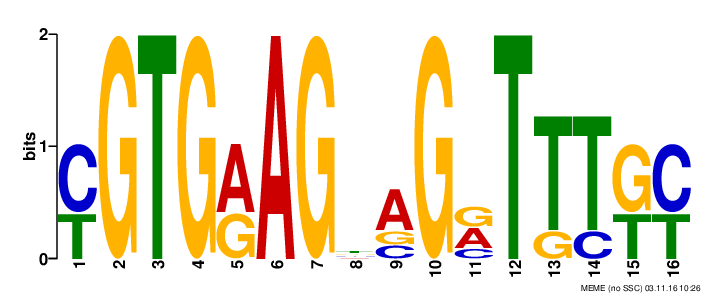

Supplement: S2 Dataset — (GZ) [file pgen.1006619.s020.tar.gz › motif_logo/exonic_edits/AC_meme/logo1.png]

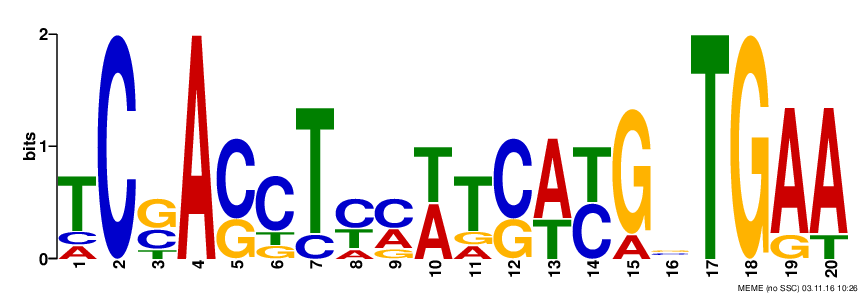

Supplement: S2 Dataset — (GZ) [file pgen.1006619.s020.tar.gz › motif_logo/exonic_edits/AC_meme/logo_rc2.png]

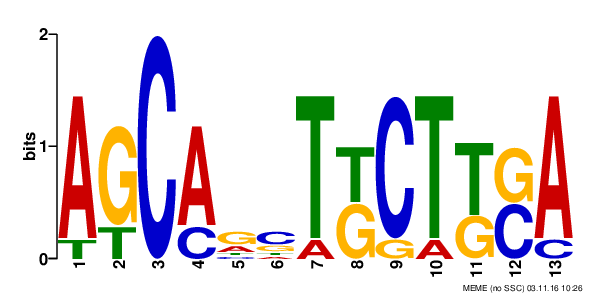

Supplement: S2 Dataset — (GZ) [file pgen.1006619.s020.tar.gz › motif_logo/exonic_edits/AC_meme/logo_rc3.png]

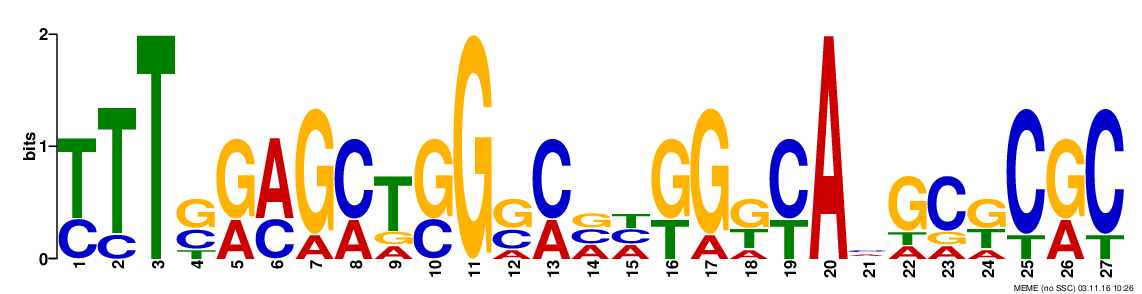

Supplement: S2 Dataset — (GZ) [file pgen.1006619.s020.tar.gz › motif_logo/exonic_edits/AC_meme/logo4.png]

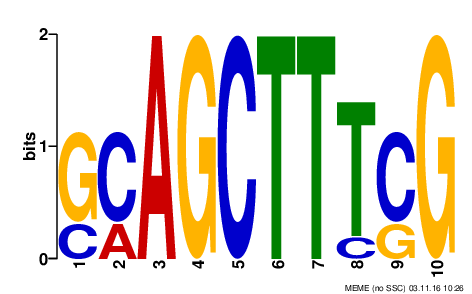

Supplement: S2 Dataset — (GZ) [file pgen.1006619.s020.tar.gz › motif_logo/exonic_edits/AT_meme/logo2.png]

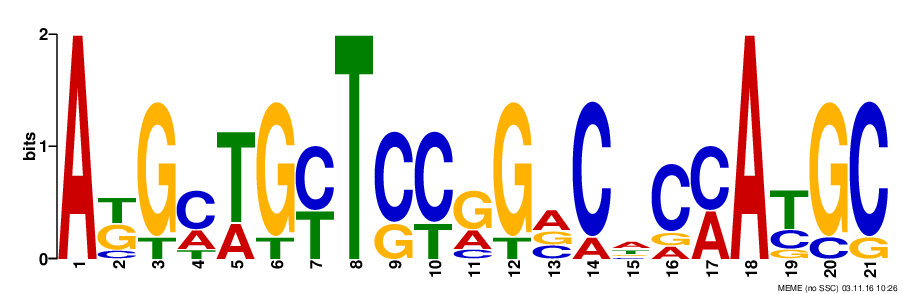

Supplement: S2 Dataset — (GZ) [file pgen.1006619.s020.tar.gz › motif_logo/exonic_edits/AT_meme/logo3.png]

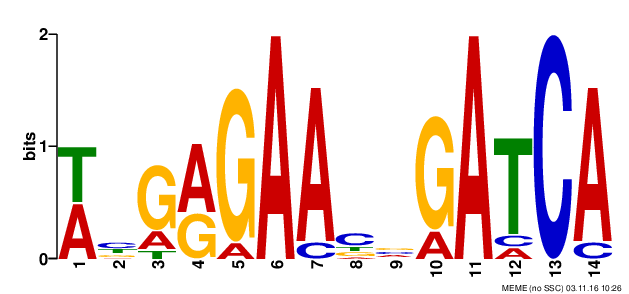

Supplement: S2 Dataset — (GZ) [file pgen.1006619.s020.tar.gz › motif_logo/exonic_edits/AT_meme/logo_rc6.png]

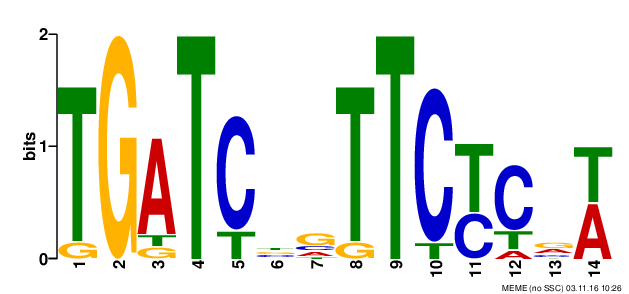

Supplement: S2 Dataset — (GZ) [file pgen.1006619.s020.tar.gz › motif_logo/exonic_edits/AT_meme/logo6.png]

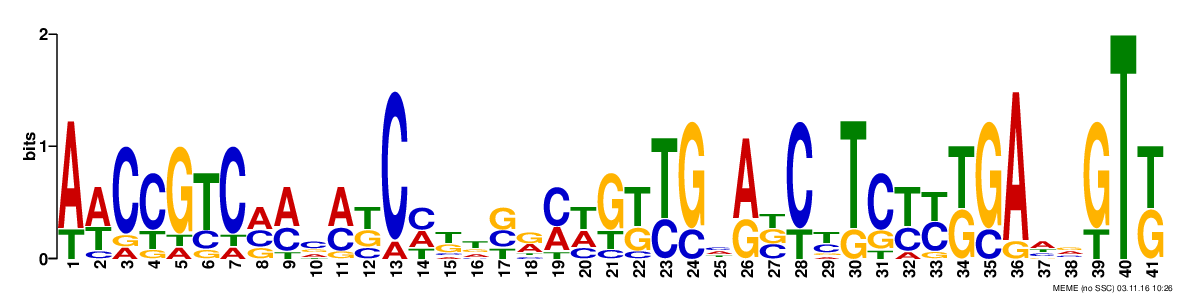

Supplement: S2 Dataset — (GZ) [file pgen.1006619.s020.tar.gz › motif_logo/exonic_edits/AT_meme/logo_rc1.png]

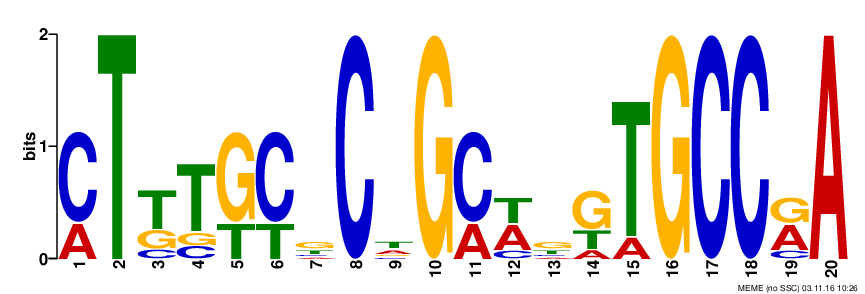

Supplement: S2 Dataset — (GZ) [file pgen.1006619.s020.tar.gz › motif_logo/exonic_edits/AT_meme/logo_rc5.png]

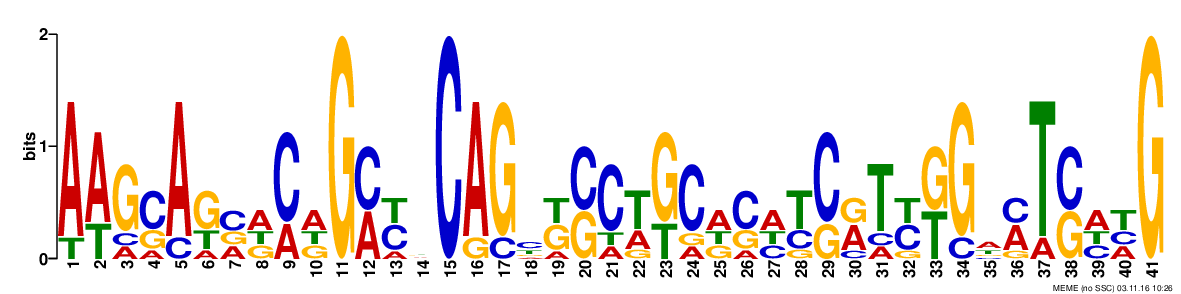

Supplement: S2 Dataset — (GZ) [file pgen.1006619.s020.tar.gz › motif_logo/exonic_edits/AT_meme/logo_rc4.png]

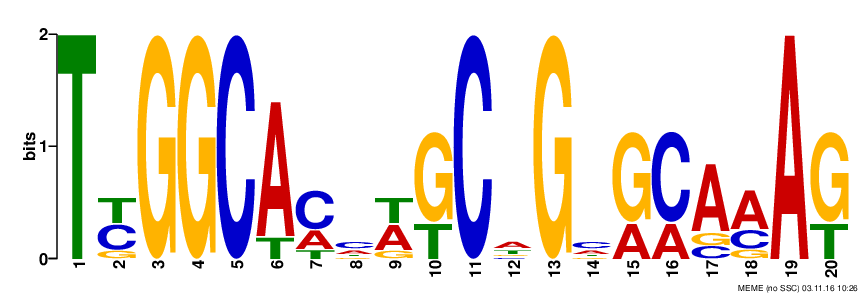

Supplement: S2 Dataset — (GZ) [file pgen.1006619.s020.tar.gz › motif_logo/exonic_edits/AT_meme/logo5.png]

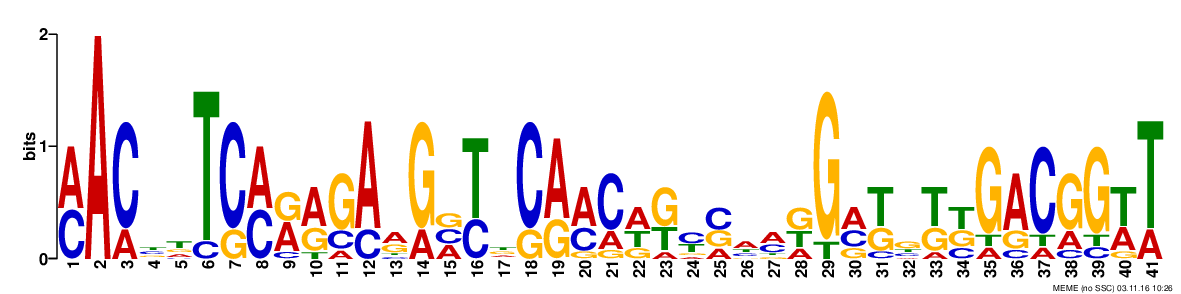

Supplement: S2 Dataset — (GZ) [file pgen.1006619.s020.tar.gz › motif_logo/exonic_edits/AT_meme/logo1.png]

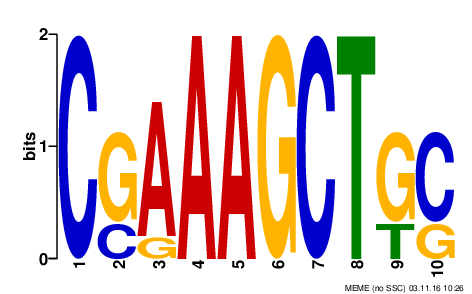

Supplement: S2 Dataset — (GZ) [file pgen.1006619.s020.tar.gz › motif_logo/exonic_edits/AT_meme/logo_rc2.png]

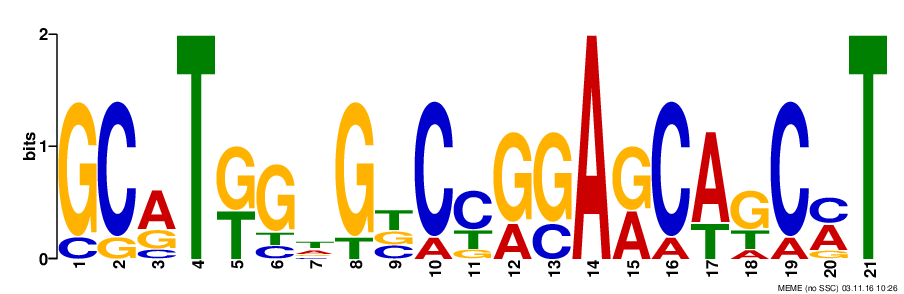

Supplement: S2 Dataset — (GZ) [file pgen.1006619.s020.tar.gz › motif_logo/exonic_edits/AT_meme/logo_rc3.png]

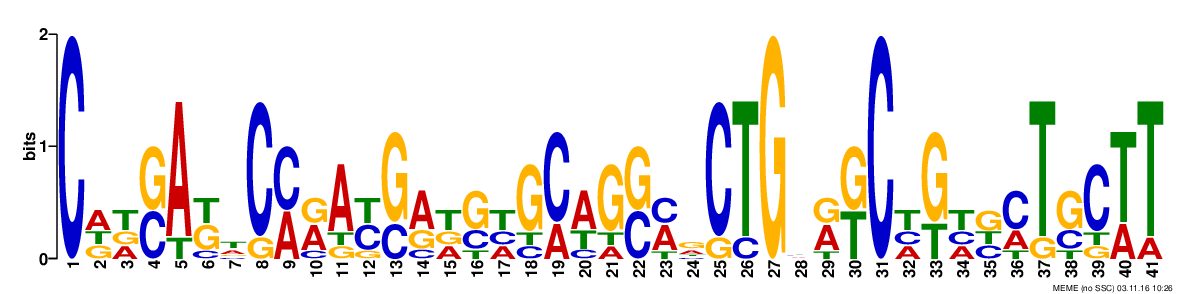

Supplement: S2 Dataset — (GZ) [file pgen.1006619.s020.tar.gz › motif_logo/exonic_edits/AT_meme/logo4.png]

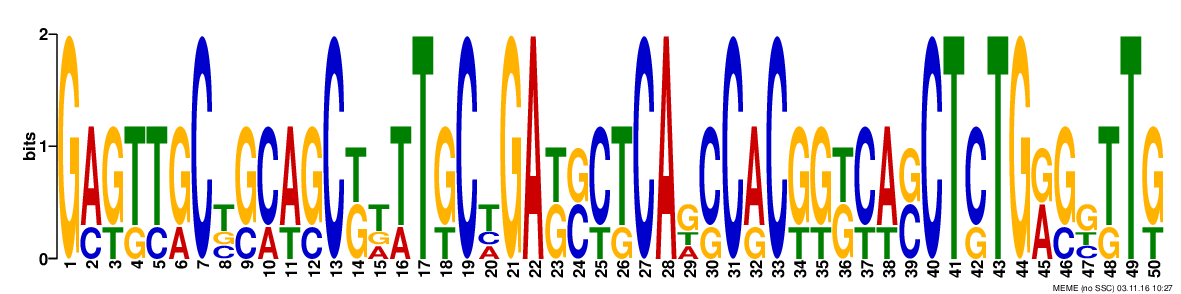

Supplement: S2 Dataset — (GZ) [file pgen.1006619.s020.tar.gz › motif_logo/exonic_edits/GA_meme/logo2.png]

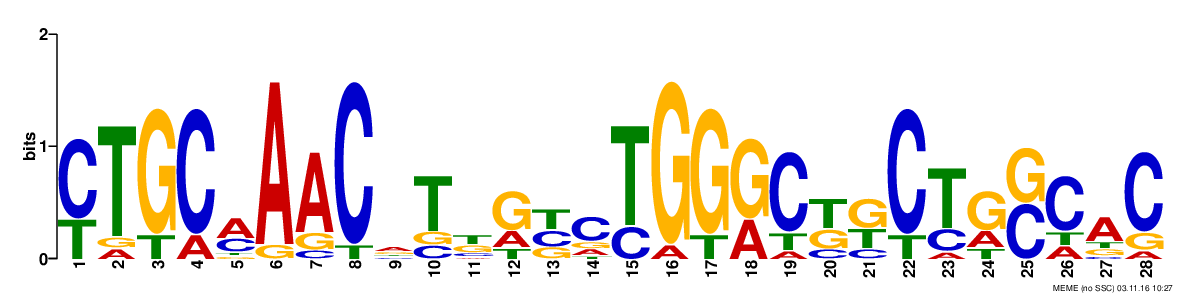

Supplement: S2 Dataset — (GZ) [file pgen.1006619.s020.tar.gz › motif_logo/exonic_edits/GA_meme/logo3.png]

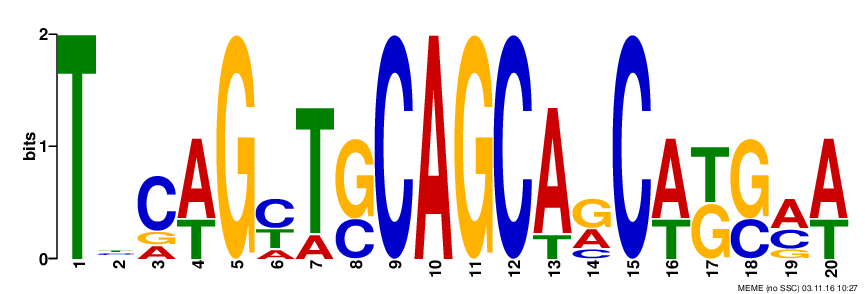

Supplement: S2 Dataset — (GZ) [file pgen.1006619.s020.tar.gz › motif_logo/exonic_edits/GA_meme/logo_rc6.png]

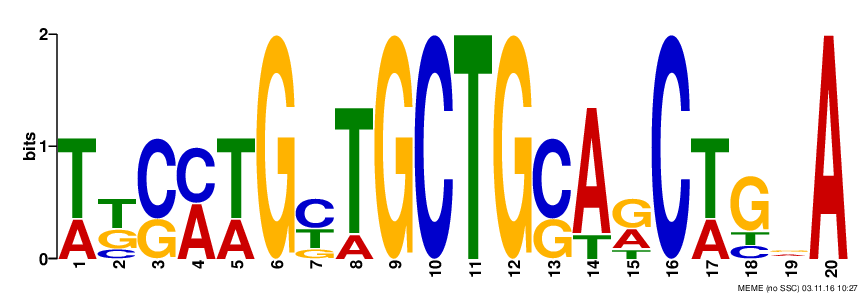

Supplement: S2 Dataset — (GZ) [file pgen.1006619.s020.tar.gz › motif_logo/exonic_edits/GA_meme/logo6.png]

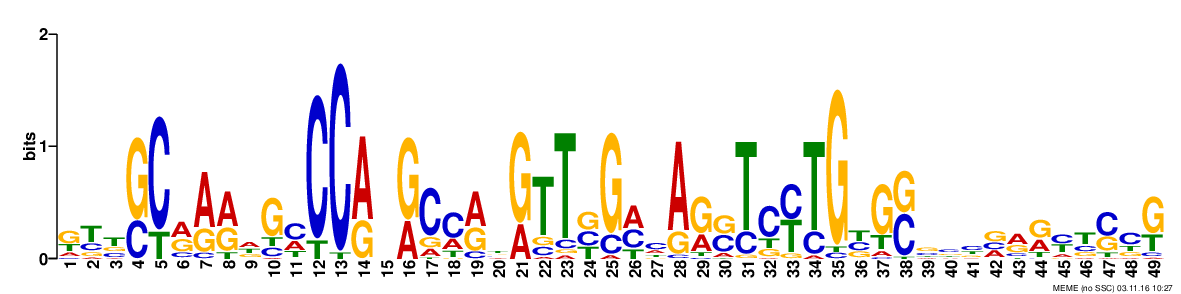

Supplement: S2 Dataset — (GZ) [file pgen.1006619.s020.tar.gz › motif_logo/exonic_edits/GA_meme/logo_rc1.png]

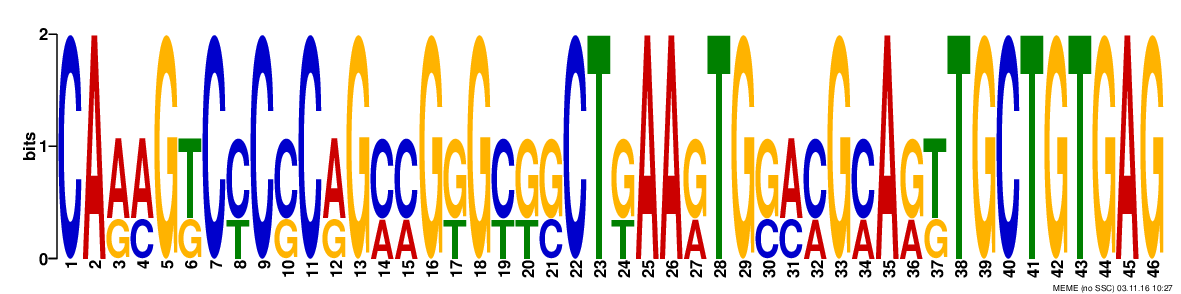

Supplement: S2 Dataset — (GZ) [file pgen.1006619.s020.tar.gz › motif_logo/exonic_edits/GA_meme/logo_rc5.png]

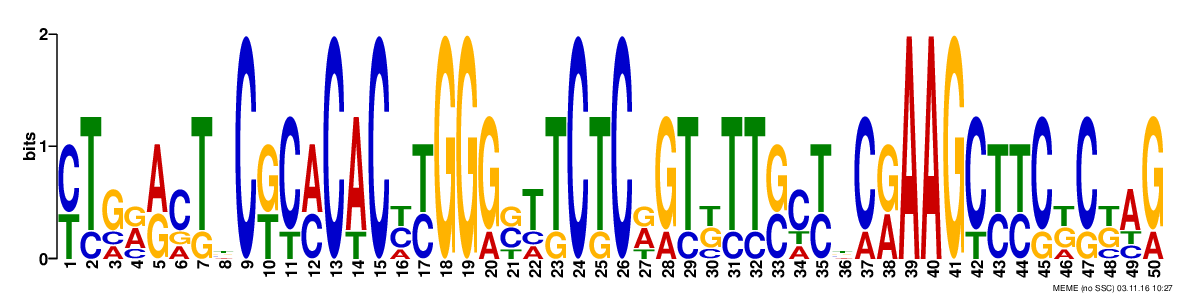

Supplement: S2 Dataset — (GZ) [file pgen.1006619.s020.tar.gz › motif_logo/exonic_edits/GA_meme/logo_rc4.png]

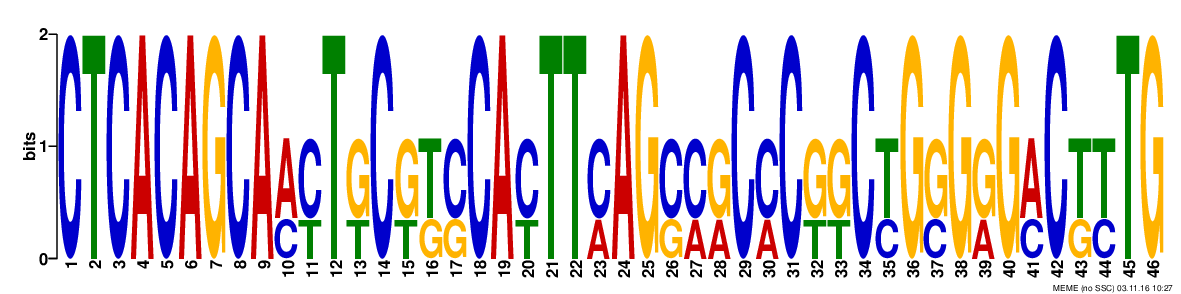

Supplement: S2 Dataset — (GZ) [file pgen.1006619.s020.tar.gz › motif_logo/exonic_edits/GA_meme/logo5.png]

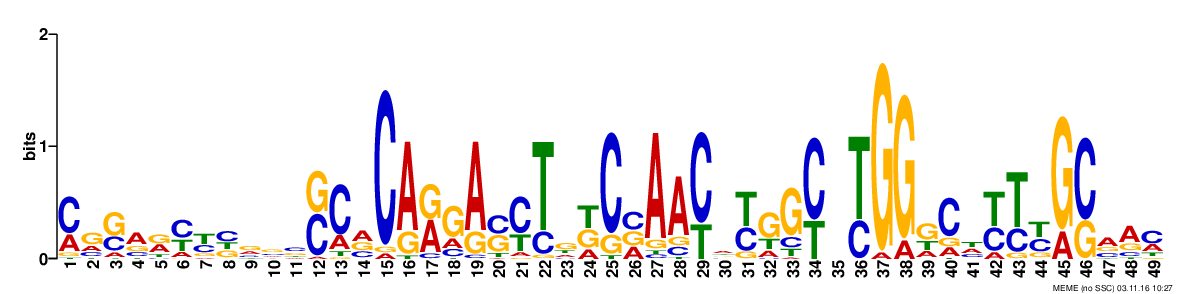

Supplement: S2 Dataset — (GZ) [file pgen.1006619.s020.tar.gz › motif_logo/exonic_edits/GA_meme/logo1.png]

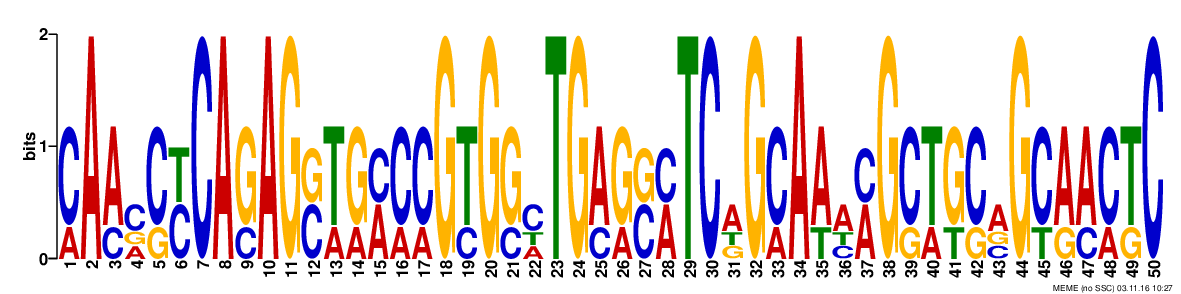

Supplement: S2 Dataset — (GZ) [file pgen.1006619.s020.tar.gz › motif_logo/exonic_edits/GA_meme/logo_rc2.png]

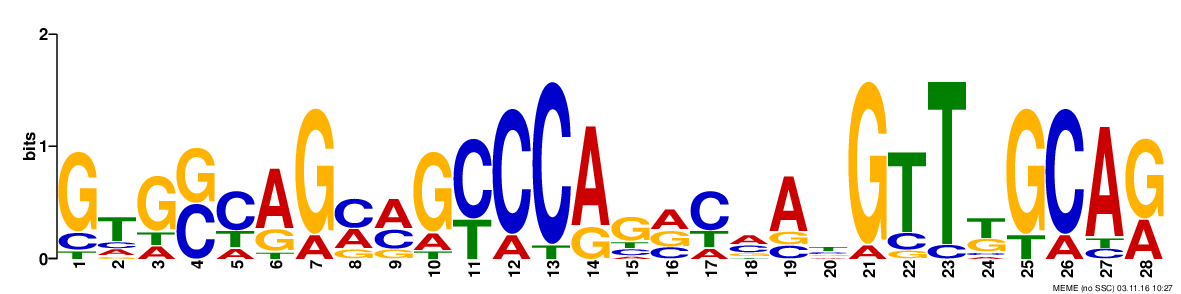

Supplement: S2 Dataset — (GZ) [file pgen.1006619.s020.tar.gz › motif_logo/exonic_edits/GA_meme/logo_rc3.png]

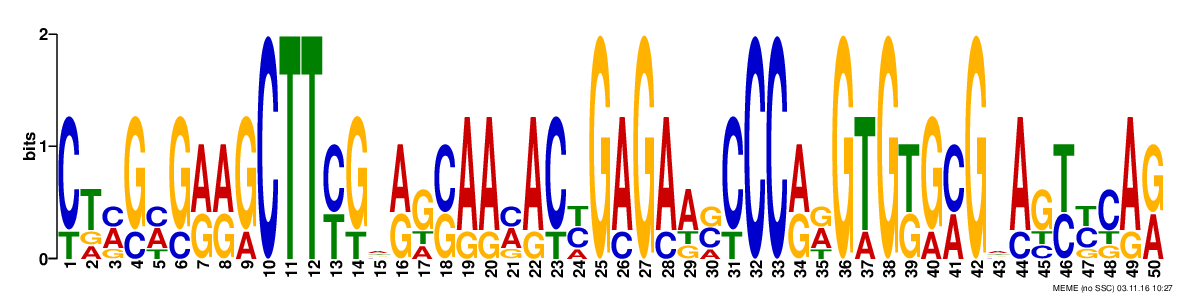

Supplement: S2 Dataset — (GZ) [file pgen.1006619.s020.tar.gz › motif_logo/exonic_edits/GA_meme/logo4.png]

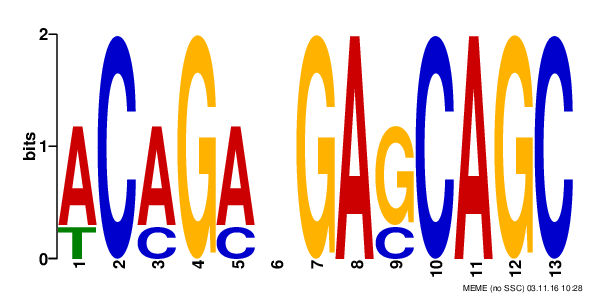

Supplement: S2 Dataset — (GZ) [file pgen.1006619.s020.tar.gz › motif_logo/exonic_edits/GT_meme/logo2.png]

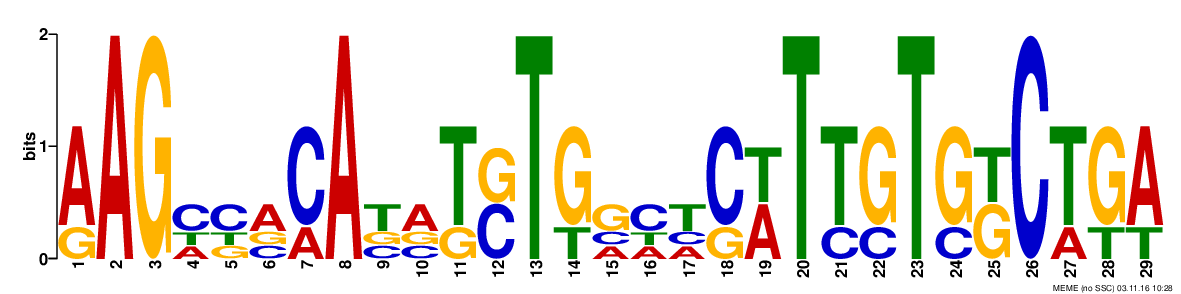

Supplement: S2 Dataset — (GZ) [file pgen.1006619.s020.tar.gz › motif_logo/exonic_edits/GT_meme/logo3.png]

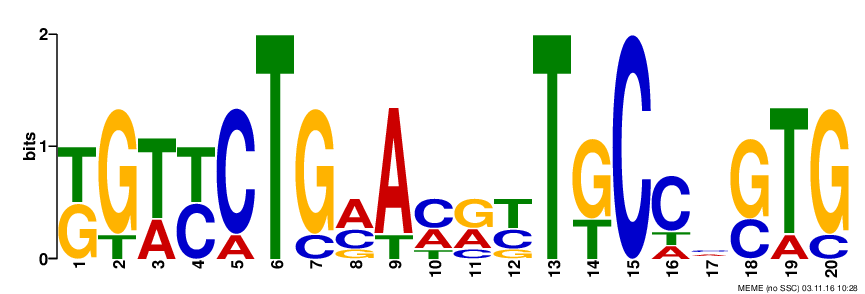

Supplement: S2 Dataset — (GZ) [file pgen.1006619.s020.tar.gz › motif_logo/exonic_edits/GT_meme/logo_rc6.png]

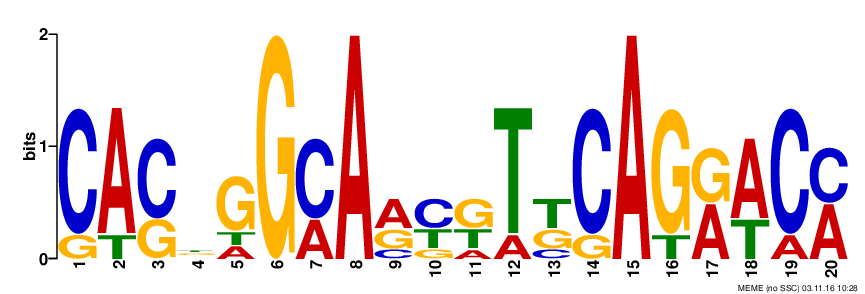

Supplement: S2 Dataset — (GZ) [file pgen.1006619.s020.tar.gz › motif_logo/exonic_edits/GT_meme/logo6.png]

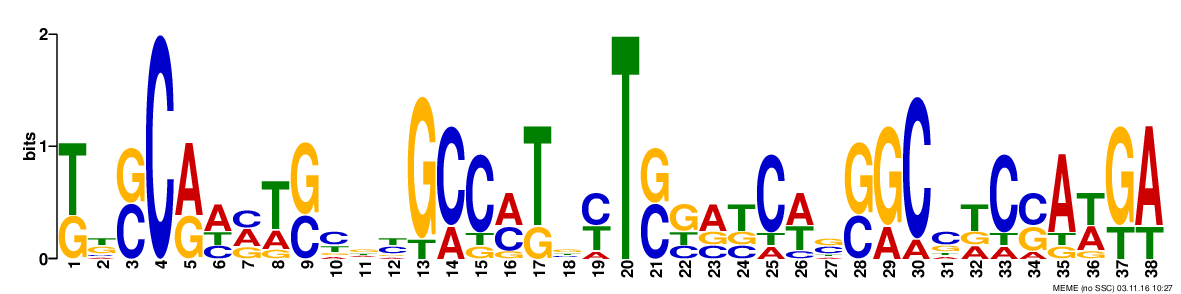

Supplement: S2 Dataset — (GZ) [file pgen.1006619.s020.tar.gz › motif_logo/exonic_edits/GT_meme/logo_rc1.png]

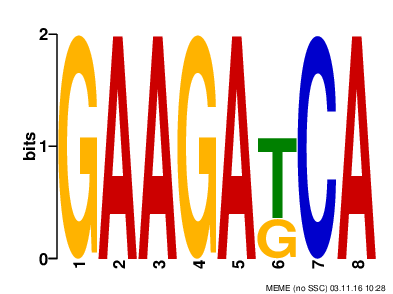

Supplement: S2 Dataset — (GZ) [file pgen.1006619.s020.tar.gz › motif_logo/exonic_edits/GT_meme/logo_rc5.png]

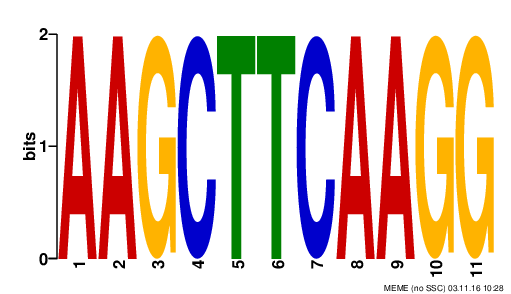

Supplement: S2 Dataset — (GZ) [file pgen.1006619.s020.tar.gz › motif_logo/exonic_edits/GT_meme/logo_rc4.png]

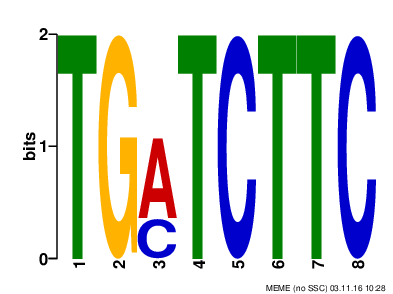

Supplement: S2 Dataset — (GZ) [file pgen.1006619.s020.tar.gz › motif_logo/exonic_edits/GT_meme/logo5.png]

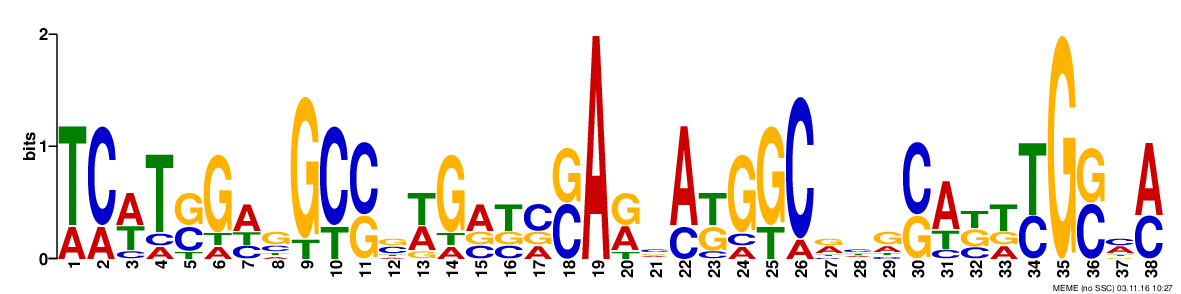

Supplement: S2 Dataset — (GZ) [file pgen.1006619.s020.tar.gz › motif_logo/exonic_edits/GT_meme/logo1.png]

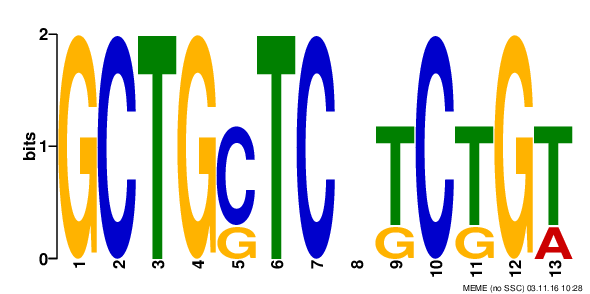

Supplement: S2 Dataset — (GZ) [file pgen.1006619.s020.tar.gz › motif_logo/exonic_edits/GT_meme/logo_rc2.png]

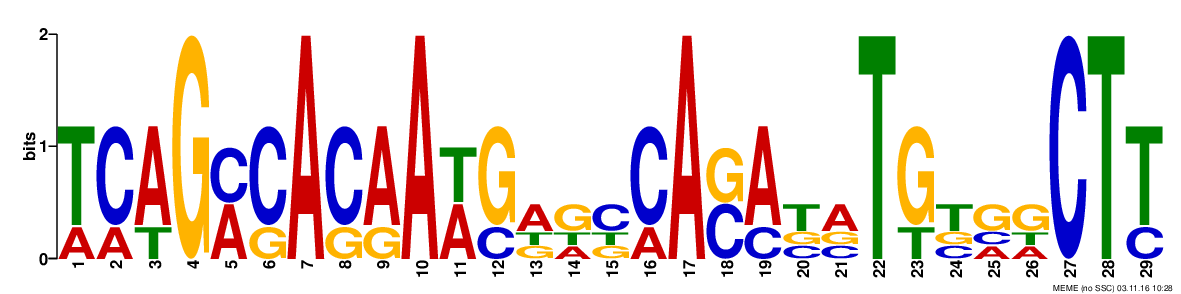

Supplement: S2 Dataset — (GZ) [file pgen.1006619.s020.tar.gz › motif_logo/exonic_edits/GT_meme/logo_rc3.png]

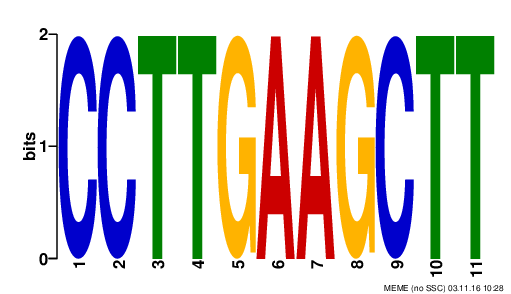

Supplement: S2 Dataset — (GZ) [file pgen.1006619.s020.tar.gz › motif_logo/exonic_edits/GT_meme/logo4.png]

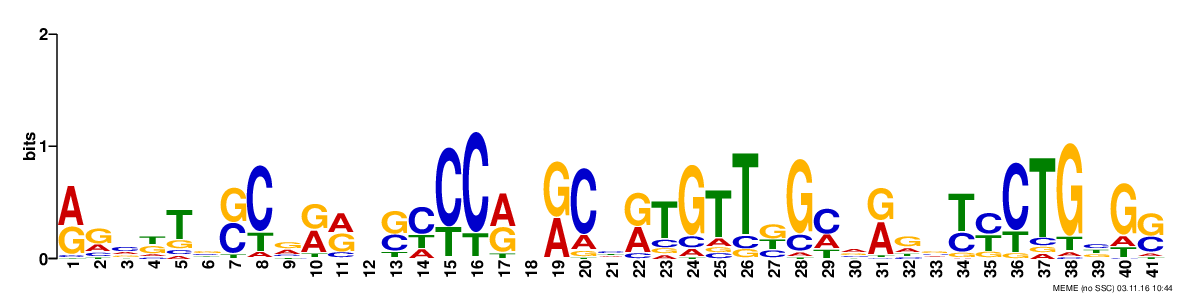

Supplement: S2 Dataset — (GZ) [file pgen.1006619.s020.tar.gz › motif_logo/genic_edits/CT_meme/logo2.png]

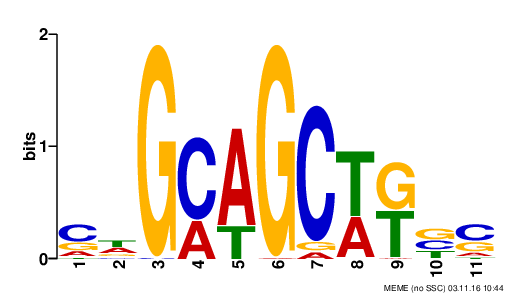

Supplement: S2 Dataset — (GZ) [file pgen.1006619.s020.tar.gz › motif_logo/genic_edits/CT_meme/logo3.png]

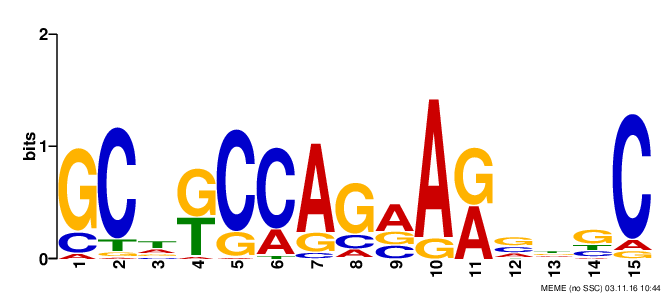

Supplement: S2 Dataset — (GZ) [file pgen.1006619.s020.tar.gz › motif_logo/genic_edits/CT_meme/logo_rc6.png]

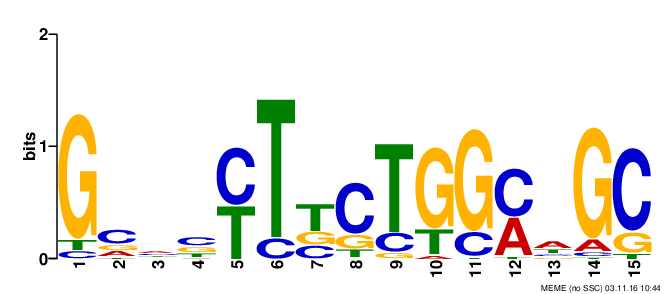

Supplement: S2 Dataset — (GZ) [file pgen.1006619.s020.tar.gz › motif_logo/genic_edits/CT_meme/logo6.png]

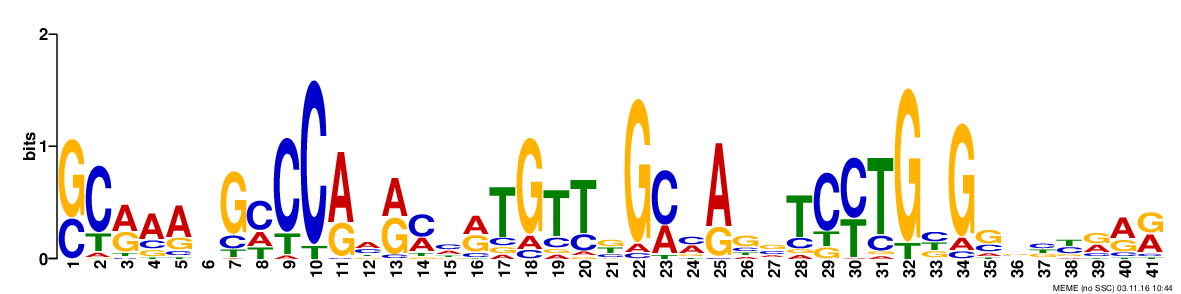

Supplement: S2 Dataset — (GZ) [file pgen.1006619.s020.tar.gz › motif_logo/genic_edits/CT_meme/logo_rc1.png]

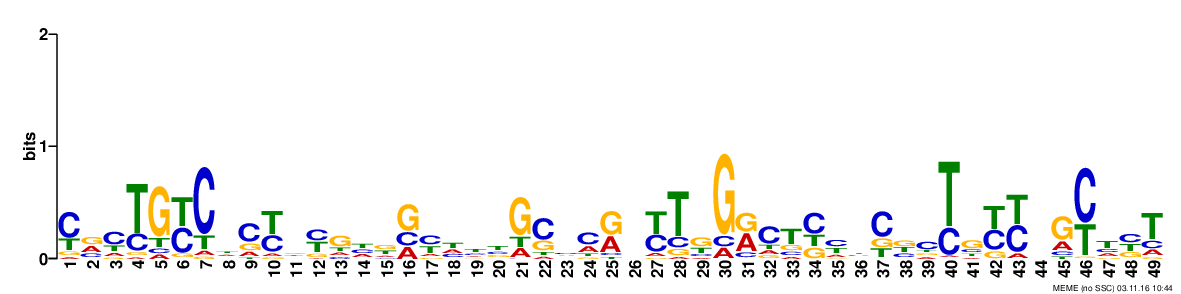

Supplement: S2 Dataset — (GZ) [file pgen.1006619.s020.tar.gz › motif_logo/genic_edits/CT_meme/logo_rc5.png]

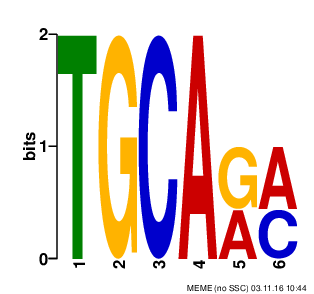

Supplement: S2 Dataset — (GZ) [file pgen.1006619.s020.tar.gz › motif_logo/genic_edits/CT_meme/logo_rc4.png]

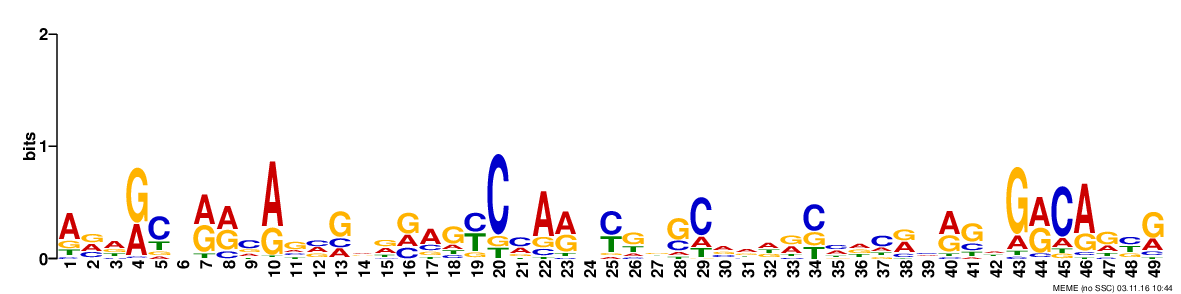

Supplement: S2 Dataset — (GZ) [file pgen.1006619.s020.tar.gz › motif_logo/genic_edits/CT_meme/logo5.png]

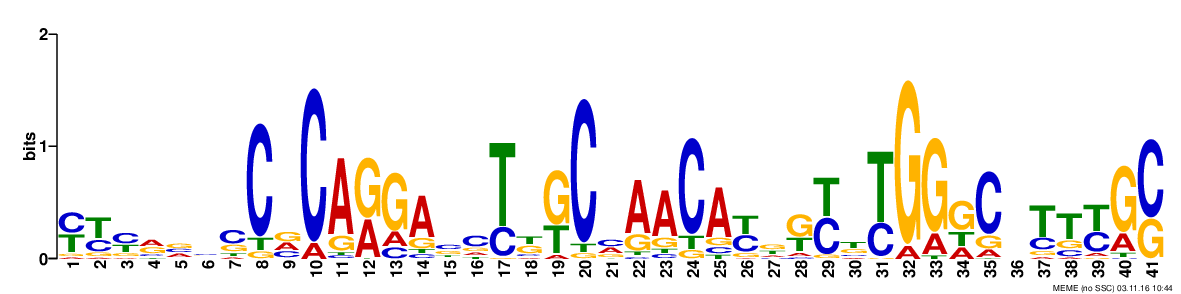

Supplement: S2 Dataset — (GZ) [file pgen.1006619.s020.tar.gz › motif_logo/genic_edits/CT_meme/logo1.png]

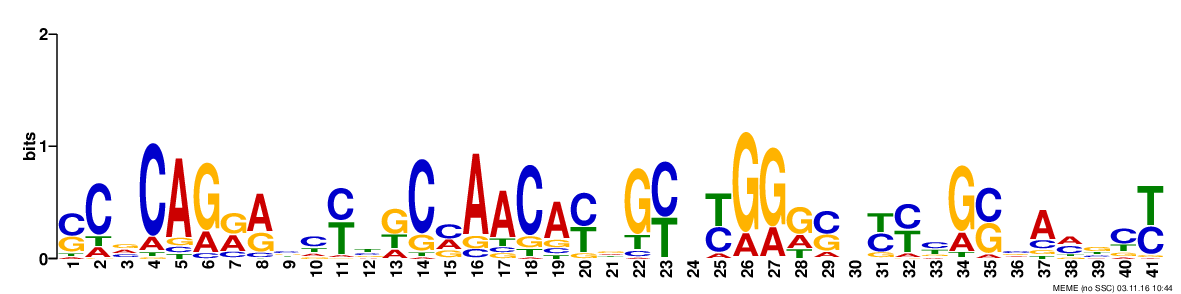

Supplement: S2 Dataset — (GZ) [file pgen.1006619.s020.tar.gz › motif_logo/genic_edits/CT_meme/logo_rc2.png]

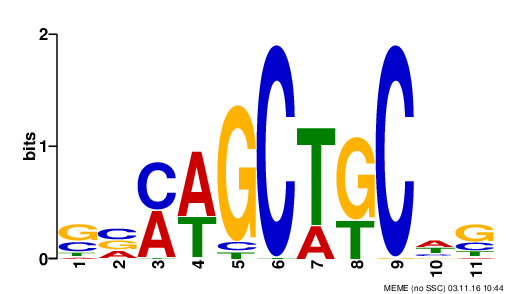

Supplement: S2 Dataset — (GZ) [file pgen.1006619.s020.tar.gz › motif_logo/genic_edits/CT_meme/logo_rc3.png]

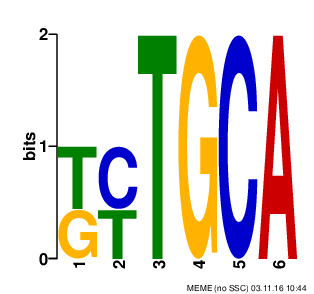

Supplement: S2 Dataset — (GZ) [file pgen.1006619.s020.tar.gz › motif_logo/genic_edits/CT_meme/logo4.png]

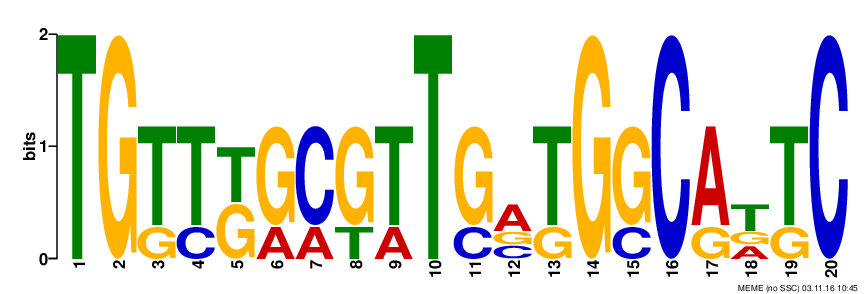

Supplement: S2 Dataset — (GZ) [file pgen.1006619.s020.tar.gz › motif_logo/genic_edits/TA_meme/logo2.png]

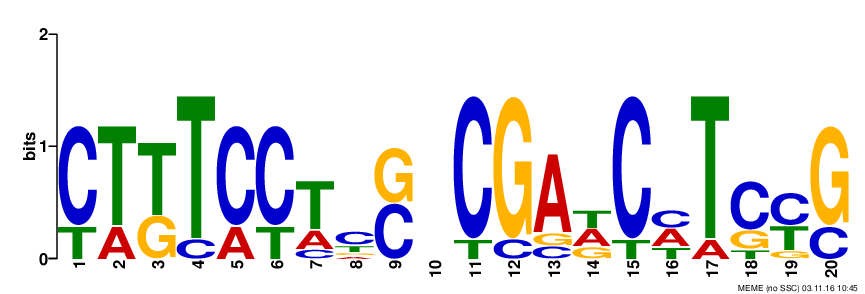

Supplement: S2 Dataset — (GZ) [file pgen.1006619.s020.tar.gz › motif_logo/genic_edits/TA_meme/logo3.png]

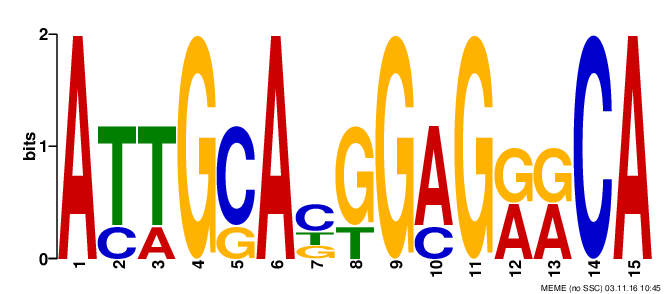

Supplement: S2 Dataset — (GZ) [file pgen.1006619.s020.tar.gz › motif_logo/genic_edits/TA_meme/logo_rc6.png]

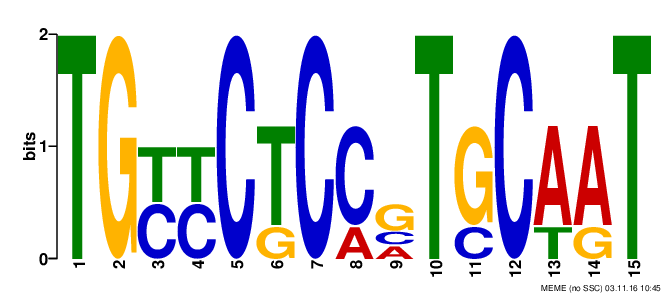

Supplement: S2 Dataset — (GZ) [file pgen.1006619.s020.tar.gz › motif_logo/genic_edits/TA_meme/logo6.png]

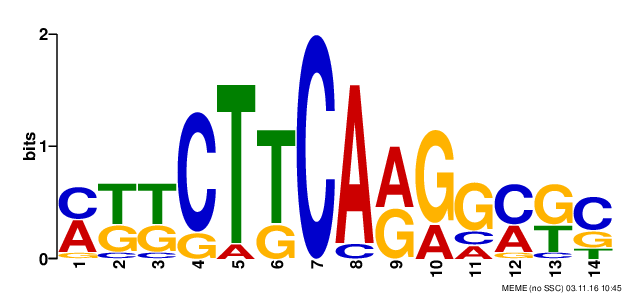

Supplement: S2 Dataset — (GZ) [file pgen.1006619.s020.tar.gz › motif_logo/genic_edits/TA_meme/logo_rc1.png]

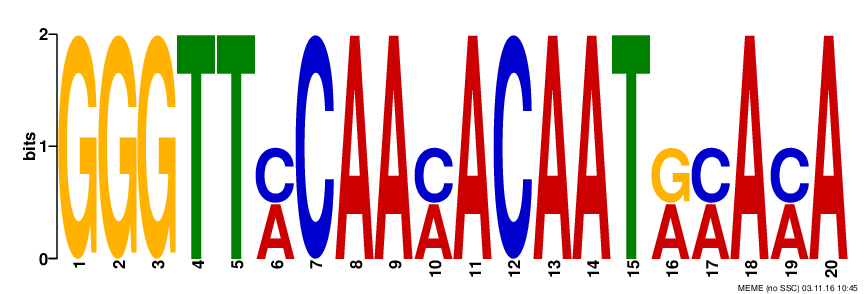

Supplement: S2 Dataset — (GZ) [file pgen.1006619.s020.tar.gz › motif_logo/genic_edits/TA_meme/logo_rc5.png]

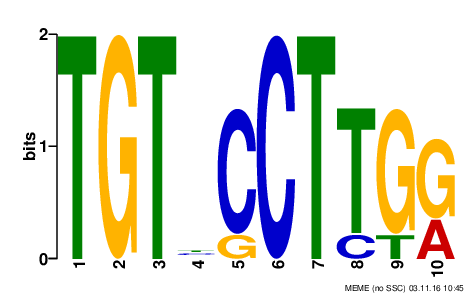

Supplement: S2 Dataset — (GZ) [file pgen.1006619.s020.tar.gz › motif_logo/genic_edits/TA_meme/logo_rc4.png]

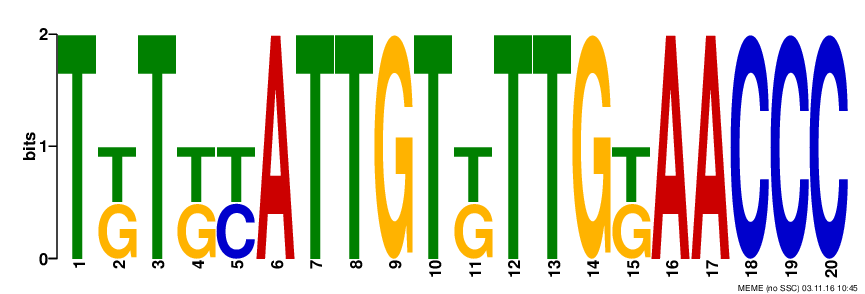

Supplement: S2 Dataset — (GZ) [file pgen.1006619.s020.tar.gz › motif_logo/genic_edits/TA_meme/logo5.png]

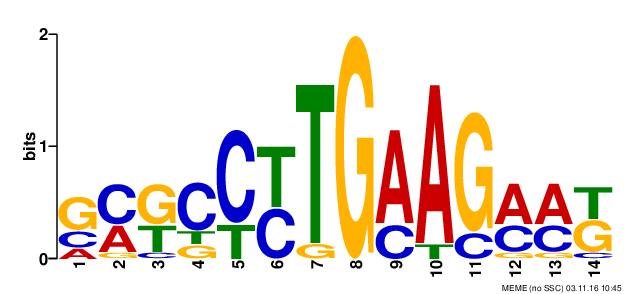

Supplement: S2 Dataset — (GZ) [file pgen.1006619.s020.tar.gz › motif_logo/genic_edits/TA_meme/logo1.png]

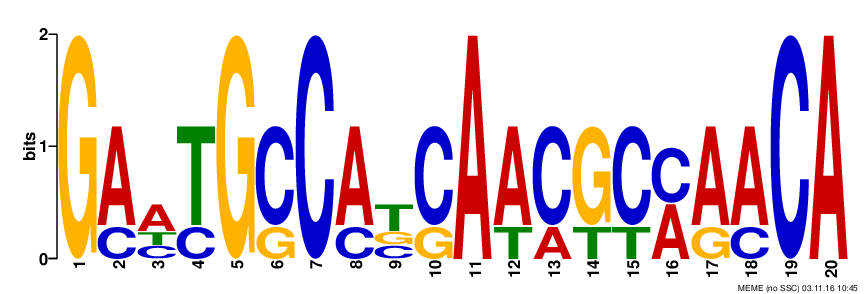

Supplement: S2 Dataset — (GZ) [file pgen.1006619.s020.tar.gz › motif_logo/genic_edits/TA_meme/logo_rc2.png]

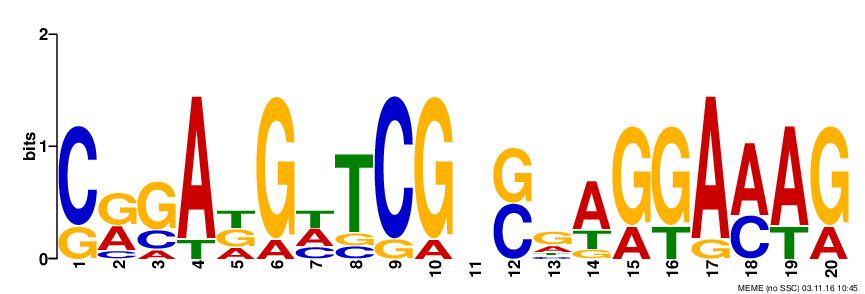

Supplement: S2 Dataset — (GZ) [file pgen.1006619.s020.tar.gz › motif_logo/genic_edits/TA_meme/logo_rc3.png]

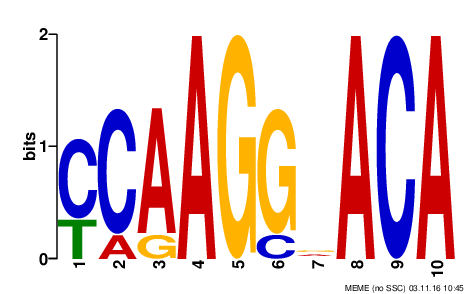

Supplement: S2 Dataset — (GZ) [file pgen.1006619.s020.tar.gz › motif_logo/genic_edits/TA_meme/logo4.png]

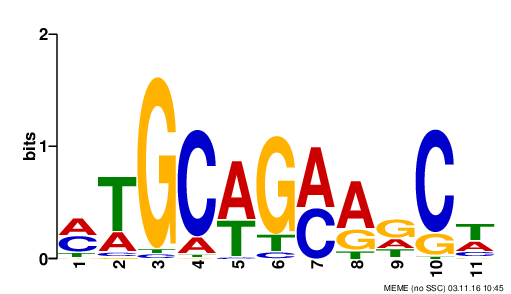

Supplement: S2 Dataset — (GZ) [file pgen.1006619.s020.tar.gz › motif_logo/genic_edits/GC_meme/logo2.png]

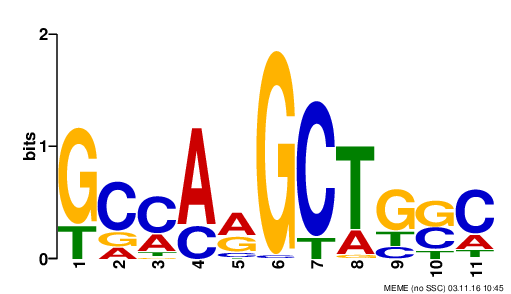

Supplement: S2 Dataset — (GZ) [file pgen.1006619.s020.tar.gz › motif_logo/genic_edits/GC_meme/logo3.png]

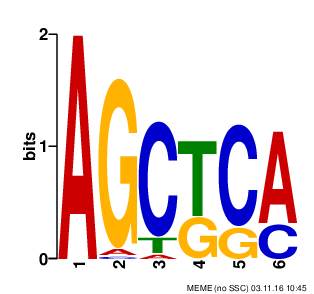

Supplement: S2 Dataset — (GZ) [file pgen.1006619.s020.tar.gz › motif_logo/genic_edits/GC_meme/logo_rc6.png]

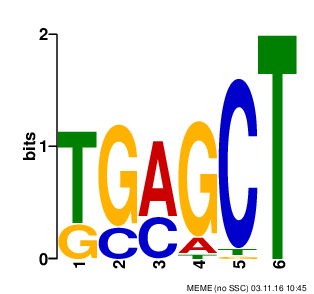

Supplement: S2 Dataset — (GZ) [file pgen.1006619.s020.tar.gz › motif_logo/genic_edits/GC_meme/logo6.png]

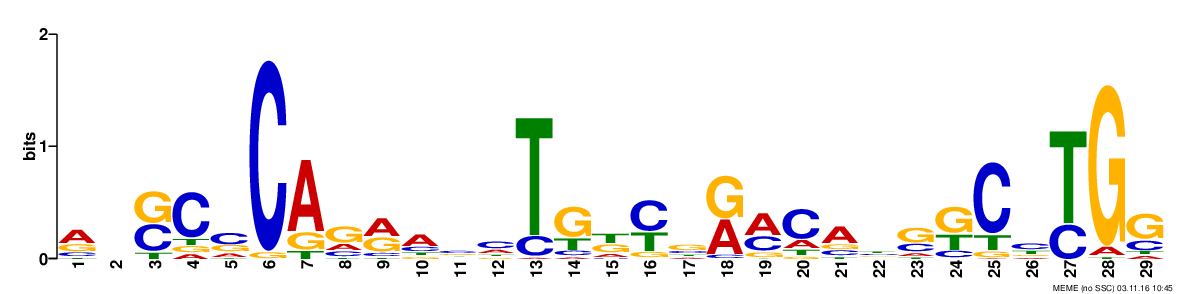

Supplement: S2 Dataset — (GZ) [file pgen.1006619.s020.tar.gz › motif_logo/genic_edits/GC_meme/logo_rc1.png]

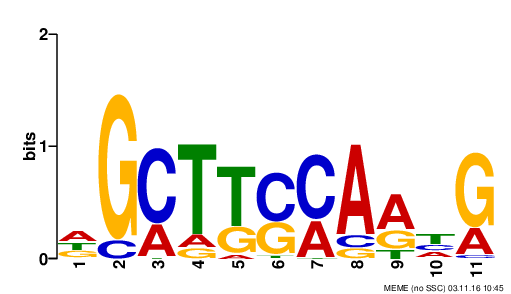

Supplement: S2 Dataset — (GZ) [file pgen.1006619.s020.tar.gz › motif_logo/genic_edits/GC_meme/logo_rc5.png]

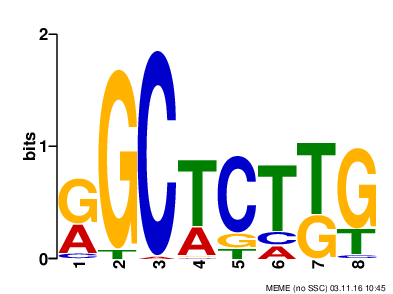

Supplement: S2 Dataset — (GZ) [file pgen.1006619.s020.tar.gz › motif_logo/genic_edits/GC_meme/logo_rc4.png]

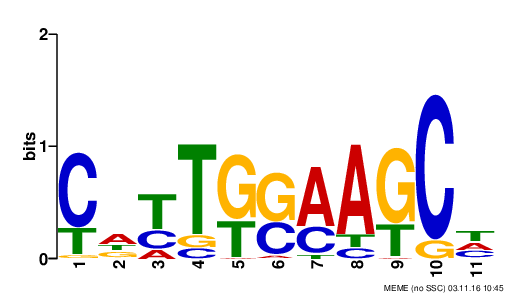

Supplement: S2 Dataset — (GZ) [file pgen.1006619.s020.tar.gz › motif_logo/genic_edits/GC_meme/logo5.png]

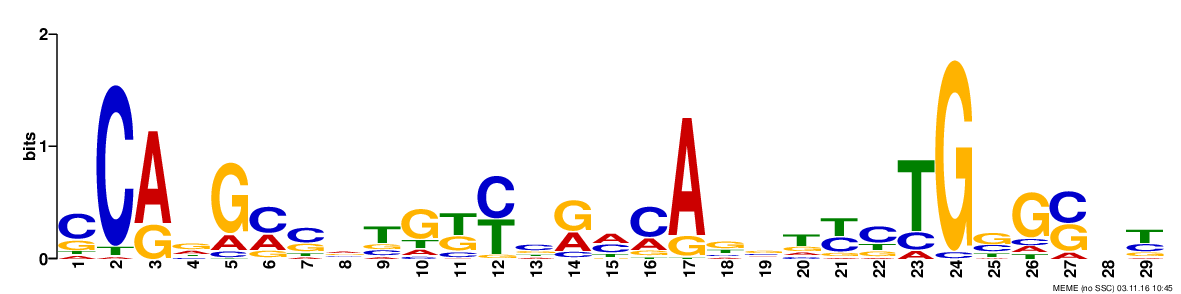

Supplement: S2 Dataset — (GZ) [file pgen.1006619.s020.tar.gz › motif_logo/genic_edits/GC_meme/logo1.png]

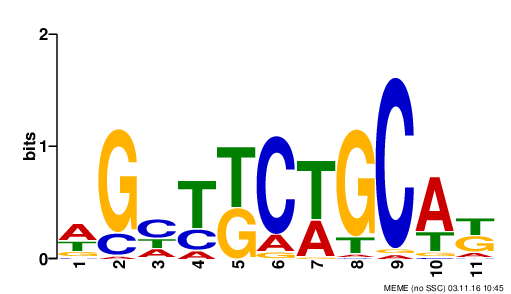

Supplement: S2 Dataset — (GZ) [file pgen.1006619.s020.tar.gz › motif_logo/genic_edits/GC_meme/logo_rc2.png]

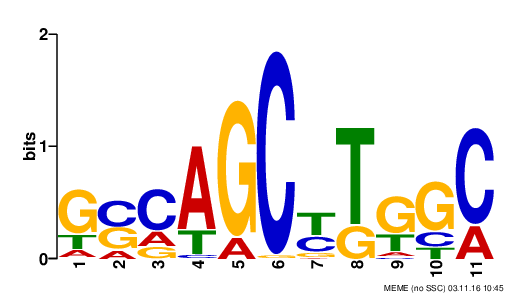

Supplement: S2 Dataset — (GZ) [file pgen.1006619.s020.tar.gz › motif_logo/genic_edits/GC_meme/logo_rc3.png]

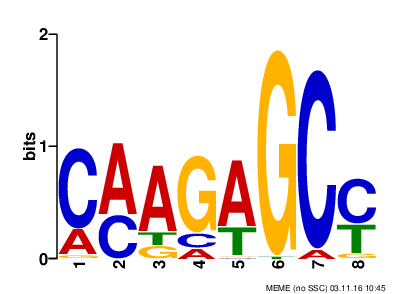

Supplement: S2 Dataset — (GZ) [file pgen.1006619.s020.tar.gz › motif_logo/genic_edits/GC_meme/logo4.png]

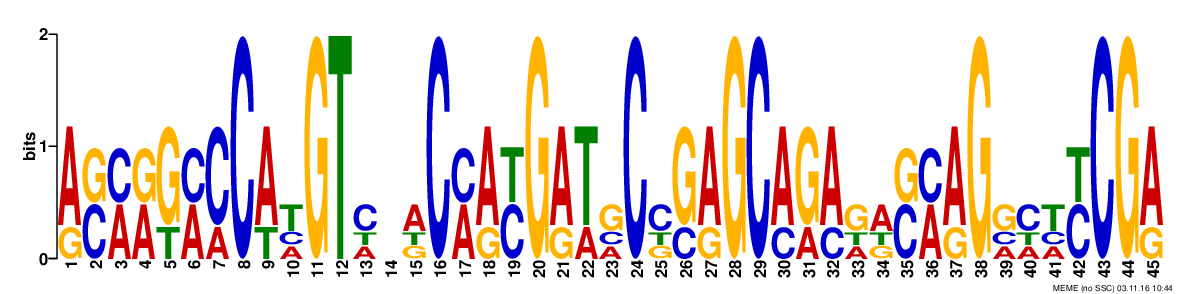

Supplement: S2 Dataset — (GZ) [file pgen.1006619.s020.tar.gz › motif_logo/genic_edits/CA_meme/logo2.png]

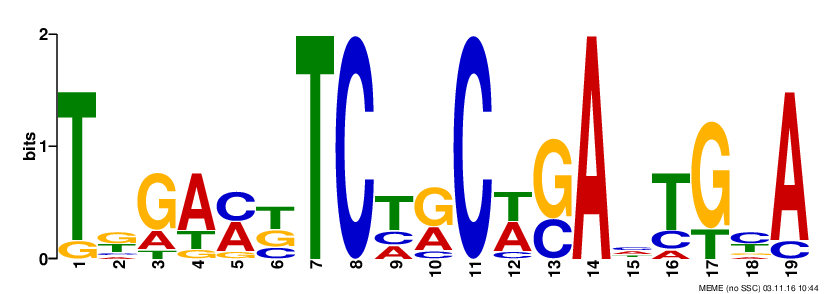

Supplement: S2 Dataset — (GZ) [file pgen.1006619.s020.tar.gz › motif_logo/genic_edits/CA_meme/logo3.png]

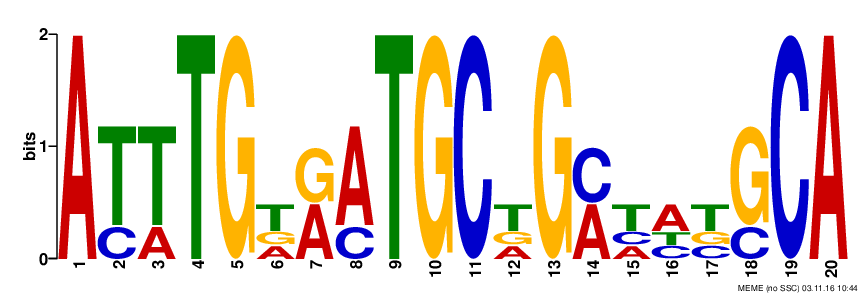

Supplement: S2 Dataset — (GZ) [file pgen.1006619.s020.tar.gz › motif_logo/genic_edits/CA_meme/logo_rc6.png]

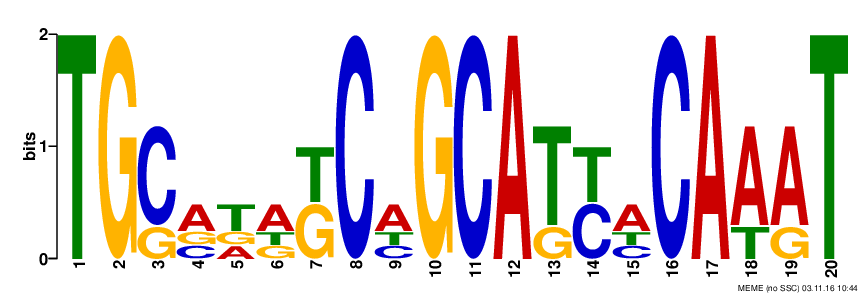

Supplement: S2 Dataset — (GZ) [file pgen.1006619.s020.tar.gz › motif_logo/genic_edits/CA_meme/logo6.png]
